# A Geographically Diverse Collection of *Schizosaccharomyces pombe* Isolates Shows Limited Phenotypic Variation but Extensive Karyotypic Diversity

**DOI:** 10.1534/g3.111.001123

**Published:** 2011-12-01

**Authors:** William R. A. Brown, Gianni Liti, Carlos Rosa, Steve James, Ian Roberts, Vincent Robert, Neil Jolly, Wen Tang, Peter Baumann, Carter Green, Kristina Schlegel, Jonathan Young, Fabienne Hirchaud, Spencer Leek, Geraint Thomas, Anders Blomberg, Jonas Warringer

**Affiliations:** * School of Biology, Queen's Medical Centre, Nottingham, NG7 2UH, United Kingdom; † Departamento de Microbiologia, ICB, C.P. 486, Universidade Federal de Minas Gerais, Belo Horizonte, MG, 31270-901, Brazil; ‡ National Collection of Yeast Cultures, Institute of Food Research, Norwich, NR4 7UA, United Kingdom; § Centraalbureau voor Schimmelcultures, 3584 CT Utrecht, The Netherlands; ** ARC Infruitec-Nietvoorbij, Stellenbosch 7599, Republic of South Africa; †† Howard Hughes Medical institute and Stowers Institute for Medical Research, Department of Molecular and Integrative Physiology, University of Kansas Medical Centre, Kansas City, Missouri 64110; ‡‡ Department of Cell and Molecular Biology, University of Gothenburg, SE-405 30 Gothenburg, Sweden

**Keywords:** pombe, karyotype, diversity, fission yeast

## Abstract

The fission yeast *Schizosaccharomyces pombe* has been widely used to study eukaryotic cell biology, but almost all of this work has used derivatives of a single strain. We have studied 81 independent natural isolates and 3 designated laboratory strains of *Schizosaccharomyces pombe*. *Schizosaccharomyces pombe* varies significantly in size but shows only limited variation in proliferation in different environments compared with *Saccharomyces cerevisiae*. Nucleotide diversity, π, at a near neutral site, the central core of the centromere of chromosome II is approximately 0.7%. Approximately 20% of the isolates showed karyotypic rearrangements as detected by pulsed field gel electrophoresis and filter hybridization analysis. One translocation, found in 6 different isolates, including the type strain, has a geographically widespread distribution and a unique haplotype and may be a marker of an incipient speciation event. All of the other translocations are unique. Exploitation of this karyotypic diversity may cast new light on both the biology of telomeres and centromeres and on isolating mechanisms in single-celled eukaryotes.

Variation segregating in natural populations of model organisms is now widely studied to address questions about the populations structure (Ruderfer *et al.* 2006; Tsai *et al.* 2008), geographic differentiation (Johnson *et al.* 2004), and phylogeny, as well as to provide new sources of variation for the investigation of traits that were previously studied using either experimentally induced mutations or a small set of natural variants (Nieduszynski and Liti 2011). In the case of the budding yeast *Saccharomyces cerevisiae*, variation has, on one hand, been used to establish the origins and relatedness of the many yeast strains (Fay and Benavides 2005; Schacherer *et al.* 2009; Liti *et al.* 2009) that have been exploited by humans and, on the other, to identify new components of pathways and processes that have been exhaustively studied by laboratory methods (Torabi and Kruglyak 2011; Parts *et al.* 2011). In contrast; variation in *Arabidopsis thaliana* (Nordborg and Weigel 2008) has been studied to understand the population structure of a predominantly self-fertilizing plant (Kim *et al.* 2007; Fournier-Level *et al.* 2011), to identify variation in traits of fundamental and agricultural interest, such as disease resistance (Atwell *et al.* 2010; Nemri *et al.* 2010); and to serve as an ecological model that allows a unified understanding of trait variation at the population and nucleotide levels (Todesco *et al.* 2010).

The fission yeast *Schizosaccharomyces pombe* (Egel 2003) is a powerful complement to *S. cerevisiae* for the study of eukaryotic cell autonomous processes. *S. pombe* was originally isolated from East African beer, but subsequently it has been found in many parts of the world in indigenous fermentations, fruit, molasses, and industrial glucose. There have been two large efforts to isolate *S. pombe* from fruit, nectar, or fermentations: one by Florenzano *et al.* (1977) in the vineyards of Western Sicily, and the other by Gomes *et al.* (2002) in four regions of Southeast Brazil. Thus, while *S. pombe* has played only a minor role in biotechnology in comparison to *S. cerevisiae*, isolates of *S. pombe* are present in many of the major yeast strain collections. Although *S. cerevisiae* was intensively studied by brewers, oenologists, and bakers prior to its exploitation as a laboratory model, *S. pombe* has only been studied in any depth as a model of eukaryotic cell biology. Almost all of this work exploits derivatives of the strain 968, which was first identified in a French wine by Osterwalder and then developed as a genetic system by Leupold (1950) (Jürg Kohli, personal communication). Little is known, therefore, about the extent or nature of the variation of this yeast in nature. We assembled a collection of 81 isolates from many regions of the world. We analyzed the isolates at both the phenotypic, genotypic, and karyotypic level. We identified inherited variation in cell size but only limited variability in the proliferative ability in various environments. There are extensive karyotypic differences between many of the strains. The level of nucleotide variation, π, at neutral sites is about 0.7%, which is higher than *S. cerevisiae* (Liti *et al.* 2009). Our data suggest that *S. pombe* exists in small, incompletely isolated populations and that these occupy a limited range of environments. Although mechanistic analysis of the phenotypic diversity of *S. pombe* will require structural analysis of the different karyotypes, the diverse karyotypes may themselves provide new insights into centromere and telomere function, isolation mechanisms, and speciation in single-celled eukaryotes (Gordon *et al.* 2011).

## Materials and Methods

### Handling of strains

Isolates typically arrived on agar slopes or as freeze-dried samples. If they were freeze dried, then the yeast was reconstituted with water and streaked onto supplemented yeast extract agar plates; (YES; 5% yeast extract, 3% glucose, 225 mg/L histidine, 225 mg/L adenine, 225 mg/L leucine, and 225 mg/L uracil), 2% agar Bacto agar (Becton Dickinson). Strains were maintained either on YES agar or cultured in liquid YES.

### Restriction site mapping and PCR

Filter transfer, hybridization analysis, and pulsed field gel electrophoresis were carried out as previously described (Brown 1988; Brown *et al.* 1990). PCR was carried using Taq polymerase (homemade or from Yorkshire Biosciences). Sanger sequencing was carried out using BigDye v3.1 (Applied Biosystems). Primers used to construct probes for filter hybridization are given in supporting information, Table S3.

### DNA extraction, sequencing, and analysis

DNA for PCR was extracted from the 5 mL of yeast cultures using a protocol kindly supplied by Jacob Dalgaard of the Marie Curie Research Institute. Five milliliters of saturated culture was concentrated by centrifugation; spheroplasted using zymolyase 20T in 100 μl 1M sorbitol and 50 mM EDTA; concentrated by centrifugation once again; resuspended in 0.2 mL of DNAzol; and vortex mixed. The DNA was precipitated with an equal volume of cold ethanol. The crude DNA was treated with ribonuclease and then pronase in 10 mM Tris-HCl (pH 8.0), 1 mM EDTA, and 0.1% SDS; extracted between three and five times with a 1:1 mixture of phenol and chloroform; and finally precipitated with ethanol prior to use. Primers used to amplify and sequence DNA for diversity analysis are listed in Table S4.

DNA was amplified with Taq polymerase in an ammonium chloride buffer containing 2 mM MgCl_2_ using the following conditions: an initial denaturation step of 92° for 30 sec was followed by 33 cycles of 92° for 10 sec, 56° for 10 sec, and 65° for 2 min. Primers and unincorporated dNTP were removed from the reactions using Ampure (Agencourt, Beckman Coulter), the products were sequenced on each strand using the primers described above using BigDye (v2 or v3.1; Applied Biosystems), and then purified prior to electrophoretic analysis by Cleanseq (Agencourt, Beckman Coulter). Sequences were aligned and edited using Bioedit and collapsed into haplotypes using FaBox (http://www.birc.au.dk/~biopv/php/fabox/). Sequences that defined unique haplotypes at any one locus were reamplified and resequenced. Standard summary statistics were extracted with Arlequin (Excoffier *et al.* 2005) and DNASp (Librado and Rozas 2009). DNA for pulsed field gel electrophoresis was embedded in agarose plugs and extracted as described (Smith *et al.* 1987).

### Microarray analysis

For microarray analysis, chromosomal DNA was size-fractionated by pulsed field gel electrophoresis, electroeluted from the gel into dialysis tubing, concentrated using Butan-2-ol, amplified by the Qiagen REPL1-g mini kit before labeling using the Agilent DNA ULS labeling kit (5190-0419), and then purified and hybridized to the Agilent *S. pombe* 4 × 44K ChIP-on-chip array (G4810) using unfractionated CRUK 972 DNA as competitor. In some experiments, unfractionated DNA from natural isolates was used as target. All steps were carried out according to the manufacturer’s instructions. Arrays were scanned using an Agilent Scanner, and the data were analyzed using the Agilent Genomic Workbench v5.0.14.

### Quantifying natural trait variation

Strains were subjected to high-throughput phenotyping by microcultivation (n = 2) in an array of environments essentially as described (Warringer *et al.* 2008; Warringer and Blomberg 2003). Briefly, strains were inoculated in 350 µl of YES medium (5% yeast extract, 3% glucose, 225 mg/L histidine, 225 mg/L adenine, 225 mg/L leucine, and 225 mg/L uracil) and incubated in two serial rounds of precultivation for 48–72 h at 30°. For experimental runs, strains were inoculated to an OD of 0.05–0.1 in 350 µl of YES medium (3% glucose was replaced by 3% of alternative carbon sources where indicated) and microcultivated for 48 h or 72 h in a Bioscreen analyzer C (Oy Growth Curves, Finland). Optical density was measured every 20 min using a wide band (450–580 nm) filter. The mitotic proliferation rate (population doubling time), lag (population adaptation time), and efficiency (total change in population density) were extracted from high-density growth curves and LN (natural logarithm)–transformed (data set in Table S5). Relative fitness variable for each strain and trait, LSC*ij*, was calculated as:

$${\text{LSC}}_{ij}=\frac{\sum_{r=1}^{2}\left[\left[\frac{1}{10}\sum_{k=1}^{10}\log\left(wt_{kj}^{r}\right)\right]-\log\left(x_{ij}^{r}\right)\right]}{2}$$

where *wt_kj_* is the fitness variable of the *k*^th^ measurement of the wild-type for trait *j*; *x_ij_* is the measure of strain *i* for trait *j*; and *r* indicates the run. The measure for proliferation efficiency was inverted to maintain directionality between fitness components.

### Cell size at septation

Cells were cultured over night in YES containing glucose at 0.5% weight per volume until they reached mid-log phase, concentrated by centrifugation, and visualized using phase microscopy using a Zeiss Axioscop microscope with a Plan NeoFluor 40× objective. Images were collected and sizes were quantitated using Metamorph (Universal Imaging) v6.1.

### Population structure revealed by F_st_ analysis of concatenated sequences of noncoding DNA

The SPBC660.16 large intron (which encodes phosphogluconate dehydrogease), TER1, and CEN2 sequences were stripped of indels and, in the case of the large intron of SPBC 660.16, the microsatellite; concatenated; and then grouped according to geographic origin. F_st_ values (Cockerham and Weir 1984) were estimated using the Arlequin package (Excoffier *et al.* 2005).

### Linkage disequilibrium calculation

Informative SNPs were identified both in the centromere of chromosome 2 (Figure S6) and in the flanking DNA (Figure S7) and in the TER1 gene and in the flanking DNA (Figure S8) and used to estimate linkage disequilibrium (LD) statistics using DNAsp (Table 5).

## Results

### A collection of natural isolates of *S. pombe*

**Figure 1:**
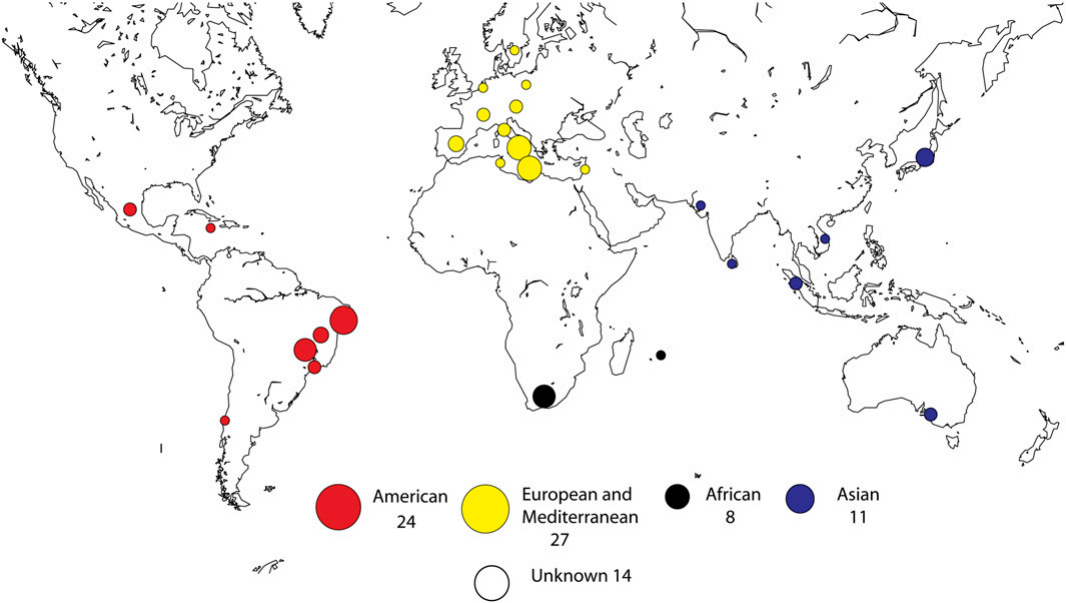
Geographic origin of the 84 strains of *Schizosaccharomyes pombe* used in this study. The area of the circles is proportionate to the numbers of strains from the respective areas.

We started our project by assembling a collection of 84 *S. pombe* strains (Figure 1, Table 1, and Table S1). Of these strains, 81 were natural isolates and 3 were listed as laboratory strains. We first checked the ploidy by DNA staining with Cytox green and fluorescence flow cytometry; this showed that all of the strains but one (NCYC 2355) were haploid. We therefore subcloned NCYC 2355 and isolated three haploid clones. We analyzed two of these clones using the methods described below; they were identical, so we refer to one: NCYC 2355-1. We characterized the 84 haploid strains by sequence and phenotype. We initially sequenced three segments of the genome that did not code for proteins: a segment of the central core of chromosome II (II: 1,621,085–1,621,800); the gene encoding the RNA component of telomerase, TER1 (I: 3,084,446–3,086,143); and the second intron (the largest intron in the *S. pombe* genome, II: 230,740–231,501) of gene SPBC660.16-1, which encodes phosphogluconate dehydrogenase. To establish a preliminary estimate of the levels of outcrossing (see below), we also sequenced two loci that flanked the centromere of chromosome II (II: 1,572,330–1,572,988 and 1,658,033–1,658,751) and two loci centromere distal of the TER1 gene (I: 3,113,975–3,114,540 and I: 3,194,538–3,195,201). In total, we sequenced and analyzed 5,777 bp in 84 strains. The results established that many of the isolates were closely related and that the entire collection could be reduced to 40 identical haplotypes (Table 1). The strains with identical haplotypes either had a widespread distribution or were collected close to one another. Haplotypes with a widespread distribution could have spread around the world by natural mechanisms or as result of human activity. The geographically restricted haplotypes may represent clones that had reached a high frequency because of founder effects or population bottlenecks, or it may have arisen from restricted collection activity.

**Table 1:** Assorting 84 strains of *S. pombe* into 40 groups with shared haplotypes at seven loci

Strain	Haplotype Number	Origin Where Known
UWOPS 92.229.4	1	Mexico
UWOPS 94.422.2	2	Mexico
UFMG A529, UFMG 790, UFMG A826	3	Brazil, Belo Horizonte and Viçosa
UFMG R416, UFMG R418, UFMG R420, UFMG R424, UFMG R435	4	Brazil; Aracaju
UFMG R427	5	Brazil; Aracaju
UFMG A1263	6	Brazil, Viçosa
UFMG A521, UFMG A571, UFMG A602	7	Brazil, Belo Horizonte
UFMG A1000, UFMG A1153,	8	Brazil, Belo Horizonte and Salinas
UFMG R434	9	Brazil; Aracaju
UFMG R428	10	Brazil; Aracaju
UFMG A1152	11	Brazil, Salinas
UFMG R437	12	Brazil; Aracaju
UFMG A738	13	Brazil, Belo Horizonte
NCYC 683, NCYC 2387, DBVPG4435, AWRI 442	14	Spain, Italy, South Australia
NCYC 936, NCYC 2355-1,	15	Sri Lanka, Japan
CBS 356, NCYC 132, NCYC535, DBVPG2817, DBVPG4437, AWRI 141	16	Eastern Mediterranean, Africa, Italy, Australia
NCYC 380, CBS 1063, DBVPG 6281, CBS 355, DBVPG 6417	17	Sicily, Spain
DBVPG 4433, DBVPG 6279, DBVPG 6610, DBVPG 6699, Y0036, Y0037, CRUK 972, CRUK 975, Y 468, Y 469,	18	Germany, Indo-China, South Africa, France,
CBS 2628	19	Pakistan
CBS 2775, CBS 2776, CBS 2777	20	Japan (all)
CBS 5680	21	Poland
CBS 5682	22	South Africa
CBS 7335	23	Spain
DBVPG 2801	24	Tunisia
DBVPG 2805	25	Malta
DBVPG 2804, DBVPG 2806, DBVPG 2807, DBVPG 2808, DBVPG 2809	26	Malta (all)
DBVPG 2810	27	Malta
DBVPG 2811, DBVPG 2812, DBVPG 2814, DBVPG 2815, DBVPG 2816, DBVPG 2818	28	Sicily (all)
Y470	29	—
Y 831, Y 832	30	South Africa (both)
CBS 374	31	Delft
DBVPG 6447, DBVPG 6449	32	—
CBS 358	33	—
CBS 1058	34	Java
CBS 357	35	Jamaica
CBS 352	36	Indonesia
CBS 1057	37	Sweden
CBS 1059	38	Mauritius
CBS 1044	39	—
L2470	40	Chile

Each of the 84 strains in the original collection was sequenced using conventional Sanger methodology at seven individual loci as described in the text and in File S1. Strains were grouped according to 40 compound haplotypes. For the details of the sequences of the individual loci and the haplotype structures, see File S1.

### Population genetics of *S. pombe*

We used sequence data to analyze the diversity and population structure of *S. pombe*. To minimize the consequences of biased collecting activities or human trade, we carried out this analysis using the diversity contained within the 40 different haplotypes identified in Table 1 and Table S2. The diversity π (Table 2) detected at each of the three nonprotein-coding loci varies with the highest value being observed at the locus most likely to approach neutrality, the central core of the centromere of chromosome II. A negative value of Tajima’s D suggests recent selection at or close to the region of the SPBC 660.16 intron. The value of π of 7 × 10^−3^ seen at the central core of chromosome II is consistent with the variation seen in pairwise comparison of 4-fold degenerate sites between the laboratory strain and strains NCYC 132 and SPK 1820, which gave values of 8.9 × 10^−3^ and 6.7 × 10^−3^ (Rhind *et al.* 2011), respectively, so we concluded that this estimate is correct. This is slightly higher than in the budding yeast *S. cerevisiae*, which has a value of 5.65 × 10^−3^ (Liti *et al.* 2009). The fact that all but one of the strains are haploid and the assumption of a neutral coalescent allows estimation of the effective mitotic population size N_e_ by use of relationship π = 2N_e_u, where u is the mutation rate per nucleotide per mitotic generation (Tsai *et al.* 2008). The neutral mutation rate in *S. pombe* is not known, so we assumed the rate that has been used for *S. cerevisiae* of 0.33 × 10^−9^/bp/generation (Lynch *et al.* 2008), leading to an estimate of the global effective population size of 1 × 10^7^. It is also of interest to know how much variation is endemic to particular population. There was too little sequence variation to be able to estimate population structure *a priori* using the Bayesian approaches implemented in the STRUCTURE (Pritchard *et al.* 2000) and BAPS (Corander *et al.* 2008) packages, so we grouped the haplotypes according to their geographic origin, where this was possible, and then analyzed them using the F statistic (Cockerham and Weir 1984). These results (Table 3) demonstrated little by way of population structure but suggested that the American strains were the most highly differentiated. Network analysis of the CEN2, TER, and 660.16 sequences was also consistent with extensive mixing between the different haplotypes (Figure S6, Figure S8, Figure S9, and Figure S10). Two loci include sufficient data to estimate the extent of linkage disequilibrium, which is inversely proportional to the out-crossing rate. To do this, we identified informative SNPs in and around the centromere of chromosome II and within and centromere distal of the TER1 gene (Figure S6, Figure S7, Figure S8, Figure S9, and Figure S10). A four gamete test of the sequence variation around the centromere does not indicate recombination. Significant linkage disequilibrium is also detectable 30 kb from the telomerase RNA gene using both the D' and R statistics (Table 4). The extent of linkage disequilibrium would seem, therefore, to be greater than in *S. paradoxus* (Tsai *et al.* 2008), which out-crosses once in every 1000 asexual generations and which shows lower levels of nucleotide diversity. Network analysis (Figure S6, Figure S9, and Figure S10) of the haplotypes at centromere II, TER1, and SPBC660.16, however, demonstrate that there is extensive mixing between different strains. More detailed analysis will be required to measure the rate of out-crossing in *S. pombe* and to correlate it with the karyotypic diversity described below.

**Table 2:** Nucleotide diversity at three noncoding loci in the genome of *S. pombe*

Sequence	Number of Residues	π ± SD × 10^−3^	θ ± SD × 10^−3^	Tajima’s D (per Sequence)	Number of Segregating Sites
CC CEN2-indels	719	6.997 **±** 3.863	6.866 **±** 2.520	0.01131	21
TER1-indels	1702	4.329 **±** 2.298	4.420 **±** 1.472	−0.08316	32
660.16 intron-minisat	743	1.603 **±** 1.165	3.798 **±** 1.51	−1.77965	12

Diversity statistics were calculated based on the sequences at the three indicated loci using Arlequin. The sequences were cleared of indels and microsatellite sequences prior to analysis.

**Table 3:** Population differentiation as measured by F_st_

Population	American	African	European	Asian	Unknown
American (15)	0				
African (5)	0.075	0			
European (11)	0.087	−0.021	0		
Asian (7)	0.009	0.02	0.035	0	
Unknown origin (9)	0.044	−0.01	0.051	0.022	0

The sequence data from the 40 compound haplotypes were used to estimate pairwise F_st_ measures of population differentiation using the Arlequin package. Haplotypes (*e.g.* haplotypes 16 and 18) containing strains of different origins were considered as being of unknown origin. The numbers of strains in the respective populations are indicated in parentheses. Haplotypes containing strains of different known origins were assigned multiply to different populations and thus the sum of the numbers in brackets exceeds 40.

**Table 4:** Linkage disequilibrium around centromere 2 and telomerase RNA gene (TER1)

Chromosome II centromere					
R/chi	**1572349**	**1621091**	**1658392**	**1658455**	
1572349		−0.396	−0.597	−0.396	
1621091	6.271^*^		0.664	1	
1658392	14.235^***^	17.622^***^		0.664	
1658455	6.271^*^	40^***^	17.622^***^		
D'	**1572349**	**1621091**	**1658392**	**1658455**	
1572349		−1	−1	−1	
1621091			1	1	
1658392				1	
1658455					
Chromosome I TER1 region					
R/chi	**3084762**	**3086123**	**3114239**	**3114431**	**3194949**
3084762		0.555	0.267	0.434	−0.248
3086123	12.31^***^		0.339	0.236	−0.089
3114239	2.857	4.596^*^		0.85	−0.0207
3114431	7.519^**^	2.222	28.9^***^		−0.333
3194949	2.462	0.32	1.709	1.778	
D'	**3084762**	**3086123**	**3114239**	**3114431**	**3194949**
3084762		0.826	0.331	0.457	−0.0373
3086123			0.407	0.236	−0.2
3114239				1	−0.385
3114431					−0.333
3194949					

Haplotypes at individual loci were identified to identify informative SNPs, and these were used to measure linkage disequilibrium by the indicated metrics using the DNAsp package. The significance of the chi squared values are indicated by asterisks: *, at the 5% level; **, at the 1% level; ***, at the 0.1% level. The numbers in bold at the margins of the table refer to the coordinates along the chromosome of the respective, informative SNPs.

### Trait variation in *S. pombe* is defined by population structure and geographic boundaries

**Figure 2:**
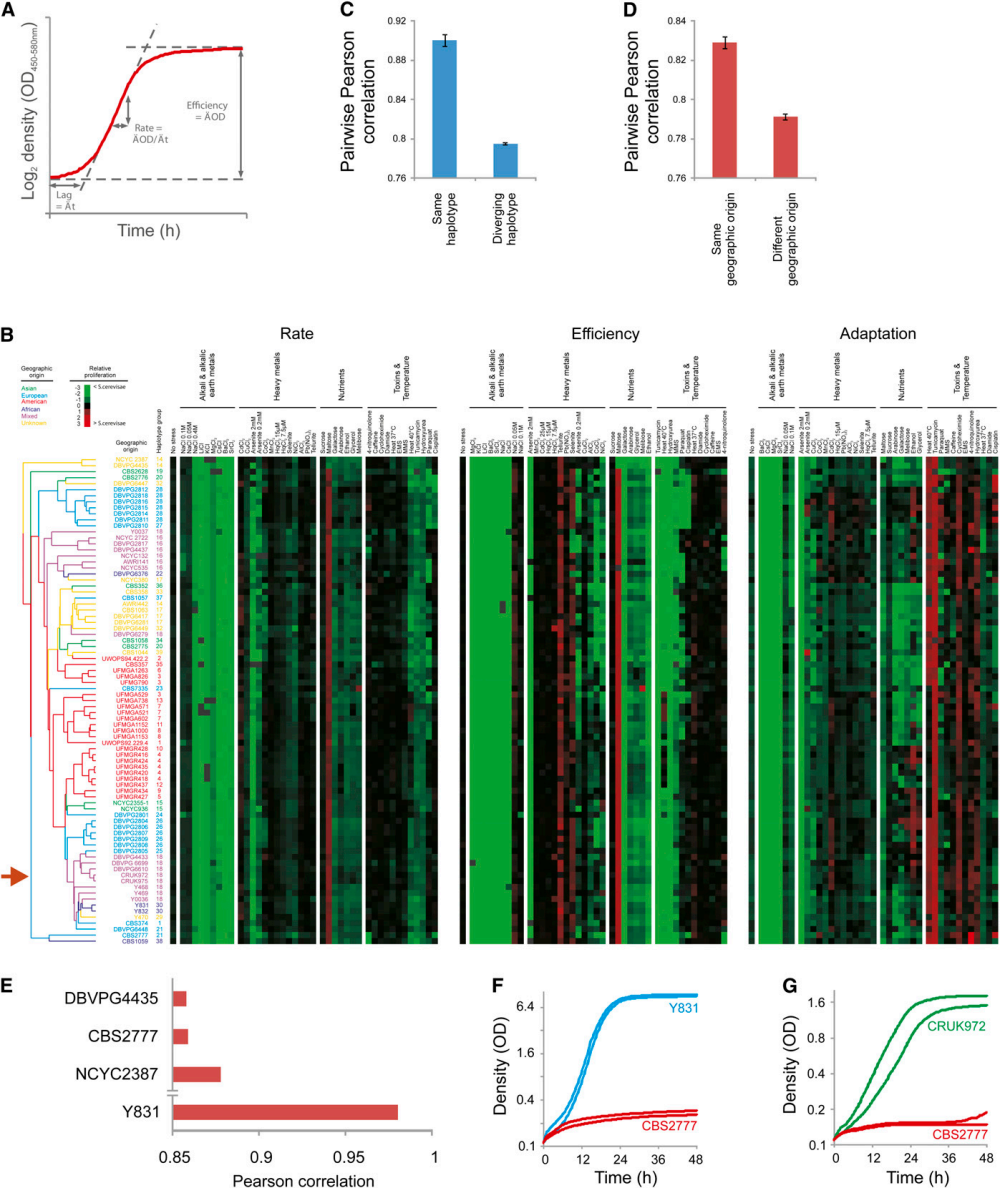
Trait variation in *S. pombe* is defined by population structure and geographic origin. (A) The proliferative lag (time to initiate proliferation), proliferative rate (population doubling time), and proliferative efficiency (change in population density) were extracted from high-density growth curves (n = 2) of natural *S. pombe* isolates (N = 2) over 42 environments. (B) Hierarchical clustering of *S. pombe* natural isolates was performed using trait profiles based on all traits, a centered Pearson correlation metric, and average linkage mapping. Numbers indicate haplotype groups and color indicates geographic origin. Heat map depicts proliferation relative the *S. cerevisiae* universal type strain BY4741 (Log2 [isolate/BY4741]). Green = inferior proliferation, red = superior proliferation, black = BY4741 proliferation, gray = missing data. The red arrow indicates the *S. pombe* reference strains 972 h− and 975 h+. (C) Pearson correlation coefficients were calculated between all pairs of strains belonging to the same and to different haplotype groups. Means and standard errors of the means are displayed. (D) Pearson correlation coefficients were calculated between all pairs of strains with similar and diverging geographic origins. Means and standard errors of the means are displayed. (E) A *S. pombe* mean trait profile was calculated and the similarity (Pearson correlation) between the mean trait profile and the trait profile of each individual isolate was calculated. Isolates were ranked according to degree of similarity; the bottom three (most atypical) *S. pombe* isolates are displayed. The most typical *S. pombe* isolate, Y 831, is shown for comparison. (F, G) Proliferation of the *S. pombe* karyotype extreme CBS 2777 in presence of the DNA-damaging drugs cisplatin (F) and 4-nitroquinolone (G). Strains showing typical *S. pombe* behavior in these environments are included for comparison.

**Figure 3:**
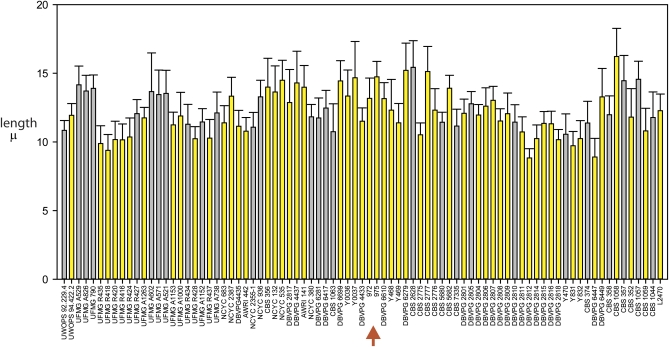
Variation in length at septation among natural isolates of *S. pombe*. *S. pombe* isolates were cultured to mid-exponential phase of growth, harvested, and then analyzed by phase contrast microscopy. The individual isolates are arranged on the abscissa according to their respective haplotypes, with isolates of the same haplotype placed adjacent to one another and accorded the same color (alternating gray or yellow). The red arrow indicates the *S. pombe* reference strains 972 h− and 975 h+.

To survey natural trait variation in *S. pombe*, isolates were subjected to high-resolution quantification of proliferative ability in an array of environments representing variations in nutrient availability, temperature, and exposure to toxic metals and drugs. From high-density mitotic growth curves, the fitness components lag of proliferation, rate of proliferation (population doubling time), and efficiency of proliferation (population density change) were extracted, providing >120 distinct measures of organism-environment interactions (Figure 2). These fitness components were compared with those of the *S. cerevisiae* universal type strain BY4741. In optimal conditions, *S. pombe* featured a delay in the time to initiate population growth and a marginal reduction in proliferation rate relative *S. cerevisiae* (Figure S1, A and B). In contrast, the performance of *S. pombe* was very severely impaired in many stress-inducing environments (Figure 2B). In particular, *S. pombe* proliferation in the presence of alkali and alkaline earth metals, DNA damaging agents, and respiratory or partially respiratory carbon sources was reduced (Figure 2B and Figure S1D). Utilization of maltose, the main storage carbohydrate of barley, represented an exception, being more efficient in *S. pombe* (Figure S1, D and E). Overall, trait variation in *S. pombe* was lower than what has been reported both for the variable *S. cerevisiae* and its less variable wild relative *S. paradoxus* (Warringer *et al.* 2011) (Figure S2A). This suggests that *S. pombe* is adapted for proliferating in a more constrained ecological range than the baker’s yeasts. Nevertheless, trait variation largely followed phylogenetic boundaries as defined by haplotype structure and geographic origin: strains with the same haplotype or the same geographic origin were significantly (Student *t*-test, *P* < E−34) more similar than other strains (Figure 2B–D). In addition, we surveyed cell length at septation and found the influence of the haplotype structure to prevail for this trait (Figure 3). The most phenotypically distinct *S. pombe* populations were the American population, with an impaired proliferation at high temperatures, and the European population, with a prolonged lag phase when initiating respiratory proliferation as well as elevated tolerance to DNA damage (Figure S2B). The strain CBS 2777 stands out in terms of mitotic fitness traits, and it emerges as one of the most atypical strains (Figure 2E and Figure S3A). CBS 2777 is a strain with the unusual karyotype of four chromosomes (see below). CBS 2777 deviated from the *S. pombe* mean in more than 14% of all traits (Student *t*-test, FDR 5%). Most of these abnormalities represented severe proliferation deficiencies, notably the inability to tolerate DNA-damaging drugs, such as cisplatin and 4-nitroquinolone, that strain DNA replication and repair (Figure 2, G and F and Figure S3B). These abnormalities were evident also in relation to CBS2775 and CBS2776, which share haplotype, but not karyotype, structure with CBS2777.

### Extensive karyotypic diversity in *S. pombe*

**Figure 4:**
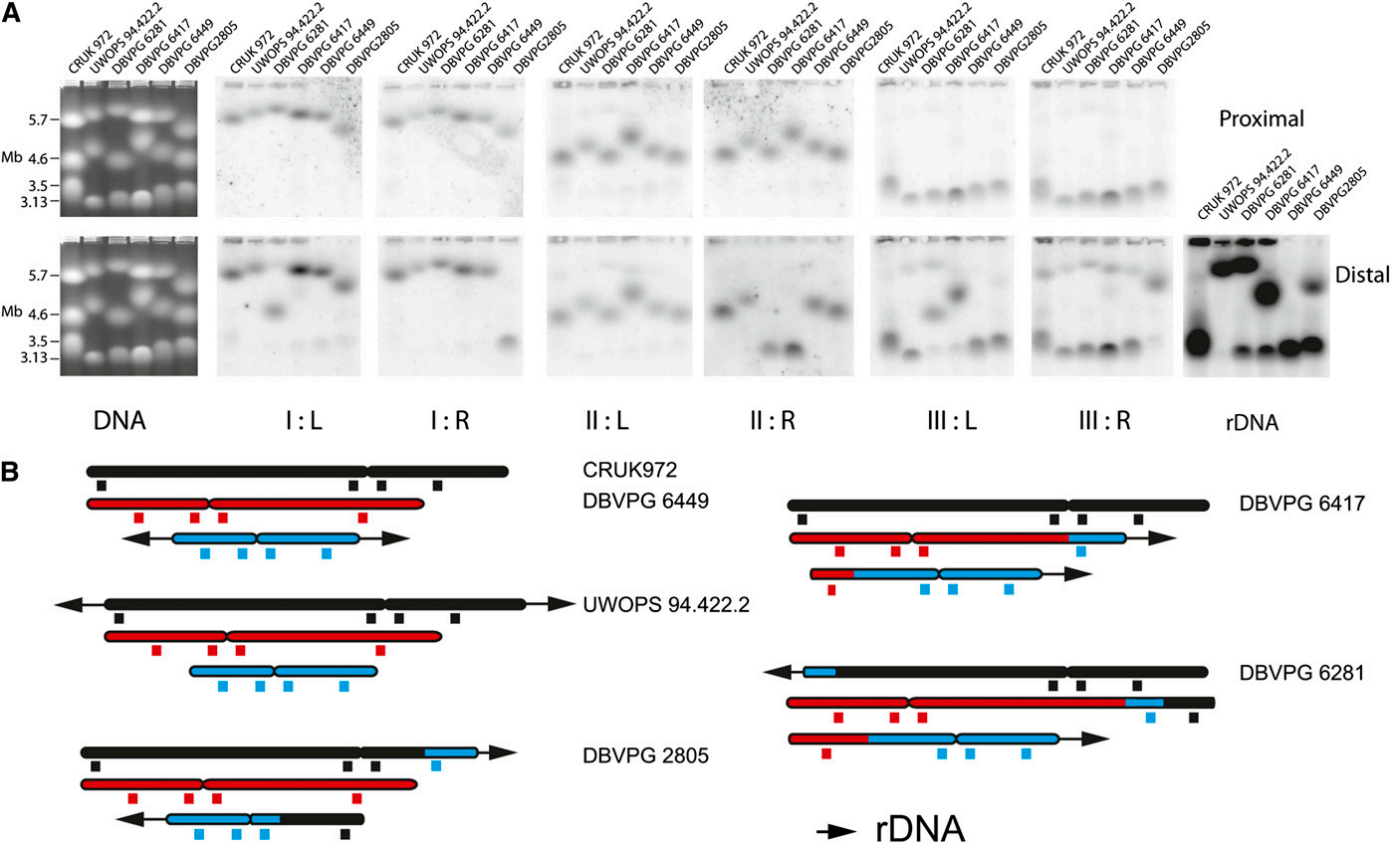
Rearrangement of the *S. pombe* karyotype by translocations of the rDNA and associated sequences. (A) DNA extracted from five different natural isolates (UWOPS 94.422.2, DBVPG 6281, DBVPG 6417, DBVPG 6449, and DBVPG 2805) and from laboratory strain CRUK 972 was size-fractionated by pulsed field gel electrophoresis and then analyzed by filter hybridization with centromere proximal and distal probes from each of the six arms of the three chromosomes and with a probe derived from rDNA. The figure illustrates the original ethidium bromide-stained gels and the results of the filter hybridization. Four isolates had karyotypes in which the rDNA was rearranged. In UWOPS 94.422.2, the rDNA is translocated in its entirety onto chromosome I. In DBVPG 2805, the rDNA on the right arm of the laboratory strain chromosome III is translocated together with the distal IIIR sequence onto chromosome I, and distal IR sequence is translocated onto chromosome III. In DBVPG6417, distal IIIL sequences are translocated onto chromosome II, and distal IIR sequences are translocated onto chromosome III. DBVPG 6281 appears to be a derivative of DBVPG 6417 as the rDNA is now present on chromosome I and distal IL sequences are present on chromosome II. (B) An ideogrammatic interpretation of these results together with an indication of the approximate positions of the probes used in the filter hybridization.

**Figure 5:**
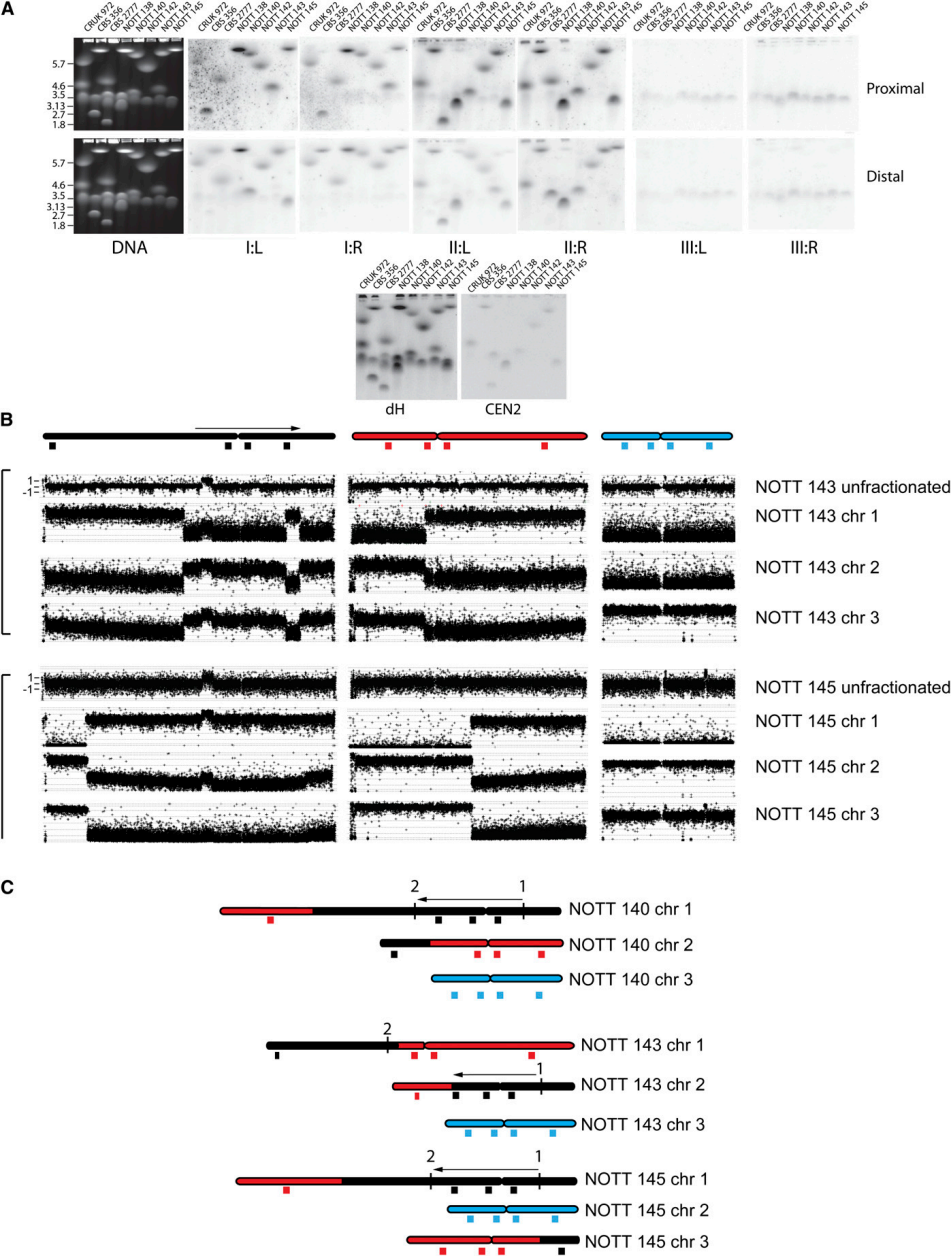
Rearrangement of the *S. pombe* karyotype by translocations between chromosomes I and II. (A) DNA extracted from seven different natural isolates (CBS 356, CBS2777, NOTT 136, NOTT 140, NOTT 142, NOTT 143, and NOTT 145) and laboratory strain CRUK 972 was size-fractionated by pulsed field gel electrophoresis and then analyzed after transfer by filter hybridization with centromere proximal and distal probes from each of the six arms of the three chromosomes and with probes for the centromeric dGdH repeat and the central core of chromosome II. The figure illustrates the original ethidium bromide-stained gels and the results of the filter hybridization. (B) DNA was extracted from strains NOTT 143 and NOTT 145, size fractionated by pulsed field gel electrophoresis, and analyzed together with unfractionated DNA by CGH using Agilent 44K ChIP on CHIP arrays using unfractionated DNA from the laboratory strain CRUK 975 as competitor. In the case of NOTT 145, chromosomes 2 and 3 are very similar in size; there is cross contamination in the CGH experiments, but this does not obscure the details of the translocation between sequences that normally reside on chromosomes I and II. (C) Ideogrammatic representation of the CGH results together with an indication of the approximate positions of the probes used in the filter hybridization. Also indicated is the position of the 2.3 Mb pericentric inversion of sequences with respect to laboratory strain chromosome I. This inversion is present in both NOTT 143, as indicated by the results of the CGH experiments, and NOTT 143, as indicated by the results of PCR analysis. For a detailed discussion of the experimental approaches needed to define the rearrangements, see File S1.

The laboratory strain of *S. pombe* possesses three chromosomes. Each centromere includes two blocks of tandemly repeated heterochromatic sequences that are arranged palindromically around an A+T rich central core. There is extensive sequence homology between the six blocks of tandemly repeated centromeric DNA, suggesting the possibility of polymorphism arising either from exchange between the arrays of repeats at different centromeres or from instability of individual centromeric palindromes. In light of this potential polymorphism, we analyzed the chromosomes by pulsed field gel electrophoresis, filter transfer, and hybridization using a set of single-copy probes that lie centromere proximal and distal on the six arms of the laboratory strain karyotype and with a probe for the ribosomal DNA (rDNA), which occupies a subtelomeric position at the ends of the two arms of chromosome III. Simple ethidium bromide staining of the pulsed field gels showed that four African isolates (Y468, Y470, Y831, and Y832) were mixtures of different karyotypes and were subcloned. One of the isolates (CBS 374), which gave ambiguous results upon first pulsed field gel analysis, was also subcloned. Together these subclones defined 15 additional strains, which we define as NOTT 133–145, NOTT 147, and NOTT 148. We subsequently sequenced these subclones at the most informative (as defined by number of haplotypes) of the seven loci that we had sequenced in the original set of 84 strains (SPBC660.0.16, TER1, and the central core of the centromere of chromosome II), and we confirmed that they were isogenic with respect to the original uncloned isolates. The pulsed field gel and filter hybridization analysis identified three types of gross chromosome rearrangement. Four isolates (UWOPS 94.422.2, DBVPG 2805, DBVPG 6417, and DBVPG 6281) had karyotypes in which the rDNA was rearranged. The nature of these rearrangements was analyzed as shown in Figure 4A to produce the ideograms shown in Figure 4B. Each of these rearrangements was unique in the collection. The conclusion we draw is that *S. pombe* shows extensive exchange of subtelomeric DNA sequences, including rDNA. It is striking that the sequences that are exchanged between the chromosomes extend hundreds of kilobases into single-copy, chromosome-specific DNA. It is also notable that there is extensive size variation between chromosomes not detectably involved in the translocation of the rDNA arrays. The cause of this variation remains to be determined but may include translocations and exchanges of subtelomeric DNA that our analyses failed to detect. The second type of rearrangement involved sequences normally present on chromosomes I and II of the laboratory strain. To facilitate discussion, we refer to these types of rearranged chromosomes using Arabic numerals, with the largest chromosome in any one strain termed chromosome 1. We identified six different rearrangements of this type. One of these rearrangements was present in what is referred to as the *S. pombe* type strain CBS 356 (Figure 5A) and in five other strains (NCYC 132, NCYC 535, DBVPG 2817, DBVPG 4437, and AWRI 141) that have a widespread distribution over the old world and Australia (Table 1, Figure S4). These strains together defined haplotype 16, which includes specific locus haplotypes at the three loci in and around CEN2 and at the large intron of SPBC 660.16. The widespread distribution of the strains with this haplotype suggests that the karyotype variant is long standing relative to the other rearrangements, and thus, haplotype 16 karyotype may define an incipient species. The other translocations were detected in single strains (NOTT 138, NOTT 140, NOTT 142, NOTT 143, and NOTT 145), all of which were derived by subcloning three African isolates. We analyzed all of these rearranged karyotypes first by pulsed field gel electrophoresis, then by filter transfer and hybridization (Figure 5A), and then for two of the karyotypes, NOTT 143 and NOTT 145, by comparative genomic hybridization (CGH) of gel-purified chromosomes to microarrays (Figure 5B), followed by PCR analysis and sequencing (File S1) across hypothetical breakpoints to produce the maps shown in Figure 5C. These demonstrate features held in common by the two rearrangements, first they are both translocations, and second, they both share a 2,227,883 bp pericentric inversion with respect to chromosome I of the laboratory strain. PCR showed that this inversion is present in all of the strains in the collection with the exception of DBVPG 2805, DBVPG 6610, DBVPG 4433, DBVPG 6279, DBVPG 6699, CRUK 972, and CRUK 975. These strains belong to haplotype 18 or the closely related haplotype 25. However, these strains have a widespread distribution, which suggested that the laboratory strain arrangement and possibly other rearrangements on a shared background are acting as barriers to fertile mating. Sequencing across the inversion and translocation breakpoints (File S1, and Figure S5) showed only microhomology in two of five sequences involved in the exchanges. Repetitive sequences were absent. This fact that the 2.23 Mb inversion was present in a minority of strains also suggested that the sequence arrangement in the laboratory strain was a derived trait, which we confirmed by comparison with the arrangement of the homologous sequences in *S. octosporus* and *S. cryophilus* (not shown). Pulsed field gel and filter hybridization analysis (Figure 5A) indicated that the rearrangements in NOTT 140 and 142 were also simple reciprocal translocations, and they were not characterized further. Filter hybridization and CGH microarray analysis showed that CBS 356 and NOTT 138 also contained translocations or transpositions, but in both cases, one of the two partner chromosomes was not detectably rearranged when analyzed by CGH on microarrays (not shown). The simplest explanation for these results is that one of the breakpoints is close to a telomere.

In addition to the analyses of the chromosomal rearrangements in CBS 356, NOTT 138, NOTT 140, and NOTT 142, we carried out comparative genomic hybridization (CGH) and microarray analysis CGH on unfractionated DNA of strains CRUK 972, NOTT 141, NOTT 143, NOTT 145, and CBS 2777 using CRUK 975 DNA as a competitor. This CGH analysis detected extensive regions of copy number variation (Table 5). Three of the eight regions that are amplified include genes that are involved in resistance to heavy metals, consistent with exposure to Bordeaux mixture (CuSO_4_/Ca(OH)_2_) used as a fungicide in vineyards.

**Table 5:** Regions of CRUK 972, CBS 356, CBS 2777, Nott 138, 141, 143, and 145 showing copy number variation

Strain	Chromosome	Rearrangement	Start Breakpoint	Finish Breakpoint	Size (kb)	Comments
NOTT 141	I	Duplication	3031645–3031834	3211591–3211839	180	
NOTT 143, NOTT 145	I	Duplication	3031645–3031834	3233014–3233274	201	
NOTT 138	I	Deletion	124442–124568	124866–125001	0.3	No annotated gene
NOTT 138	I	Duplication	5499761–5500081	5500384–5502054	2	Part of a galactosidase (mel1) gene
CRUK 972	II	Triplication	1598396–161093	1603682–1620842	19	Dgdh repeats
NOTT 141, NOTT 143, CBS 2777	II	Duplication	2036773–2037231	2041544–2041813	4	Includes ccc2-Menkes disease protein
NOTT 138, NOTT 141, NOTT 143, NOTT 145, CBS 2777	II	Deletion	356108–356292	359569–360000	3	SPBC1271.07E, SPBC1271.08E, mug96
NOTT 138	II	Loss of copy number	2114026–2115566	2116080–2116407	0.5	Mating type locus
CBS 356, NOTT 141, NOTT 143, NOTT 145, CBS2777	III	deletion	1493999–1494480	1499706–1500311	5	Deletion of pseudogene SPCC188.10c-1
NOTT 141, NOTT 143, NOTT 145 (4 copies)	III	Duplication	1893874–1894205	1926082–1926689	32	SPCC737.05-08, hmt1 (ABC transporter involved in response to Cd++), mug24, sly1
NOTT 141, NOTT 143, NOTT 145	III	Duplication	1174389–1174518	1180220–1180872	6	Includes SPCC4B3.01-1 putative 3-mercaptopyruvate sulfurtransferase
NOTT 141, NOTT 143	III	Duplication	381359–383288	383347–383709		SPCC1682.06
NOTT 138	III	Deletion	969398–970117	971646–971940	1	No annotated gene
CBS 2777, NOTT 138, NOTT 141, NOTT 143, NOTT 145	III	Deletion	1645637–1646096	1648511–1649231	2	Deletion of pseudogene SPCC663.07c-1
CBS 356, CBS 2777, NOTT 138, NOTT 141, NOTT 143	III	CNV polymorphic	2108532–2109334	2109565–2111026	1	wtf22; listed as a pseudogene

The indicated strains were analyzed by comparative genome hybridization using Agilent 4 × 44K ChiP-on-chip arrays, and the indicated regions of copy number variation were detected.

The strain CBS 2777 contained four chromosomes (Figure 5A). Preliminary pulsed field gel and filter hybridization analysis of intact chromosomal DNA demonstrated a complex pattern of sequence rearrangements. Detailed functional and structural analysis of this strain will be described elsewhere.

## Discussion

In many organisms, natural isolates have been a source of variation for the analysis of pathways and processes of basic biological interest. In the short term, the aim of such studies is to increase knowledge of the components of these pathways and processes, whereas in the long term, the aim is to understand the architecture of a trait or feature at all levels from DNA to phenotype in terms of the ecology of the respective organism (Mackay *et al.* 2009). We initiated the study of the variation of *S. pombe* because this organism is easy and cheap to manipulate and analyze and because much is known about the cell biology of *S. pombe*. We showed that the amount of phenotypic variation segregating in *S. pombe* is small in comparison to that seen, for example, in *S. cerevisiae* and that the existence of karyotypic rearrangements may limit access to some of it. We conclude that the variation that does exist (in size, for example) will need to be studied in a focused way. Our pulsed field gel-based approach is likely to have revealed the presence of only a subset of the translocations, inversions, and deletions segregating in the collection, and thus, a subset of strains, segregating a trait of particular interest, will need to be completely sequenced and characterized in terms of rearrangements. In light of the expense required to carry out such work, it would seem prudent to start with the most significant of the variable traits and characterize the strains segregating this variation first.

Cell size is a feature of the biology of *S. pombe* that has been intensively studied, is of general biological importance, and shows clear evidence of variation among the different isolates. Such variation is also observed in budding yeast (Nogami *et al.* 2007). It may be that this variation is adaptively neutral, and the correlation between the size of particular haplotype and the source from which it is isolated that is observed in haplotypes 3 and 4 of Brazilian origin has arisen by drift. Alternatively, the variation may be adaptively significant and related to the fact that the strains were isolated from different environments: caçhaca must (haplotype 3) or the frozen pulp of *Eugenia uniflora* (haplotye 4). A combination of experimental and ecological studies should discriminate between these alternative explanations and improve our understanding of this important aspect of cell physiology.

Although karyotypic diversity will make it necessary to adapt and apply the approaches used to exploit diversity in other model organisms to *S. pombe*, karyotype diversity is itself of interest. Thus, an understanding of the karyotype diversity is essential if we are to understand the nucleotide diversity because inversion and translocations will limit exchange between segments of DNA and strains. Rearranged chromosomes have been useful as both genetic reagents and in terms of their ability to cast new and often unexpected perspectives on fundamental problems. Thus the discovery of strain CBS 2777 with four chromosomes poses interesting questions about the mechanism of formation of additional centromeres and telomeres. The unexpectedly high levels of karyotypic diversity that characterize *S. pombe* worldwide also require explanation in population genetic and mechanistic terms. The simplest explanation, at the population genetic level, is that *S. pombe* exists in small, rarely out-crossing populations that favor the accumulation of weakly deleterious mutations, including karyotypic rearrangements. This explanation is consistent with both the pattern of linkage distribution around CEN2 and TER1 and with the observation that most of the rearrangements occur once in the collection or are confined to single haplotypes (Table S2). Note, however, that our perspective on out-crossing is based on only two regions of the genome and the patterns of LD within these regions will in turn be sensitive to whether they include any common rearrangements. Genome-wide estimates of LD will thus also require a knowledge of the karyotype diversity. A second, and not exclusive, adaptive explanation for the high levels of karyotypic diversity at the population genetic level is suggested by the isogenic rearrangements isolated by subcloning the African strains Y468, Y470, Y831, and Y832. The fact that the substrains are isogenic at the sequence level, vary karyotypically, and are derived from the same isolates raises the possibility that adaptive radiation may be occurring within in a limited geographic area and that the rearrangements are acting to isolate variants adapted to different, specific subniches. Although it may be possible to investigate such hypothetical microspeciation, a potential limitation of this line of work is that the traits under selection may be significant only for yeast ecology (*e.g.* sugar utilization) and not of general interest as regards eukaryotic cell biology. However, it seems worthwhile to study this aspect of the biology of *S. pombe* because speciation is a problem of general interest that remains incompletely understood, and *S. pombe* offers the possibility of rigorous analysis and understanding. In this respect, our population level approach extends the comparative genomics analysis of four different *Schizosaccharomyces* species by Rhind *et al.* (2011). Deeper understanding at both levels should help cast light on the mode and tempo of speciation in *Schizosaccharomyces spp*. and, in particular, on the relative significance of karyotypic and gene incompatibility as isolating mechanisms in this genus (Greig 2009).

It is clear from our analysis that our understanding of the origins and spread of *S. pombe* would be improved not only by more sequence data and data about the rearrangements segregating in the population but also by the collection and analysis of many more strains from around the world. *S. pombe* will grow at high concentrations of glucose and at low pH. An enrichment medium for *S. pombe* has been described (Florenzano *et al.* 1977). Thus, it may be possible to collect many more strains, although the ease with which this might be done is not yet clear. In particular, more strains from Africa and South and East Asia will be necessary to establish the geographic origins and phylogeny of the species. The success of the Brazilian collection expeditions suggests that it should be possible to isolate such strains. This work should also help define the range of environments that harbor *S. pombe*, provide additional karyotypic variants, and perhaps identify convenient locations for field work.

## Supplementary Material

Supporting Information
