# Supplementary material for: A Geographically Diverse Collection of Schizosaccharomyces pombe Isolates Shows Limited Phenotypic Variation but Extensive Karyotypic Diversity
Source: G3 (Bethesda). 2011 Dec 1;1(7):615–26. doi: 10.1534/g3.111.001123 (PMC3276172; doi:10.1534/g3.111.001123)
Supplement: Supporting Information [file supp_1_7_615__index.html]

Supporting Information 

# A Geographically Diverse Collection of *Schizosaccharomyces pombe* Isolates Shows Limited Phenotypic Variation but Extensive Karyotypic Diversity

## Supporting Information for Brown *et al.*, 2011

**Files in this Data Supplement:**

- Supporting Information - Figures S1-S10, File S1, and Tables S1-S5 (PDF, 2.1 MB)
- Figure S1 - *S. pombe* proliferates more slowly than *S. cerevisiae* in a wide range of environments (PDF, 108 KB)
- Figure S2 - Natural trait variation in *S. pombe* is defined by geographic boundaries (PDF, 72 KB)
- Figure S3 - The *S. pombe* karyotype extreme, CBS2777, shows abnormal proliferation patterns (PDF, 84 KB)
- Figure S4 - A karyotype re-arrangement shared between the *S. pombe* type strain CBS 356 and five other strains (PDF, 440 KB)
- Figure S5 - Identification of the sites of re-arrangements in NOTT 143 and NOTT 145 (PDF, 164 KB)
- Figure S6 - SNPs and haplotype structure in the central core of *S. pombe* chromosome II (PDF, 56 KB)
- Figure S7 - SNPs flanking the centromere and inormative SNPs used in four gamete test (PDF, 68 KB)
- Figure S8 - SNPs identified in the TER1 gene and in adjacent sequences (PDF, 68 KB)
- Figure S9 - Network of haplotypes at the TER gene (PDF, 64 KB)
- Figure S10 - SNPs identified in intron 3 of the SPBC660.16 gene (PDF, 64 KB)
- File S1 - Supporting Text (PDF, 292 KB)
- Table S1 - Strains used in this study (PDF, 104 KB)
- Table S2 - Assorting 84 strains of *S. pombe* into 40 groups with shared haplotypes (PDF, 292 KB)
- Table S3 - Primers used to make probes used in hybridization analysis (PDF, 40 KB)
- Table S4 - Primers used to amplify DNA for diversity analys (PDF, 40 KB)
- Table S5 - The proliferative lag (time to initiate proliferation), proliferative rate (population doubling time) and proliferative efficiency of natural *S. pombe* isolates over 42 environments (.txt, 120 KB)
