# Supplementary material for: A Geographically Diverse Collection of Schizosaccharomyces pombe Isolates Shows Limited Phenotypic Variation but Extensive Karyotypic Diversity
Source: G3 (Bethesda). 2011 Dec 1;1(7):615–26. doi: 10.1534/g3.111.001123 (PMC3276172; doi:10.1534/g3.111.001123)
Supplement: Supporting Information [file supp_1.7.615_001123SI.pdf]

File S1

Supporting Text

1 Strain collection

We used the web, the scientific literature and personal contacts to identify strain collections holding isolates of fission yeast. This led to a collection which includes 84 isolates of *S. pombe* (supplementary data Table 1). Each isolate in our collection retains its original designation. All but one of the strains were haploid as judged by DNA staining. The one strain; NCYC 2355, that was diploid yielded isogenic haploid clones upon re-streaking and we worked with one of these and for this reason it is marked with an asterisk. DBVPG 4433 is listed as *Zygosaccharomyces* but was found to contain fission yeast upon colony purification by GL.

2 Structural Analysis of re-arranged chromosomes

2.1 Analysis of Nott 143, Nott 145 and other strains containing re-arrangements involving sequences on chromosomes I and II of the laboratory strain.

As shown in the main text chromosome 1 in NOTT 143 was a derivative of chromosome II and chromosome 2 was the reciprocal chromosome I derivative. Micro-array analysis showed that the translocated sequences on NOTT 143 chromosome 1 were derived from two discontinuous regions of laboratory strain chromosome I; from the telomere of the left arm of chromosomes I to sequences between 2,683,519 and 2,683,704 and from sequences between 4,642,534 and 4,643,255 to sequences between 4,911,470 and 4,911,584. This pattern suggested a compound re-arrangement in which sequences from a variant of chromosome I containing a large pericentric inversion were translocated onto chromosome II. We used the micro-array data to design primers flanking hypothetical breakpoints and confirmed this compound re-arrangement by PCR (supplementary data; figure 2) and determined the identity of the breakpoint sequences and in particular the existence of a 2,227, 883 bp inversion across the centromere of chromosome I of the laboratory strain with respect to NOTT 143. The breakpoint on NOTT 143 chromosome 2 disrupted the gene SPAC30C2.07-1 and generated a truncated fusion protein of unknown functional significance (see below). We wanted to determine whether the inversion was ancestral or derived and we therefore analysed the entire collection for the presence of the inversion by PCR. All of the strains in the collection with the exception of DBVPG2805, NCYC 3092, DBVPG6610, DBVPG 4433, DBVPG 6279, DBVPG 6699, CRUK 972, CRUK 975 contained the sequence organization seen in NOTT 143 with respect to the inversion breakpoints. This suggested that the sequence arrangement in the laboratory strain was a derived trait and we confirmed this by comparison with the arrangement of the relevant sequences in *S. octosporus* and *S. cryophilus* (not shown). In addition to the inversion NOTT 145 was re-arranged by a translocation of between II R and I L giving rise to a derived chromosome 1 that was larger than the laboratory strain chromosome I and a derived chromosome 3 that at 3.2Mb was

slightly smaller than the rDNA containing chromosome. We were able to identify the breakpoint on NOTT 145 chromosome 3 but not on chromosome 1. We used two sets of primers suggesting that the failure to identify the chromosome 1 breakpoint may be due non-reciprocity. In NOTT 145 the chromosome 3 breakpoint was associated with a C-terminal truncation of SPBC24C6.09C-1. Otherwise all breakpoints were reciprocal with respect to the laboratory strain sequence with the exception of the chromosome I inversion, which involved the loss of a single residue (below). Filter hybridization analysis indicated that the re-arrangements in NOTT 140 and 142 were simple reciprocal translocations and they were not characterized further. Chromosome 1 in CBS 356 contained all of the chromosome II sequences used in the hybridization analysis as well as both of the distal sequences from chromosome I. Chromosome 2 of CBS 356 corresponds to chromosome III of the laboratory strain and chromosome 3 to a deleted derivative of chromosome I. We have not carried out any further analysis of this strain. Micro-array analysis of the chromosomes of NOTT 138 show that a 1.7Mb block of sequence from chromosome II has been transposed onto chromosome I to generate a derivative of chromosomes I and II corresponding to chromosome 1 and 3 of NOTT 138. Chromosome 1 of NOTT 140 contains sequences from the centromere proximal regions of chromosome I, distal I R and distal II L while NOTT 140 chromosome 2 contains sequences from the proximal regions of chromosome II, distal II R. and distal I L This result is suggests a exchange of material that is normally present on the distal left arms of chromosomes I and II of the laboratory strain giving rise to a derived II that is about 600kb smaller than chromosome II of the laboratory strain and a derived I that is larger than laboratory strain chromosome I by a similar amount. NOTT 140 contains the ancestral pericentric inversion of chromosome I and so the I R sequences are now on the left arm of the chromosome 1 in this strain.

2.2 Sequences of breakpoints in strains NOTT 143 and NOTT 145

NOTT 143: chromosome 1 breakpoint

```
I: 4642748
ccaaacatcagatcagtaagtggagcatttgccgagtccctaagcacactatctcctttctg/
I: 4642687
II: 1445844
/tatatttattgctactatcgcttttagaacatcatgttattaataatccgtaactagaccgtttaat
II: 1445910
```

NOTT 143: chromosome 2 breakpoint

```
II: 1445778
cctgtgcatgatttactttttaaattcagggttaattaattactttccatcatttcctcctctccat/
II: 1445844 / I: 4642686
/accatccacatgattagagtcaaacaaaacagtttttttcggtctccgatgtncttgaancgagga
caaga
I: 4642617
```

Region of homology highlighted

Disruption of SPAC30C2.07-1 (842 amino acids in CRUK 972) by the introduction of premature stop codon

I-4,642,495

atgcttcatttttatttcattcaggatcttcatcaaatagaaac**tcatcgccgaaagaatcctatgagcttctt**
M L H F L F H S G S S S N R N **S S P K E S Y E L L**
catggtttggacaaacaatatcaaagtactaaggatgtaacgtttcgtcttgtcctcgttcaagacatcggagac
H G L D K Q Y Q S T K D V T F R L V L V Q D I G D

I: 4642686 / II: 1445844

cgaaaaaaaactgttttgtttgactctaatcatgtggatggt/atggagaggaggaaatga 210
R K K T V L F D S N H V D G M E R R K * 69 amino acids

NOTT 145: chromosome 3 breakpoint

II:2336713
ctaaaattcgccattcaaatacttgggacatcctttacccttaggggtacgaaggataatcatgggcc
atctgggtttgatttcttctctgttaacacgagcgcgatgttggatgtcatgaatgcgttcaacggcc
caatc**cat**a/
II: 2336858 / I: 823412
/taattacgtggagctaccttaaattcaattaggtagtttaaaatctatcccagaaagctaacttact
gagaagtcctcgctaaaccatattagcgcatgtagcatttaatgaattattcgtgtggatattagata
actgatttaggttgattttgtagcttatacaattnccttttcttttggtataataaagccaaaat
cgtaaacaaagatgaattggaataaacattctttcacaatacttccacgagttcancatcgccatgtg
cgaacctcgttatatctttgaccntcgaaattcttgnagtccctgtcc
I: 823099
Potential new ATG indicated giving a C terminal product of SPBC24C6.09C-1
1596 nt; 532 amino acids truncation of CRUK 972 protein

Common chromosome I inversion (2,227,883 bp) breakpoints:

Left breakpoint

I: 2683567
tgttttcaaaatataaccacgggtaagtaatacagttggcatacaacatttgggtaacc
toccata / I:2683632 / I: 4911514
gcaagctagatctagattaatgctataggcatacnaaac
I: 4911476

Right breakpoint: underlined residue lost

I: 2684324
Agatcttagcaaggtttcggatatgtcatgggtaaagttcgatngctttgtgcatctccgttagcagc
tgcatcaagcgagtatccaacgatgatcatctcgattcgttttgtgtgttcatgaaacccacgccttg
cgtatgaagttgcccactttgtatgacaatgagataatcttacactgcaataaggccatatacccgta
attggtgggnacnaccactcacattccaaagtccaactcaatagtgaagaaattcatcattggcaaca
agtcatcaacacttcgttgctgttggacccttctaccaaacgttctttattgacaactttgaacggct
tcggctgcnttcccgagccctgtaacgcctatctttcatgtggctcggaatgtaaaccagaaactcag
acaaataccgctgttgatcgcgctgatgatatttgtttatcgaggctgtgttcatttttcttagtcat
tttagcgtaacgaactgacaccgaaaaatatcaagcaatcaagaagatccatctgtccaagcaccaac
cacatctttgctcgataatcaaacttggattaccgatagttaaaggtgaaggatcggttttgctggtt
tcattcgattttccaaacatcgtcgattcagtagactggggttgagtcagtatccaagccattcttgc
gctcaattgataat/**t** / I: 2683632 / I: 4911515
gtcatataacgaccagatttactcttgaccgacttcatagcagcataattagcntntccnnngncata
aaagaaaaaatttgctatctccactctcacaaggttagcgaaggtttgtgcatttgacagcaggttg
gttaaattcgttaagcttccaagagatataaatatagtggctcgaatttgacttatccagtcagtcat
tcattgctgaagttgttgggttggccag I: 4911745

3 Quantifying natural trait variation in *S. pombe*;

Strains were stored in 20% glycerol at -80° C. Strains were subjected to high throughput phenotyping by micro-cultivation (n=2) in an array of environments essentially as described (PMID: 12489126, PMID:18721464). First, strains were inoculated in 350 µl of YES medium (5% yeast extract, 3% glucose, 225mg/L histidine, 225mg/L adenine, 225mg/L leucine and 225mg/L uracil) and incubated in two serial rounds of pre-cultivation for 48h-72 h at 30C°. For experimental runs, strains were inoculated to an OD of 0.05 - 0.1 in 350 µl of YES medium (3% glucose was replaced by 3% of alternative carbon sources where indicated) in the relevant environments and micro-cultivated for 48 or 72h in a Bioscreen analyzer C (Growth curve Oy, Finland). Environments were: 4-nitroquinolone (0.3µg/ml), $AlCl_3$ (0.2mg/ml), arabinose (3%; carbon source), Arsenite (0.2, 2mM) $BaCl_2$ (25mM), Caffeine (1.5mg/ml), $CdCl_2$ (25µM), Cisplatin (100µg/ml), $CoCl_2$ (0.25mM), CsCl (12.5mg/ml), $CuCl_2$ (5mM), Cycloheximide (0.05 µg/ml), Diamide (1.4mM), EMS (0.3%), Ethanol (3%; carbon source), Galactose (3%; carbon source), Glycerol (3%, carbon source), Heat (37, 40°C), $HqCl_2$ (7.5 15µM), Hydroxyurea (8mg/ml), KCl (1.45 M), LiCl (225mM), Maltose (3%; carbon source), Melibiose (3%; carbon source), $MgCl_2$ (1.25mM), MMS (0.0015%), $MnCl_2$ (0.1mM), NaCl (0.05, 0.1, 0.4 M), $NiCl_2$ (0.75mM), Paraquat (200µg/ml), $Pb(NO_3)_2$ 0.4mM, Selenite (0.1mM), $SrCl_2$ (25mg/ml), Sucrose (3%; carbon source), Tellurite (30.5mM) and Tunicamycin (1µg/ml). All incubations were at 30°C (±0.1°C), except for when heat stress was applied (see above). Pre-heating time was set to ten minutes. Plates were subjected to shaking at highest shaking intensity with 60s of shaking every other minute. Optical density (OD) was recorded using a wide band (450-580 nm) filter every 20 minutes. Strains were run in duplicates on separate plates and normalized to four replicates of the *S. cerevisiae* universal type strain BY4741 in randomised (once) positions on each plate. The plate layout was kept unchanged between duplicates and runs. Runs affected by systematic technical problems were discarded as were isolates severely affected by flocculation (four isolates).

Data analysis: Optical density measurements (OD) were calibrated in the following way:

i) Background correction was achieved by subtracting the SD medium background of 0.067.

ii) Non-linearity of optical density and population density at higher population densities was compensated for by calibration of each OD measurement as: calibrated OD = OD + 0.8324*OD^3 (PMID: 14676322, PMID: 12489126)

iii) Noise reduction was achieved by a moving average smoothening: each OD measurement, $X_{i,smoothened} = (X_{i-1}+X_i+X_{i+1})/3$.

iv) Confounding effects from stationary phase proliferation curve collapses was compensated for by removing all negative slopes (if $X_{i+1} < X_i$ then set $X_{i+1} = X_i$).

From calibrated proliferation curves, each fitness variable was calculated as:

Proliferation lag: Growth measurements were LN transformed, slopes were calculated between every pair of measurements spaced 140min apart, intercepts between each calculated slope and the extended horizontal line given by the start OD (average of initial five measurements) were calculated and the mean of the two highest intercepts was taken as the length of the lag phase. Proliferation lags longer than 48h were set to 48h.

Proliferation rate: Growth measurements were LN transformed. Slopes were calculated between every pair of measurements spaced 40min apart along the curve (no slopes were calculated from the eight initial time points to filter for digitalisation effects), the top two slopes were discarded to exclude possible artefacts and a mean was calculated from the third to the eight highest slopes. Population doubling time was calculated as LN(2) divided by the mean. Population doubling times longer than 48h were set to 48h.

Proliferation efficiency: The difference between end OD (last measurement) and initial OD (average of initial five measurements) was calculated and taken at face value as a measure of total change in population density. No proliferation efficiency was calculated from growth curves for which no stationary phase was reached, defined as curves with a coefficient of variation over the last five measurements >2%. Proliferation efficiency lower than 0.05 OD units were set to 0.05 OD units.

The calculated fitness variables were transferred onto log scale (LN_2). To normalize fitness variables across plates, runs and instruments, each fitness variable was related to the corresponding measure from four reference strains (BY4741) included on the same plate. Relative fitness measures for each strain and trait, LSC_{ij}, were calculated as:

$$\mathrm{LSC}_{ij} = \frac{\sum_{r=1}^{2}\left[\left[\frac{1}{10}\sum_{k=1}^{10}\log\left(wt_{kj}^{r}\right)\right]-\log\left(x_{ij}^{r}\right)\right]}{2}$$

where wt_{kj} is the fitness variable of the k:th measurement of the wildtype for trait *j*, x_{ij} is the measure of strain *i* for trait *j* and *r* indicates the run. The LSC for proliferation efficiency was inverted in order to maintain directionality between fitness variables.

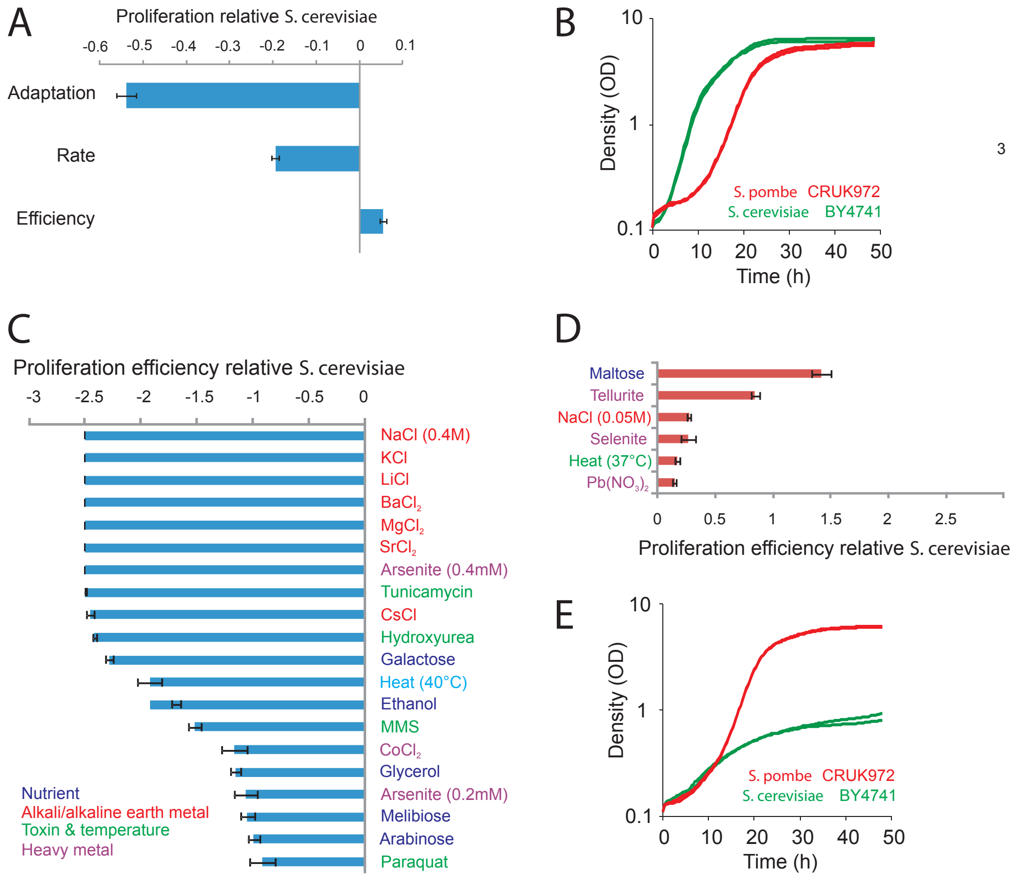

Figure S1 *S. pombe* proliferates more slowly than *S. cerevisiae* in a wide range of environments. A-B) *S. pombe* shows a much longer mitotic lag phase and a slower rate of mitotic proliferation than the *S. cerevisiae* universal type strain BY4741 in basal conditions (Synthethic Defined medium). A) The proliferative lag, rate and efficiency of *S. pombe* natural isolates in basal conditions were calculated and put in relation to the corresponding measures of BY4741 (Log2 [isolate/BY4741]). Mean and Standard Error of the Mean are displayed. B) Proliferation of the *S. pombe* type strain 972h- (N=2) in basal conditions. BY4741 (N=2) is shown as reference. C) Top 20 environments in which the *S. pombe* proliferative efficiency is inferior to that of the *S. cerevisiae* universal type strain BY4741. A relative proliferative efficiency, (Log2 [isolate/BY4741]), was calculated for all *S. pombe* natural isolates. Means and Standard Error of the Means for each environment are displayed. D) Top 5 environments in which the *S. pombe* proliferative efficiency is superior to that of the *S. cerevisiae* universal type strain BY4741. A relative proliferative efficiency, (Log2 [isolate/BY4741]), was calculated for all *S. pombe* natural isolates. Mean and Standard Error of the Mean for each environment are displayed. E) Proliferation of the *S. pombe* type strain 972h- (N=2) using maltose as sole carbon source. BY4741 (N=2) is shown as reference.

FS2; Brown et al

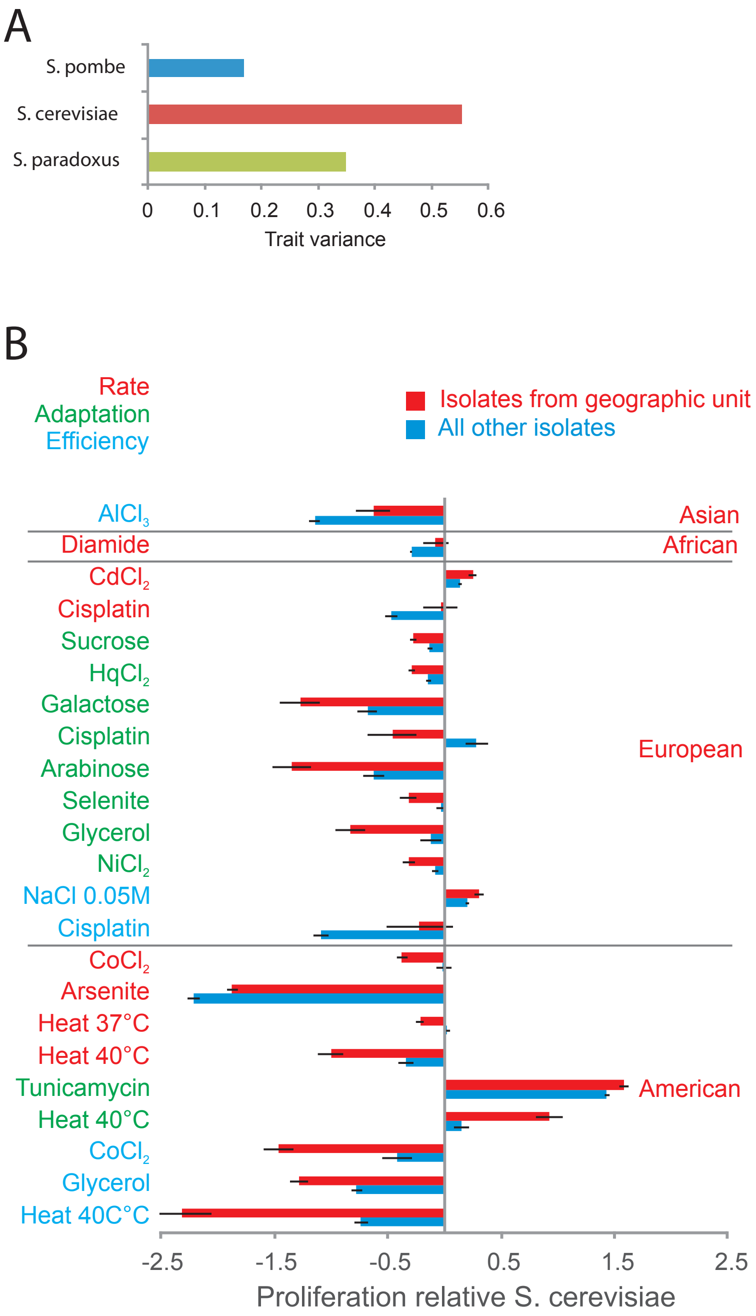

Figure S2 Natural trait variation in *S. pombe* is defined by geographic boundaries. A) Trait variation in natural isolates *S. pombe* is substantially smaller than trait variation in *S. cerevisiae* and *S. paradoxus*. Trait variance within *S. pombe* was calculated for each trait separately and a mean over all traits was formed, excluding environments in which more than 50% of S. pombe isolates failed to proliferate. Trait variance in *S. cerevisiae* and *S. paradoxus* was obtained from (ref) in which the mitotic proliferation in a similar, but wider array of environments was quantified using an identical experimental set-up. Error bars represent standard errors of the mean. B) Traits for which *S. pombe* isolates from one geographic area differ significantly (Students ttest, FDR=5%) from all other *S. pombe* isolates. Means and Standard Error of the Means are displayed.

FS3; Brown et al

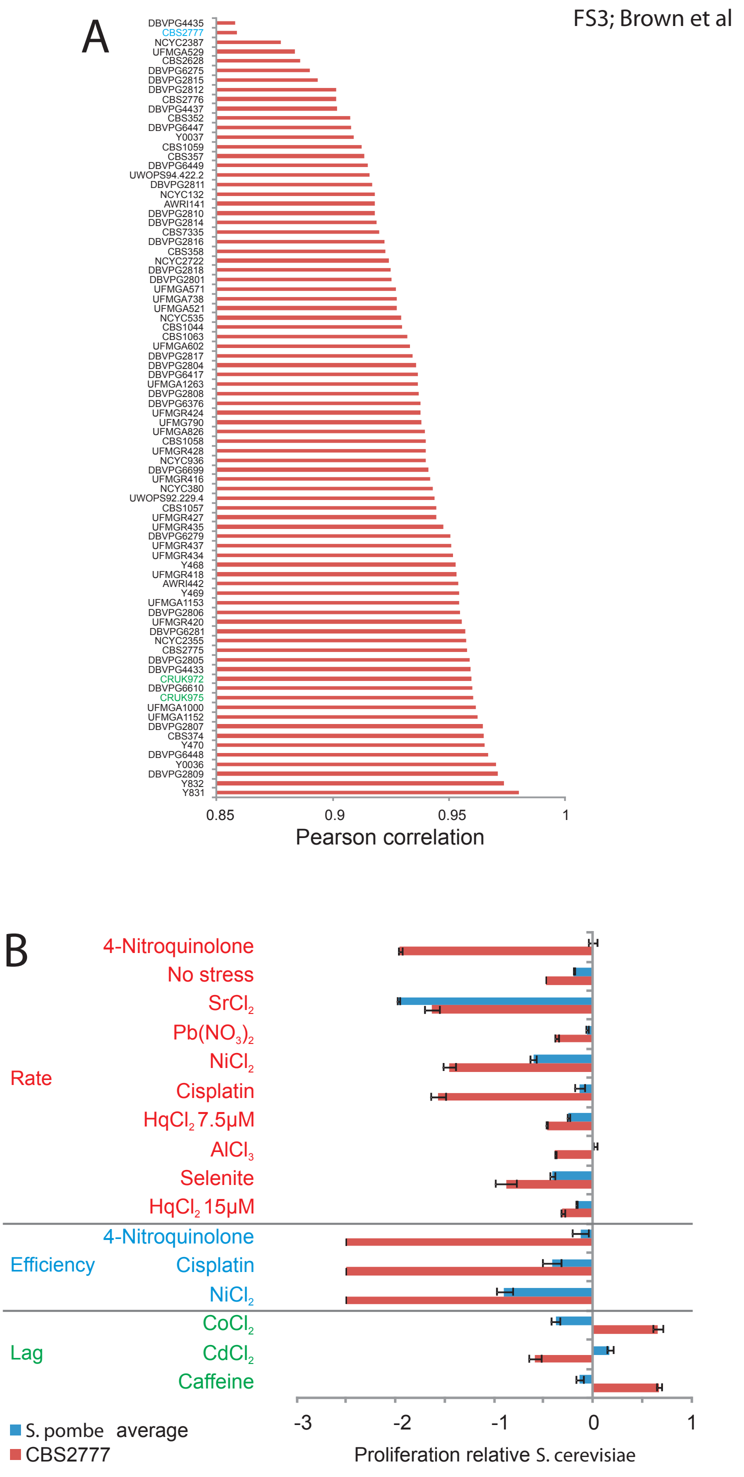

Figure S3 The *S. pombe* karyotype extreme, CBS2777, shows abnormal proliferation patterns. A) *S. pombe* mean trait profile was calculated over all traits and the similarity (Pearson correlation) between the mean trait profile and the trait profile of each individual *S. pombe* isolate was calculated. Isolates were ranked according to degree of similarity. The *S. pombe* karyotype extreme CBS2777 (blue) and the universal reference strains 972h- and 975h+ (green) are indicated with color. B) Traits for which the *S. pombe* karyotype extreme CBS2777 (N=2) differ significantly (Students ttest, FDR=5%) from all other *S. pombe* isolates. Means and Standard Error of the Means are displayed.

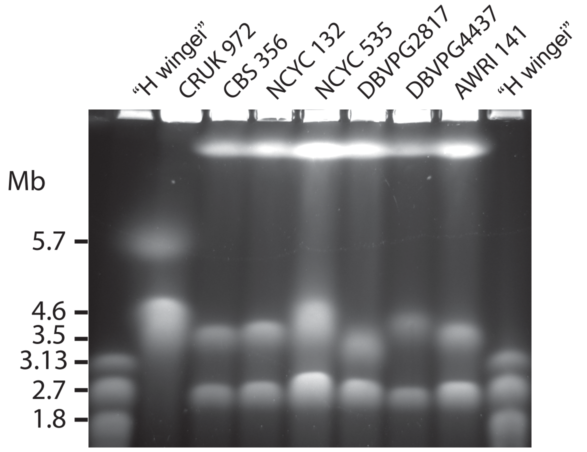

Figure S4 A karyotype re-arrangement shared between the *S. pombe* type strain CBS 356 and five other strains. DNA was extracted from the *S. pombe* laboratory strain CRUK 972 , the type strain, CBS 356 and five other strains, NCYC 132, NCYC535, DBVPG2817, DBVPG4437 and AWRI 141 and analysed by pulsed field gel electrophoresis and ethidium bromide staining. The markers used in the flanking tracks were obtained from Bio-Rad and marketed as chromosomal DNA derived from *Hansenula wingei*. However we have prepared chromosomal DNA from the *Hansenula wingei* type strain and do not observe the same pattern, thus the label is in parentheses. The variability of the chromosome 2 is characteristic of the presence of the rDNA.

FS5; identification of the sites of
re-arrangement in NOTT13 and NOTT145

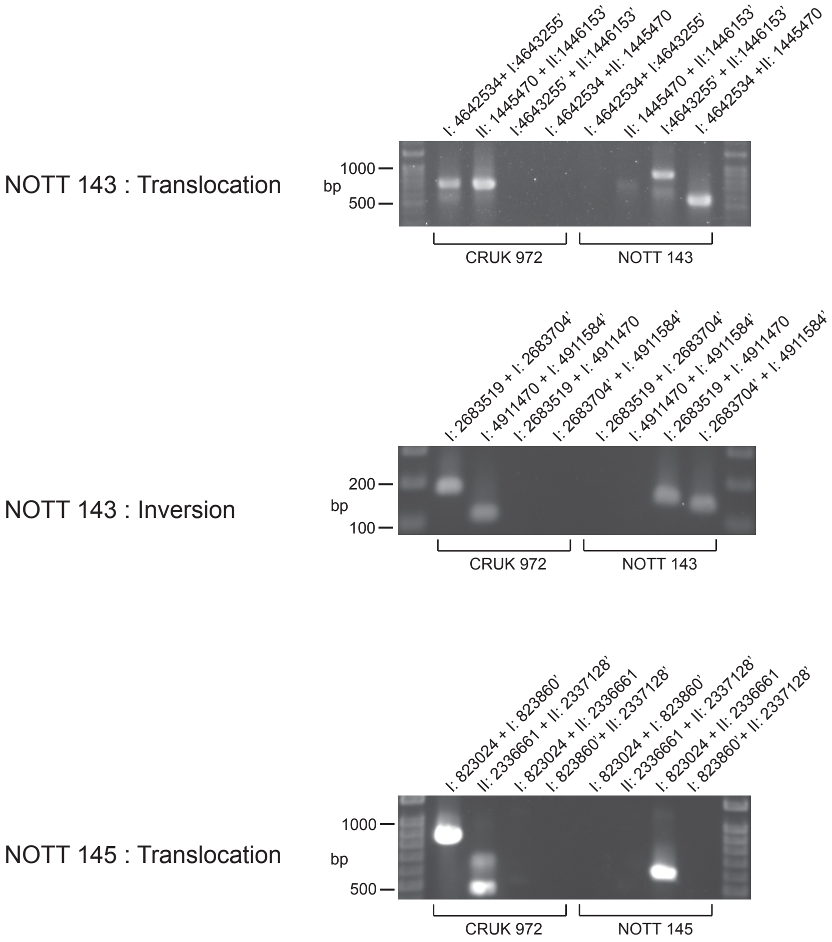

Figure S**5** Identification of the sites of re-arrangements in NOTT 143 and NOTT 145. DNA was analysed by PCR using the indicated primers for the three re-arrangements identified in the CGH analysis of strains NOTT 143 and NOTT 145. CRUK 972 DNA was used as the control. The numbers refer to the positions of the 5' residue of the respective primers on the assembly of the laboratory strain. The ' indicates that the primer corresponds to the complementary strand of the laboratory strain assembly.

Central Core Centromere II

A

Position

```
Haplotype:   1621091 1621094 1621110 1621127 1621146 1621261 1621265 1621288 1621290 1621324 1621340 1621354 1621381 1621420
abundance    1621477 1621497 1621522 1621523 1621606 1621615 1621628 1621636 1621660 1621694 1621748 1621763 1621795 1621799

 1:26        T C A A G TCGTA C - T - T G A T C GC C C A A T T - C C C
 3:1         . . . . . ..... T - . - . . . . . .. . . . . . . - . . .
 2:1         . . . . . ..... . T . - . . . . . .. . . . . . . - . . .
 5:1         . . G . . ..... . - . - . . . . . .. . . . . . . - . . .
 8:1         . . . . . ..... . - . - . . . . . .. . . . . . . - . . T
 4:1         . . . . . ----- . - . - . . . . . .. . . . . . . - . . .
 7:1         . . . . . ----- . - . - . . . C . .. . . . . . . - . . .
10:1         . . . T . ..... . - . - . . . . . .. . . . . . . - . . .
 6:6         A T . . A ..... . - C A G A G . T -- T T T T G C A G G .
 9:1         A T . . A ..... . - C A G A G . T -- T C T T G C A G G .
```

B

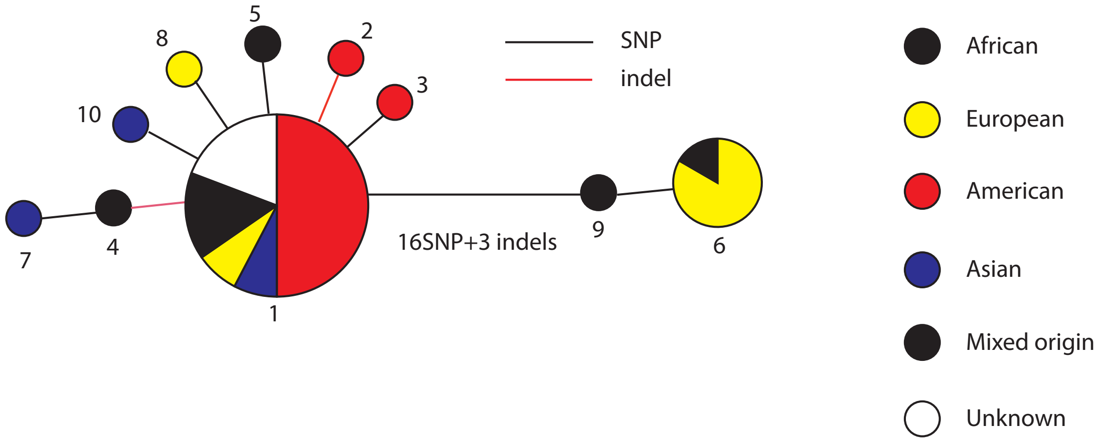

Figure S6 SNPs and haplotype structure in the central core of *S. pombe* chromosome II. A, The sequences shown in the text were analysed using the FABbox suite to identify informative residues B. The sequences shown in A were analysed for their relatedness using Network (Version 4.6.0.0, Fluxus Technology http://www.fluxus-engineering.com/sharenet.htm) to produce the network of haplotype relationships based on a median joining network using default parameters. The numbers adjacent to the nodes in the network refer to the specific central core haplotypes listed in A. The areas of the individual nodes are proportional to the numbers of compound haplotypes that contain the particular central core haplotype. The colors refer to the geographical origins of the respective haplotypes as indicated. Also detailed are the numbers and types of mutation separating the respective nodes.

CEN2 L; 659bp

Haplotype: abundance	**1572349**	1572524	1572778	1572987
1:17	G	C	A	C
2:19	A	.	.	.
3:1	A	T	.	.
4:2	A	.	.	G
5:1	A	.	G	.

CEN2 R; 719bp

Haplotype: abundance	1658038	**1658392**	1658425	**1658455**	1658566
1:23	T	T	A	G	A
2:3	.	.	C	.	.
3:4	.	C	.	.	.
4:1	C	.	.	.	.
5:1	.	C	.	.	T
6:7	.	C	.	A	.
7:1	C	C	.	.	.

CEN2 compound haplotypes

Haplotype: abundance	**1572349**	**1621091**	**1658392**	**1658455**
1:17	G	T	T	G
2:6	A	T	C	G
3:10	A	T	T	G
4:7	A	A	C	A

Figure S7 SNPs flanking the centromere and inormative SNPs used in four gamete test. The figure illustrates the positions of the SNPs identified in the sequences flanking the centromere of chromosome II. Informative SNPs are shown in bold. The four compound haplotypes composed of informative SNPs are indicated below.

Haplotype: abundance	3084500	3084506	3084568	3084571	3084743	**3084762**	3084771	3084778	3084779	3084929	3084934	3084944	3084970	3085000	3085001	3085013	3085016	3085085	3085203	3085232	3085238	3085242	3085352	3085405	3085432	3085436	3085448	3085464	3085507	3085520	3085546	3085574	3085611	3085720	3085776	3085966	**3086123**	3086146
1:1	T	G	T	A	T	A	G	A	A	G	C	G	T	A	C	T	A	A	C	A	T	T	T	T	C	T	G	A	A	A	C	A	T	G	A	T	T	T
5:1	.	.	.	.	.	.	.	.	.	.	.	.	.	.	.	.	.	.	.	.	–	.	.	.	.	.	.	.	.	.	.	.	.	.	.	.	.	.
2:1	.	A	.	.	.	.	.	.	.	.	.	A	.	.	.	A	.	.	.	.	–	.	.	.	.	.	.	.	.	.	.	.	.	.	.	.	.	.
13:2	.	A	.	.	.	.	.	.	.	.	.	A	.	.	.	.	.	.	.	.	–	.	.	.	.	.	.	.	.	T	.	.	.	.	.	.	.	.
15:1	.	A	.	.	.	.	C	.	.	.	.	A	.	–	T	.	.	.	.	.	–	.	.	.	.	.	.	.	.	T	.	.	.	.	.	.	.	.
3:5	A	A	.	.	.	.	.	.	.	.	.	A	.	.	.	.	.	.	.	.	–	.	.	.	.	.	.	.	.	T	.	.	.	.	.	.	A	.
7:3	A	A	.	.	.	.	.	.	.	C	.	A	.	.	.	.	.	.	.	.	–	.	.	.	.	.	.	G	.	T	.	.	.	.	.	.	A	.
14:1	A	A	.	.	.	.	.	–	–	.	.	A	.	.	.	.	.	.	.	T	–	.	.	.	.	.	.	.	.	T	.	.	.	.	.	.	A	.
11:1	A	A	.	.	.	G	.	.	.	.	.	A	.	.	.	.	.	.	.	.	–	.	.	.	.	.	.	.	.	T	.	.	.	.	.	.	A	.
10:1	A	A	.	G	G	.	.	.	.	.	T	A	.	.	.	.	.	.	.	.	–	.	C	.	.	.	A	.	.	T	T	.	C	.	G	C	.	.
9:1	A	A	C	.	G	.	.	.	.	.	.	A	.	.	.	.	.	.	.	.	–	–	C	.	.	.	A	.	.	T	T	.	C	.	G	C	.	.
4:2	A	A	.	.	.	G	.	.	.	.	.	A	.	.	.	.	T	C	.	.	–	–	.	A	G	.	.	.	T	T	T	G	.	A	.	C	.	C
6:17	A	A	.	.	.	G	.	.	.	.	.	A	.	.	.	.	T	C	.	.	–	–	.	.	G	.	.	.	T	T	T	G	.	A	.	C	.	C
8:1	A	A	.	.	.	G	.	.	.	.	.	A	.	.	.	.	T	C	.	.	–	–	.	.	G	–	.	.	T	T	T	G	.	A	.	C	.	C
12:1	A	A	.	.	.	G	.	.	.	.	.	A	C	.	.	.	T	C	.	.	–	–	.	.	G	.	.	.	T	T	T	G	.	A	.	C	.	C
16:1	A	A	.	.	.	G	.	.	.	.	.	A	.	.	.	.	T	C	A	.	–	–	.	.	G	.	.	.	T	T	T	G	.	A	.	C	.	C

TER1+30Y

	3113994	3114036	3114236	**3114239**	3114280	3114344	**3114431**	3114480
1:21	A	A	T	A	T	–	T	G
2:10	.	.	.	G	G	A	A	.
3:3	G	.	.	.	.	–	.	.
4:2	.	.	.	.	.	A	A	.
5:3	.	G	A	G	G	A	A	.
6:1	.	.	.	.	.	A	A	A

TER1+100Y

	3194616	3194679	3194724	**3194949**	3195027
1:24	T	C	T	C	G
2:12	.	T	.	G	A
3:1	.	.	C	G	.
4:2	.	.	.	G	A
5:1	C	.	.	.	.

Figure S8 SNPs identified in the TER1 gene and in adjacent sequences. The figure illustrates the positions of the SNPs identified in the TER1 gene and the two adjacent sequences studied. Informative SNPs used in the linkage disequilibrium (Table 4) measurements are shown in bold.

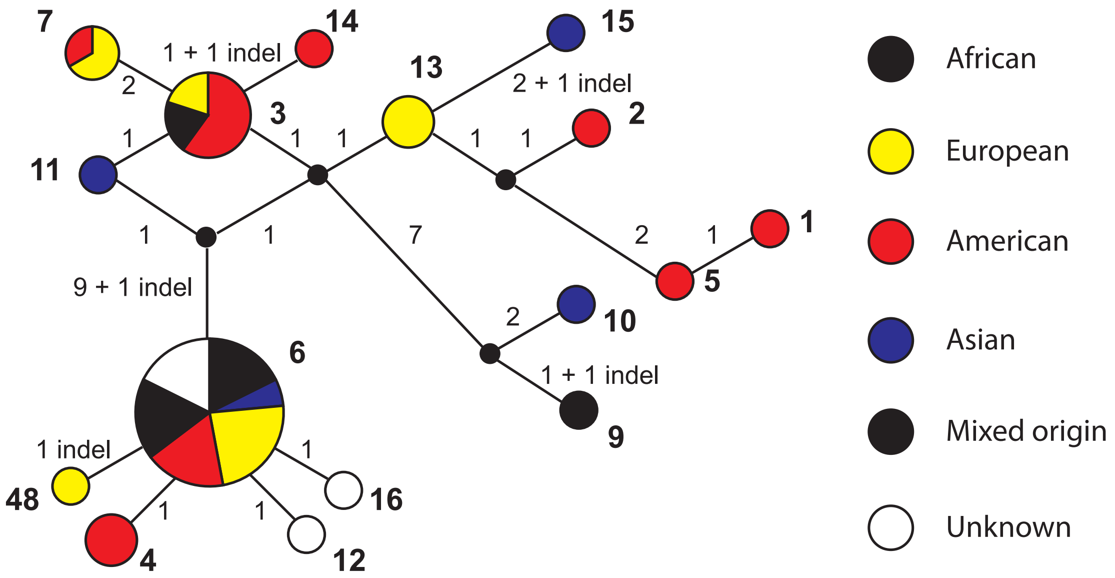

Figure S9 Network of haplotypes at the TER gene. The sequences shown in figure S8 were analysed as in Figure S6. The numbers in bold refer to the haplotypes indicated in S8. The areas of the individual nodes are proportional to the numbers of compound haplotypes that contain the particular haplotype. The numbers between adjacent to the branches refer to the number of SNPs or indels separating the respective nodes. The colors refer to the geographical origins of the respective haplotypes as indicated.

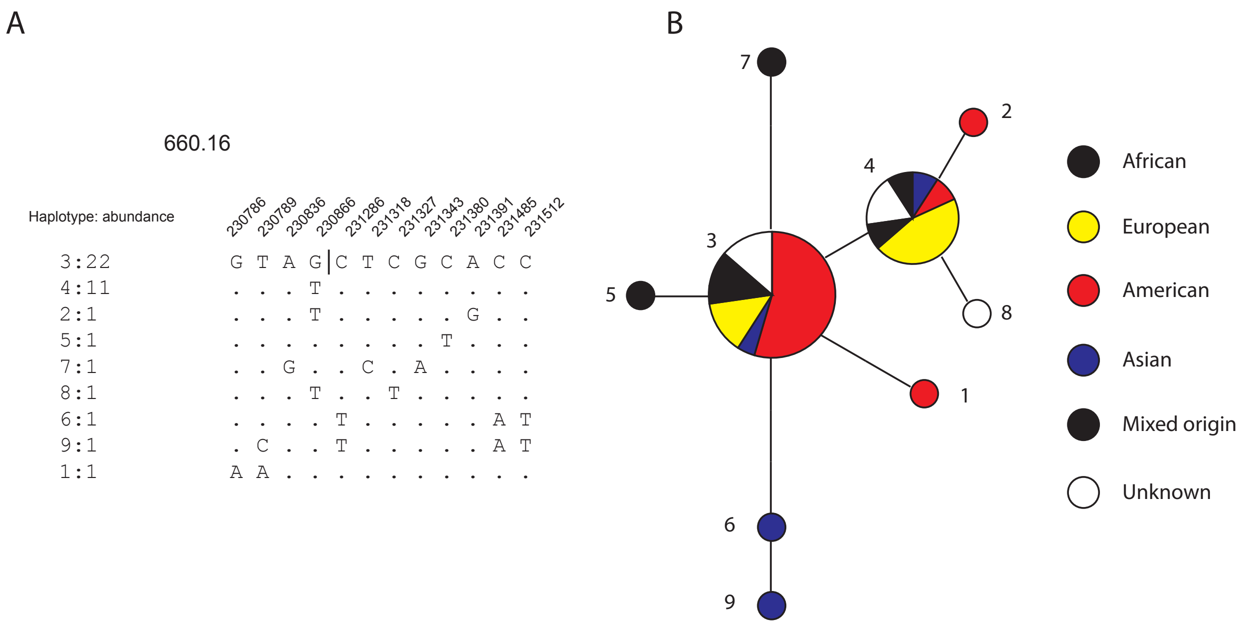

Figure S10 SNPs identified in intron 3 of the SPBC660.16 gene. A**.** The figure illustrates the positions of the SNPs identified in the third intron of the SPBC660.16 gene. **B.** The sequences shown in A were analysed as in Figure S6. The numbers refer to the haplotypes indicated in A. The areas of the individual nodes are proportional to the numbers of compound haplotypes that contain the particular haplotype. The colors refer to the geographical origins of the respective haplotypes as indicated. The numbers of mutation separating the respective nodes are proportional to the distances between them.

Table S1 Strains used in this study

Strain number	Source-Collection	Date of isolation	Source	Location isolated
UWOPS 92.229.4	MA Lachance UWOPS	unknown to us	Tequilla	Mexico, Jalisco
UWOPS 94.422.2	MA Lachance UWOPS	unknown to us	Tequilla	Mexico, Jalisco
UFMG 790=CBS10459	Carlos Rosa UFMG	1996	Must of Brazilian cachaça;	Brazil:Vicosa
UFMG R435=CBS10458	Carlos Rosa UFMG	1999	Frozen pulp of Eugenia uniflora;	Brazil: Aracaju
UFMG A1263=CBS10469	Carlos Rosa UFMG	1996	Must of Brazilian cachaça;	Brazil:Vicosa
UFMG A602=CBS10460	Carlos Rosa UFMG	1996	Must of Brazilian cachaça;	Brazil: Belo Horizonte
UFMG R420=CBS10472	Carlos Rosa UFMG	1996	Frozen pulp of Eugenia uniflora	Brazil; Aracaju
UFMG A1153=CBS10468	Carlos Rosa UFMG	1996	Must of Brazilian cachaça;	Brazil: Salinas
UFMG R434=CBS10476	Carlos Rosa UFMG	1999	Frozen pulp of Eugenia uniflora	Brazil: Aracaju
UFMG A826=CBS10465	Carlos Rosa UFMG	1996	Must of Brazilian cachaça;	Brazil: Belo Horizonte
UFMG R416=CBS10470	Carlos Rosa UFMG	1999	Frozen pulp of Eugenia uniflora	Brazil: Aracaju
UFMG R428=CBS10475	Carlos Rosa UFMG	1999	Frozen pulp of Eugenia uniflora	Brazil: Aracaju
UFMG A1152=CBS10467	Carlos Rosa UFMG	1996	Must of Brazilian cachaça;	Brazil: Salinas
UFMG R424=CBS10473	Carlos Rosa UFMG	1999	Frozen pulp of Eugenia uniflora	Brazil: Aracaju
UFMG A571=CBS10463	Carlos Rosa UFMG	1996	Must of Brazilian cachaça;	Brazil: Belo Horizonte
UFMG A529=CBS10462	Carlos Rosa UFMG	1996	Must of Brazilian cachaça;	Brazil: Belo Horizonte
UFMG A1000=CBS10465	Carlos Rosa UFMG	1996	Must of Brazilian cachaça;	Brazil: Belo Horizonte
UFMG R427=CBS10474	Carlos Rosa UFMG	1999	Frozen pulp of Eugenia uniflora	Brazil: Aracaju
UFMG R437=CBS10477	Carlos Rosa UFMG	1999	Frozen pulp of Eugenia uniflora	Brazil: Aracaju
UFMG R418=CBS10471	Carlos Rosa UFMG	1999	Frozen pulp of Eugenia uniflora	Brazil: Aracaju
UFMG A521=CBS10461	Carlos Rosa UFMG	1996	Must of Brazilian cachaça;	Brazil: Belo Horizonte
UFMG A738=CBS10464	Carlos Rosa UFMG	1996	Must of Brazilian cachaça;	Brazil: Belo Horizonte
NCYC 2387=DBVPG 6275 = CBS5557 (T of Schiz. malidevorans)	Steve James NCYC	11/11/1985	Listan grapes	Spain
NCYC 2355-1*	Steve James NCYC	1991	Steve James NCYC	Japan
CBS 356 =DBVPG6277 (T of Schiz. pombe var. pombe)	Steve James NCYC	1922	Arak (aniseed-flavoured distilled alcoholic drink)	Eastern mediterraenen

NCYC 380= CBS 10392	Steve James NCYC	1953	Raw cane sugar	Unknown
NCYC 132= CBS 10391	Steve James NCYC	1921	Millet Beer	
NCYC 936= CBS 10394	Steve James NCYC	1979	Toddy (Palm wine)	Sri Lanka
NCYC 683 = CBS 10393	Steve James NCYC	1966	Fermenting apple juice	Unknown
DBVPG6699-1*	Gianni Litti	01/04/1990	Lychee fruit	Indochina
Y0036	Neil Jolly ARC Infruitec	unknown to us	Wine	South Africa
Y0037	Neil Jolly ARC Infruitec	unknown to us	Wine	South Africa
CBS2628	Steve James NCYC	1952	Palm wine	Pakistan
CBS2775	Steve James NCYC	1957	Fermenting molasses	Japan
CBS5680=DBVPG6448	Steve James NCYC	1965	Apple	Poland
CBS5682=DBVPG6376	Steve James NCYC	1965	Bantu beer	South Africa
CBS7335	Steve James NCYC	1988	Alpechín	Spain
NCYC535 = CBS 4100	Steve James NCYC	1959	Unknown	Unknown
DBVPG2801	Steve James NCYC	1939	Lagby (drink from date Palm)	Tunisia
DBVPG2804	Steve James NCYC	1963	Wine	Malta
DBVPG2805	Steve James NCYC	1963	Wine	Malta
DBVPG2806	Steve James NCYC	1963	Wine	Malta
DBVPG2807	Steve James NCYC	1963	Grape must treated with SO2	Malta
DBVPG2808	Steve James NCYC	1963	Grape must treated with SO2	Malta
DBVPG2809	Steve James NCYC	1963	Grape must treated with SO2	Malta
DBVPG2810	Steve James NCYC	1963	Wine	Malta
DBVPG2811	Steve James NCYC	1966	Grape must treated with SO2	Sicily, Italy
DBVPG2812	Steve James NCYC	1966	Grape must treated with SO2	Sicily, Italy
DBVPG2814	Steve James NCYC	1966	Grape must treated with SO2	Sicily, Italy
DBVPG2815	Steve James NCYC	1966	Grape must treated with SO2	Sicily, Italy
DBVPG2816	Steve James NCYC	1966	Grape must treated with SO2	Sicily, Italy
DBVPG2817	Steve James NCYC	1966	Grape must treated with SO2	Sicily, Italy
DBVPG2818	Steve James NCYC	1966	Grape must treated with SO2	Sicily, Italy
DBVPG4433*	Steve James NCYC	12/04/1996	Unknown	Germany
DBVPG4435	Steve James NCYC	12/04/1996	Unknown	Italy
DBVPG4437	Steve James NCYC	12/04/1996	Unknown	Italy
DBVPG6610	Steve James NCYC	08/08/1986	Lab strain lys1	Unknown
Y468	Gert Marais CAMS	unknown to us	Cape wines	South Africa
Y469	Gert Marais CAMS	unknown to us	Unknown	Unknown
Y470	Gert Marais CAMS	unknown to us	Unknown	Unknown
Y831	Gert Marais CAMS	unknown to us	Industrial glucose;	South Africa
Y832	Gert Marais CAMS	unknown to us	Industrial glucose:	South Africa
CBS374 = DBVPG6418	Gert Marais CAMS	1928	Molasses	Delft
972	Jacky Hayles CRUK	1947	Rotten wine	France
975	Jacky Hayles CRUK	1947	Rotten wine	France
DBVPG 6275 = CBS5557 (T of Sciz maledivorans)	Steve James NCYC	11/11/1985	Listan grapes	Spain

DBVPG6279= CBS1042 (T of Schiz. liquefaciens)	Steve James NCYC	11/11/1985	Sulfited grape juice	Unknown
DBVPG6281=CBS1061	Steve James NCYC	11/11/1985	Cane-sugar molasses	Unknown
DBVPG6417=CBS355	Steve James NCYC	10/16/1987	Cane-sugar molasses	Unknown
DBVPG6447=CBS1043	Steve James NCYC	10/31/1987	Cane-sugar molasses	Unknown
DBVPG 6449=CBS1062	Steve James NCYC	10/31/1987	Cane-sugar molasses	Unknown
CBS358=DPVPG6374	Vincent Robert CBS	1922	Unknown	Unknown
CBS2777	Vincent Robert CBS	1957	Fermenting molasses	Japan
CBS1058	Vincent Robert CBS	1949	Molasses	Java, Indonesia
CBS357=DBVPG6280	Vincent Robert CBS	1912	Cane-sugar molasses	Jamaica
CBS2776	Vincent Robert CBS	1957	Fermenting molasses	Japan
CBS 352= DBVPG 6373	Vincent Robert CBS	1923	Batavian arrak factory	Indonesia
CBS1057= DBVPG6375	Vincent Robert CBS	1933	Brewer's yeast	Skane, Sweden
CBS1059	Vincent Robert CBS	1949	Cane sugar	Mauritius
CBS1044	Vincent Robert CBS	1927	Cane-sugar molasses	Unknown
L2470	Giani Litti	unknown to us	Wine	Chile
AWRI141	Eveline Bartowsky AWRI	25/06/1945	Vineyard	Eden Valley, South Australia
AWRI442	Eveline Bartowsky AWRI	1951	Haslegrove Wines	McLaren Vale, South Australia
CBS1063=DBVPG6450	Vincent Robert CBS	1934	Cane-sugar molasses	Unknown
Y468-subclone WRAB NOTT133				
Y468-subclone WRAB NOTT134				
Y468-subclone WRAB NOTT135				
Y468-subclone WRAB NOTT136				
Y470-subclone WRAB NOTT137				
Y470-subclone WRAB NOTT138				
Y470-subclone WRAB NOTT139				
Y470-subclone WRAB NOTT140				
Y831-subclone WRAB NOTT141				
Y831-subclone WRAB NOTT142				
Y832-subclone WRAB NOTT143				
Y832-subclone WRAB NOTT144				
Y832-subclone WRAB NOTT145				
Y832-subclone WRAB NOTT146				
Y468-subclone WRAB NOTT147				
CBS374-subclone WRAB NOTT148				

* DBVPG 6699 is listed as *Saccharomyces boulardi* however GL found the original isolate to be impure, sub-cloned it and found it to contain a yeast resembling *S. pombe* which we have listed here as DBVPG 6699-1. DBVPG 4433 is listed as *S. pombe* in the DBVPG catalogue which we have confirmed here, however it is also listed as being identical to NCYC 3092 which is listed as *Zygsaccharomyces*. We have not established the source of this discrepancy.

Table S2 Assorting 84 strains of *S. pombe* into 40 groups with shared haplotypes.

Strain	hap. no.	660	TER1	TER1+30	TER1+100	CC2L	CC2R	CCC2
UWOPS 92.229.4	**1**	1	1	1	1	1	1	1
**UWOPS 94.422.2**	**2**	2	2	2	1	1	1	1
UFMG A529, UFMG 790, UFMG A826	**3**	3	3	3	1	1	2	1
UFMG R416, UFMG R418, UFMG R420, UFMG R424, UFMG R435	**4**	3	4	1	2	1	1	1
UFMG R427	**5**	3	4	1	2	2	3	3
UFMG A1263	**6**	3	5	3	1	1	2	1
UFMG A521, UFMG A571, UFMG A602	**7**	3	3	3	1	2	1	1
UFMG A1000, UFMG A1153,	**8**	3	6	1	1	2	1	1
UFMG R434	**9**	3	6	1	2	3	1	2
UFMG R428	**10**	3	7	2	2	2	3	1
UFMG A1152	**11**	3	8	1	1	2	1	1
UFMG R437	**12**	3	6	1	2	1	1	1
UFMG A738	**13**	3	3	1	1	1	2	1
NCYC 683, NCYC 2387, DBVPG4435, AWRI 442	**14**	4	6	1	1	1	1	1
NCYC 936, NCYC 2355-1,	**15**	3	9	4	1	4	4	4
**CBS 356, NCYC 132, NCYC535, DBVPG2817, DBVPG4437, AWRI 141**	**16**	5	3	2	1	5	5	5
NCYC 380, CBS 1063, **DBVPG 6281**, CBS 355, **DBVPG 6417**	**17**	3	6	1	2	2	1	1
DBVPG4433, DBVPG 6279, DBVPG6610, DBVPG 6699, Y0036, Y0037, CRUK 972, CRUK 975, Y468, Y469,	**18**	4	6	1	2	2	6	6
CBS 2628	**19**	6	10	4	3	4	7	7
CBS 2775, CBS 2776, **CBS 2777**	**20**	3	11	2	1	1	1	1
CBS 5680	**21**	4	7	2	2	2	1	1
CBS 5682	**22**	7	6	2	1	1	1	1
CBS 7335	**23**	8	12	1	4	1	1	1
DBVPG2801	**24**	3	3	1	5	2	3	8
DBVPG2805	**25**	3	6	1	2	2	6	6
DBVPG2804, DBVPG2806, DBVPG2807, DBVPG2808, DBVPG2809	**26**	4	6	1	1	2	6	6
DBVPG2810	**27**	4	13	5	1	2	6	6
DBVPG2811, DBVPG2812, DBVPG2814, DBVPG2815, DBVPG2816, DBVPG2818	**28**	4	6	1	4	2	6	6
**Y470***	**29**	4	6	5	1	1	6	1
**Y831*, Y832***	**30**	3	6	2	1	2	6	9
CBS374	**31**	3	7	2	2	2	6	6
DBVPG 6447, DBVPG 6449	**32**	3	6	2	1	2	1	1
CBS 358	**33**	3	6	1	1	1	1	1

CBS 1058	**34**	4	6	1	1	2	3	10
CBS 357	**35**	3	14	2	1	1	1	1
CBS 352	**36**	9	15	6	1	1	1	1
CBS 1057	**37**	4	6	1	2	2	1	1
CBS 1059	**38**	4	6	1	2	1	1	1
CBS 1044	**39**	3	16	1	1	2	1	1
L2470	**40**	4	13	5	1	1	1	1

Highlighted in bold are strains that contain translocations detected in the survey. Underlined are strains with the 2.3Mb derived inversion of chromosome I originally identified in CRUK 972. The asterisks indicated strains containing mixtures of karyotypes.

Sequences of haplotypes shown in table S2.

660.16 intron

```
>1
TAAAGCTACAATGATGTTAGCATATTCACATTCGATATGATACAAATAATGTTAAAACAAGGAAAGGTTA
ATGCTCATGAGCCCGGGAAAGCAAGCAAAAAATAAAGACAAAATAATCATAAGTGTGAGTGACAGACAAA
AACAAGATGAACCGAAAACTCCCCGCGAGAGAATCTGATAACGAAATTTTTACCCAACAAACCGGAACGC
CGTGCCATAACAAGTGAGAAAAACTAGGATTGTTTTTCTTACATGCAAAACTCGGACATTTTTGATCACG
ATGCAAAAATAGGGCGCGGGTAAGAAAGAGACAAAAACAAATCGTCGGATAAGTTGATGAGTCATCGTTT
TCGAGGGTCGCTCGTTATCATAGACACATCTACGCTATAACAACACAAAAAGCATGGTAAATGGACAAAA
CCAACACACGCGACTCACCTTACAACGTAGCGCAGTGGAATAACGCAATAGCTATGCAAACGTTATACCA
ACAGAGCAAAAACATTGTAAGAATACACATGCCCGACACAACGGATGGTTGCAAAGAAATCGAGAGTGAA
ACCGCATCGATTACAAACAGGTTTTGAAGGAGCAAACCCGAAAGCAAGAGAAAAACCTTTTCCCATAGCT
ATGTGTTATGCCATATTGAAGAGTAGAAAATGGGAAAGAAATAGCATTTCTAATAAAGAAAGATACTTGT
CTTTGCTTGCTTTCACTTCGATACAAATGTTTTATTATGTCTT

>2
TAAAGCTACAATGATGTTAGCATATTCACATTCGATATGATACAAATAATGTTAAAACAAGGAAAGGTTA
ATGCTCATGAGCCCGGGAAAGCAAGCAAAAAATAAAGACAAAATAATCATAAGTGTGAGTGACAGACAAA
AACAAGATGAACCGAAAACCCCCCGCGAGAGAATCTGATAACGAAATTTTTACCCAACAAACCGGAACGC
CGTGCCATAACAAGTGAGAAAAACTAGGATTGTTTTTCTTACATGCAAAACTCGGACATTTTTGATCACG
ATGCAAAAATAGGGCGCGGGTAAGAAAGAGACAAAAACAAATCGTCGGATAAGTTGATGAGTCATCGTTT
TCGAGGGTCGCTCGTTATCATAGACACATCTACGCTATAACAACACAAAAAGCATGGTAAATGGACAAAA
CCAACACACGCGACTCACCTTACAACGTAGCGCAGTGGAATAACGCAATAGCTATGCAAACGTTATACCA
ACAGAGCAAAAACATTGTAAGAATACACATGCCCGACACAACGGATGGTTGCAAAGAAATCGAGAGTGAA
ACCGCATCGATTACAAACAGGTTTTGAAGGAGCAAACCCGAAAGCAAGAGAAAAACATTTTCCCATAGCT
ATGTGTTATGCCATATTGAAGAGTAGAAAATGGGAAAGAAATAGCATTTCTAATAAAGAAAGAAACCTGT
CTTTGCTTGCTTTCACTTCGATACAAATGTTTTATTATGTCTT

>3
TAAAGCTACAATGATGTTAGCATATTCACATTCGATATGATACAAATAATGTTAAAACAAGGAAAGGTTA
ATGCTCATGAGCCCGGGAAAGCAAGCAAAAAATAAAGACAAAATAATCATAAGTGTGAGTGACAGACAAA
AACAAGATGAACCGAAAACTCCCCGCGAGAGAATCTGATAACGAAATTTTTACCCAACAAACCGGAACGC
CGTGCCATAACAAGTGAGAAAAACTAGGATTGTTTTTCTTACATGCAAAACTCGGACATTTTTGATCACG
ATGCAAAAATAGGGCGCGGGTAAGAAAGAGACAAAAACAAATCGTCGGATAAGTTGATGAGTCATCGTTT
TCGAGGGTCGCTCGTTATCATAGACACATCTACGCTATAACAACACAAAAAGCATGGTAAATGGACAAAA
CCAACACACGCGACTCACCTTACAACGTAGCGCAGTGGAATAACGCAATAGCTATGCAAACGTTATACCA
ACAGAGCAAAAACATTGTAAGAATACACATGCCCGACACAACGGATGGTTGCAAAGAAATCGAGAGTGAA
ACCGCATCGATTACAAACAGGTTTTGAAGGAGCAAACCCGAAAGCAAGAGAAAAACCTTTTCCCATAGCT
ATGTGTTATGCCATATTGAAGAGTAGAAAATGGGAAAGAAATAGCATTTCTAATAAAGAAAGAAACCTGT
CTTTGCTTGCTTTCACTTCGATACAAATGTTTTATTATGTCTT

>4
TAAAGCTACAATGATGTTAGCATATTCACATTCGATATGATACAAATAATGTTAAAACAAGGAAAGGTTA
ATGCTCATGAGCCCGGGAAAGCAAGCAAAAAATAAAGACAAAATAATCATAAGTGTGAGTGACAGACAAA
AACAAGATGAACCGAAAACTCCCCGCGAGAGAATCTGATAACGAAATTTTTACCCAACAAACCGGAACGC
CGTGCCATAACAAGTGAGAAAAACTAGGATTGTTTTTCTTACATGCAAAACTCGGACATTTTTGATCACG
ATGCAAAAATAGGGCGCGGGTAAGAAAGAGACAAAAACAAATCGTCGGATAAGTTGATGAGTCATCGTTT
TCGAGGGTCGCTCGTTATCATAGACACATCTACGCTATAACAACACAAAAAGCATGGTAAATGGACAAAA
CCAACACACGCGACTCACCTTACAACGTAGCGCAGTGGAATAACGCAATAGCTATGCAAACGTTATACCA
ACAGAGCAAAAACATTGTAAGAATACACATGCCCGACACAACGGATGGTTGCAAAGAAATCGAGAGTGAA
ACCGCATCGATTACAAACAGGTTTTGAAGGAGCAAACCCGAAAGCAAGAGAAAAACATTTTCCCATAGCT
ATGTGTTATGCCATATTGAAGAGTAGAAAATGGGAAAGAAATAGCATTTCTAATAAAGAAAGAAACCTGT
CTTTGCTTGCTTTCACTTCGATACAAATGTTTTATTATGTCTT
```

>5

TAAAGCTACAATGATGTTAGCATATTCACATTCGATATGATACAAATAATGTTAAAACAAGGAAAGGTTA
ATGCTCATGAGCCCGGGAAAGCAAGCAAAAAATAAAGACAAAATAATCATAAGTGTGAGTGACAGACAAA
AACAAGATGAACCGAAAACTCCCCGCGAGAAAATCTGATAACGAAATTTTTACCCAACAAACCGGAACGC
CGTGCCATAACAAGTGAGAAAAACTAGGATTGTTTTTCTTACATGCAAAACTCGGACATTTTTGATCACG
ATGCAAAAATAGGGCGCGGGTAAGAAAGAGACAAAAACAAATCGTCGGATAAGTTGATGAGTCATCGTTT
TCGAGGGTCGCTCGTTATCATAGACACATCTACGCTATAACAACACAAAAAGCATGGTAAATGGACAAAA
CCAACACACGCGACTCACCTTACAACGTAGCGCAGTGGAATAACGCAATAGCTATGCAAACGTTATACCA
ACAGAGCAAAAACATTGTAAGAATACACATGCCCGACACAACGGATGGTTGCAAAGAAATCGAGAGTGAA
ACCGCATCGATTACAAACAGGTTTTGAAGGAGCAAACCCGAAAGCAAGAGAAAAACCTTTTCCCATAGCT
ATGTGTTATGCCATATTGAAGAGTAGAAAATGGGAAAGAAATAGCATTTCTAATAAAGAAAGAAACCTGT
CTTTGCTTGCTTTCACTTCGATACAAATGTTTTATTATGTCTT

>6
TAAAGCTACAATGATGTTAGCATATTCACATTCGATATAATACAAATAATGTTAAAACAAGGAAATGTTA
ATGCTCATGAGCCCGGGAAAGCAAGCAAAAAATAAAGACAAAATAATCATAAGTGTGAGTGACAGACAAA
AACAAGATGAACCGAAAACTCCCCGCGAGAGAATCTGATAACGAAATTTTTACCCAACAAACCGGAACGC
CGTGCCATAACAAGTGAGAAAAACTAGGATTGTTTTTCTTACATGCAAAACTCGAACATTTTTGATCACG
ATGCAAAAATAGGGCGCGGGTAAGAAAGAGACAAAAACAAATCGTCGGATAAGTTGATGAGTCATCGTTT
TCGAGGGTCGCTCGTTATCATAGACACATCTACGCTATAACAACACAAAAAGCATGGTAAATGGACAAAA
CCAACACACGCGACTCACCTTACAACGTAGCGCAGTGGAATAACGCAATAGCTATGCAAACGTTATACCA
ACAGAGCAAAAACATTGTAAGAATACACATGCCCGACACAACGGATGGTTGCAAAGAAATCGAGAGTGAA
ACCGCATCGATTACAAACAGGTTTTGAAGGAGCAAACCCGAAAGCAAGAGAAAAACCTTTTCCCATAGCT
ATGTGTTATGCCATATTGAAGAGTAGAAAATGGGAAAGAAATAGCATTTCTAATAAAGAAAGAAACCTGT
CTTTGCTTGCTTTCACTTCGATACAAATGTTTTATTATGTCTT

>7
TAAAGCTACAATGATGTTAGCATATTCACATTCGATATGATACAAATAATGTTAAAACAAGGAAAGGTTA
ATGCTCATGAGCCCGGGAAAGCAAGCAAAAAATAAAGACAAAATAATCATAAGTGTGAGTGACAGACAAA
AACAAGATGAACCGAAAACTCCCCGCGAGAGAATCTGATAACGAAATTTTTACCCAACAAACCGGAATGC
CGTGCCATAACAAGTGAGAAAAGCTAGGATTGTTTTTCTTACATGCAAAACTCGGACATTTTTGATCACG
ATGCAAAAATAGGGCGCGGGTAAGAAAGAGACAAAAACAAATCGTCGGATAAGTTGATGAGTCATCGTTT
TCGAGGGTCGCTCGTTATCATAGACACATCTACGCTATAACAACACAAAAAGCATGGTAAATGGACAAAA
CCAACACACGCGACTCACCTTACAACGTAGCGCAGTGGAATAACGCAATAGCTATGCAAACGTTATACCA
ACAGAGCAAAAACATTGTAAGAATACACATGCCCGACACAACGGATGGTTGCAAAGAAATCGAGAGTGAA
ACCGCATCGATTACAAACAGGTTTTGAAGGAGCAAACCCGAAAGCAAGAGAAAAACCTTTTCCCATAGCT
ATGTGTTATGCCATATCGAAGAGTAGAAAATGGGAAAGAAATAGCATTTCTAATAAAGAAAGAAACCTGT
CTTTGCTTGCTTTCACTTCGATACAAATGTTTTATTATGTCTT

>8
TAAAGCTACAATGATGTTAGCATATTCACATTCGATATGATACAAATAATGTTAAAACAAGGAAAGGTTA
ATGCTCATGAGCCCGGGAAAGCAAGCAAAAAATAAAGACAAAATAATCATAAGTGTGAGTGACAGACAAA
AACAAGATGAACCGAAAACTCCCCGCGAGAGAATCTGATAACGAAATTTTTACCCAACAAACCGGAACGC
CGTGCCATAACAATTGAGAAAAACTAGGATTGTTTTTCTTACATGCAAAACTCGGACATTTTTGATCACG
ATGCAAAAATAGGGCGCGGGTAAGAAAGAGACAAAAACAAATCGTCGGATAAGTTGATGAGTCATCGTTT
TCGAGGGTCGCTCGTTATCATAGACACATCTACGCTATAACAACACAAAAAGCATGGTAAATGGACAAAA
CCAACACACGCGACTCACCTTACAACGTAGCGCAGTGGAATAACGCAATAGCTATGCAAACGTTATACCA
ACAGAGCAAAAACATTGTAAGAATACACATGCCCGACACAACGGATGGTTGCAAAGAAATCGAGAGTGAA
ACCGCATCGATTACAAACAGGTTTTGAAGGAGCAAACCCGAAAGCAAGAGAAAAACATTTTCCCATAGCT
ATGTGTTATGCCATATTGAAGAGTAGAAAATGGGAAAGAAATAGCATTTCTAATAAAGAAAGAAACCTGT
CTTTGCTTGCTTTCACTTCGATACAAATGTTTTATTATGTCTT

>9
TAAAGCTACAATGATGTTAGCATATTCACATTCGATATAATACAAATAATGTTAAAACAAGGAAATGTTA

ATGCTCATGAGCCCGGGAAAGCAAGCAAAAAATAAAGACAAAATAATCATAAGTGTGAGTGACAGACAAA
AACAAGATGAACCGAAAACTCCCCGCGAGAGAATCTGATAACGAAATTTTTACCCAACAAACCGGAACGC
CGTGCCATAACAAGTGAGAAAAACTAGGATTGTTTTTCTTACATGCAAAACTCGAACATTTTTGATCACG
ATGCAAAAATAGGGCGCGGGTAAGAAAGAGACAAAAACAAATCGTCGGATAAGTTGATGAGTCATCGTTT
TCGAGGGTCGCTCGTTATCATAGACACATCTACGCTATAACAACACAAAAAGCATGGTAAATGGACAAAA
CCAACACACGCGACTCACCTTACAACGTAGCGCAGTGGAATAACGCAATAGCTATGCAAACGTTATACCA
ACAGAGCAAAAACATTGTAAGAATACACATGCCCGACACAACGGATGGTTGCAAAGAAATCGAGAGTGAA
ACCGCATCGATTACAAACAGGTTTTGAAGGAGCAAACCCGAAAGCAAGAGAAAAACCTTTTCCCATAGCT
ATGTGTTATGCCATATTGAAGAGTAGAAAATGGGAAAGAAATAGCATTTCTAATAAAGAAAGAGACCTGT
CTTTGCTTGCTTTCACTTCGATACAAATGTTTTATTATGTCTT

TER1

>1
ACAGATACAATTAAGAATAACTTGTAATGGTATTCACATTACTAATAAACTTCTTAATTCGTCTGTTTGG
GTATTCATTACATATTCATCAACTAAGCAAGTCTTACATGCGTCCAAATTTGTTTAAGACAAGAAAATAT
ACCATCTATGCTTAATAAACAAACTTCATCTCTTCTAGTACGCAAATAAATACATTAAATTTATTTTACA
TTATAATAAGATTCATCACTTTCTCAAAATTTTGAAACCGGAAACGGAATTCAGCATGTTTTAATAAAAA
GATGAGCAACCGCGTTCTTACTAAATACGCAATTCTAATAATTTGGGAATTGTAGTTAGTAATTTTAAAT
TTAATCATTAAAACATACCAAAAAATGGGCCCGTTCCTTTTAACATTTTGCTCAGACCAAGTGAAAAAAA
CCTAAAACGCGCTCAAGAGGAACAACGGACGAGCTACACTCAAAATATTAATGAATGGGTAGTCAACTAT
TTTACTGGCGAACAAGCAAAAGCTTTGATCCATGGATCTCACGTAATGTAAAATAGTATCTTCCCTAAGC
ATTATTTATTTGAGAAACAGGACGTTCATTATTGTTTGCCGCAAAGAATCCAAGTTCAAGGAAATTGAAT
ACAGACAAGAGTTGCTATTCGTTTGAATGTACATTGTGACCACAAGTCGGTTTTTGTTTCCTTCCAAATA
GTACCTTCAACACGAAGTTGTACATTTAGCACGGACAAAGAATCGACCAAAAAACCAAAATTAAATGTAG
AAGGAAAACCTTCAAGTCATTTTTTTTATAATAGCTCTTTTAAGTTCCATACTTTTTATCTCCGTAACAA
ATGCCAAAAGAAGCTAGTATAGACGAGAGCATTCAGCCAACAAATTTGCACAAGCAAAAATTGTTACCAA
TACAATTTAATATTTGTATGGGGAATTGAAACTATCTATAAAACTTTCTTCATTGCTTGATTTTTAGTAA
CCTTCAGTAATCAAACGAGACTCTTCTACCAAAAATTCGTTGTGATCTGACAAGCACTTAAATGATGGAT
ACAGAATTTATACAAAAGAAAGGTAGGATTAAACTGTAAATGAACTTTAAAGGCAGAAGACTCACGTACA
CTACACCCAGCGATTCAATTGATGTCCAGGCCTCTTTTTTCCTACTGTATACAGAAGTATGAAGTCTGCA
GCCATAATAAAAGCGGTAGGAATGATCTATGTTTTCCGCAACGCGTCAACGATTCCATGAAGAGCCTCTC
TTATGAATGTTCCTAATGATCAAAACTCTAAAAAAAGCTAAAAAGAATAATTTCGCAATGCTGAAAAGTT
GAAGAGTACGGTTACACGAAGCAGTACAGGAATTCGAAATCCATCGAAAACAACTATCCAGACAAACTGA
AAAGCTAAACAGACTACTCGTTCTTTCTAGTAGACAGTATACAGGTTTTGGAATAGCAAACTATACTTAA
GGCCTATGAATCATTTCCTTCAAACTTTCAAATCAATCACCAAGTCCTCAAGAACCGTTTGATTAATTTT
CCTTGTCAACCTTCTAAGCATGGGCGTTGCGTTGAGTATAAACAAAACAGCAATAAATCCGAGTATACAT
GAATTGTTTCCTTCTTCAAACAAAACGCTGTTACCTTTGCGCGTATGATGACTTCCCATACTTACAAAAA
TTTTCAAAACCTAATA

>2
ACAGATACAATTAAGAATAACTTGTAATGGTATTCACATTACTAATAAACTTCTTAATTCATCTGTTTGG
GTATTCATTACATATTCATCAACTAAGCAAGTCTTACATGCGTCCAAATTTGTTTAAGACAAGAAAATAT
ACCATCTATGCTTAATAAACAAACTTCATCTCTTCTAGTACGCAAATAAATACATTAAATTTATTTTACA
TTATAATAAGATTCATCACTTTCTCAAAATTTTGAAACCGGAAACGGAATTCAGCATGTTTTAATAAAAA
GATGAGCAACCGCGTTCTTACTAAATACGCAATTCTAATAATTTGGGAATTGTAGTTAGTAATTTTAAAT
TTAATCATTAAAACATACCAAAAAATGGGCCCGTTCCTTTTAACATTTTGCTCAGACCAAGTGAAAAAAA
CCTAAAACGCGCTCAAGAGGAACAACGGACGAGCTACACTCAAAATATTAATGAATGGGTAGTCAACTAT
TTTACTAGCGAACAAGCAAAAGCTTTGATCCATGGATCTCACGTAATGTAAAATAGTATCTTCCCTAAGC
ATTAATTATTTGAGAAACAGGACGTTCATTATTGTTTGCCGCAAAGAATCCAAGTTCAAGGAAATTGAAT
ACAGACAAGAGTTGCTATTCGTTTGAATGTACATTGTGACCACAAGTCGGTTTTTGTTTCCTTCCAAATA
GTACCTTCAACACGAAGTTGTACATTTAGCACGGACAAAGAATCGACCAAAAAACCAAAATTAAATGTAG
AAGGAAAACCTTCAAGTCATTTTTTTTATAATAGCTCTTTTAAGTTCCATACTTTTTATCTCCGTAACAA
ATGCCAAAAGAAGCTAGTATAGACGAGAGCATTCAGCCAACAAATTTGCACAAGCAAAAATTGTTACCAA
TACAATTTAATATTTGTATGGGGAATTGAAACTATCTATAAAACTTTCTTCATTGCTTGATTTTTAGTAA
CCTTCAGTAATCAAACGAGACTCTTCTACCAAAAATTCGTTGTGATCTGACAAGCACTTAAATGATGGAT
ACAGAATTTATACAAAAGAAAGGTAGGATTAAACTGTAAATGAACTTTAAAGGCAGAAGACTCACGTACA

CTACACCCAGCGATTCAATTGATGTCCAGGCCTCTTTTTTCCTACTGTATACAGAAGTATGAAGTCTGCA
GCCATAATAAAAGCGGTAGGAATGATCTATGTTTTCCGCAACGCGTCAACGATTCCATGAAGAGCCTCTC
TTATGAATGTTCCTAATGATCAAAACTCTAAAAAAAGCTAAAAAGAATAATTTCGCAATGCTGAAAAGTT
GAAGAGTACGGTTACACGAAGCAGTACAGGAATTCGAAATCCATCGAAAACAACTATCCAGACAAACTGA
AAAGCTAAACAGACTACTCGTTCTTTCTAGTAGACAGTATACAGGTTTTGGAATAGCAAACTATACTTAA
GGCCTATGAATCATTTCCTTCAAACTTTCAAATCAATCACCAAGTCCTCAAGAACCGTTTGATTAATTTT
CCTTGTCAACCTTCTAAGCATGGGCGTTGCGTTGAGTATAAACAAAACAGCAATAAATCCGAGTATACAT
GAATTGTTTCCTTCTTCAAACAAAACGCTGTTACCTTTGCGCGTATGATGACTTCCCATACTTACAAAAA
TTTTCAAAACCTAATA

>3
ACAGATACAATTAAGAATAACTTGTAATGGTATTCACATTACTAATAAACTTCTAAATTCATCTGTTTGG
GTATTCATTACATATTCATCAACTAAGCAAGTCTTACATGCGTCCAAATTTGTTTAAGACAAGAAAATAT
ACCATCTATGCTTAATAAACAAACTTCATCTCTTCTAGTACGCAAATAAATACATTAAATTTATTTTACA
TTATAATAAGATTCATCACTTTCTCAAAATTTTGAAACCGGAAACGGAATTCAGCATGTTTTAATAAAAA
GATGAGCAACCGCGTTCTTACTAAATACGCAATTCTAATAATTTGGGAATTGTAGTTAGTAATTTTAAAT
TTAATCATTAAAACATACCAAAAAATGGGCCCGTTCCTTTTAACATTTTGCTCAGACCAAGTGAAAAAAA
CCTAAAACGCGCTCAAGAGGAACAACGGACGAGCTACACTCAAAATATTAATGAATGGGTAGTCAACTAT
TTTACTAGCGAACAAGCAAAAGCTTTGATCCATGGATCTCACGTAATGTAAAATAGTATCTTCCCTAAGC
ATTATTTATTTGAGAAACAGGACGTTCATTATTGTTTGCCGCAAAGAATCCAAGTTCAAGGAAATTGAAT
ACAGACAAGAGTTGCTATTCGTTTGAATGTACATTGTGACCACAAGTCGGTTTTTGTTTCCTTCCAAATA
GTACCTTCAACACGAAGTTGTACATTTAGCACGGACAAAGAATCGACCAAAAAACCAAAATTAAATGTAG
AAGGAAAACCTTCAAGTCATTTTTTTTATAATAGCTCTTTTAAGTTCCATACTTTTTATCTCCGTAACAA
ATGCCAAAAGAAGCTAGTATAGACGAGAGCATTCAGCCAACAAATTTGCACAAGCAAAAATTGTTACCAA
TACAATTTAATATTTGTATGGGGAATTGAAACTATCTATAAAACTTTCTTCATTGCTTGATTTTTAGTAA
CCTTCAGTAATCAAACGAGACTCTTCTACCAAAAATTCGTTGTGATCTGACAAGCACTTAAATGATGGAT
ACAGAATTTATACAAATGAAAGGTAGGATTAAACTGTAAATGAACTTTAAAGGCAGAAGACTCACGTACA
CTACACCCAGCGATTCAATTGATGTCCAGGCCTCTTTTTTCCTACTGTATACAGAAGTATGAAGTCTGCA
GCCATAATAAAAGCGGTAGGAATGATCTATGTTTTCCGCAACGCGTCAACGATTCCATGAAGAGCCTCTC
TTATGAATGTTCCTAATGATCAAAACTCTAAAAAAAGCTAAAAAGAATAATTTCGCAATGCTGAAAAGTT
GAAGAGTACGGTTACACGAAGCAGTACAGGAATTCGAAATCCATCGAAAACAACTATCCAGACAAACTGA
AAAGCTAAACAGACTACTCGTTCTTTCTAGTAGACAGTATACAGGTTTTGGAATAGCAAACTATACTTAA
GGCCTATGAATCATTTCCTTCAAACTTTCAAATCAATCACCAAGTCCTCAAGAACCGTTTGATTAATTTT
CCTTGTCAACCTTCTAAGCATGGGCGTTGCGTTGAGTATAAACAAAACAGCAATAAATCCGAGTATACAT
GAATTGTTTCCTTCTTCAAACAAAACGCTGTTACCTTTGCGCGTATGATGACTTCCCATACATACAAAAA
TTTTCAAAACCTAATA

>4
ACAGATACAATTAAGAATAACTTGTAATGGTATTCACATTACTAATAAACTTCTAAATTCATCTGTTTGG
GTATTCATTACATATTCATCAACTAAGCAAGTCTTACATGCGTCCAAATTTGTTTAAGACAAGAAAATAT
ACCATCTATGCTTAATAAACAAACTTCATCTCTTCTAGTACGCAAATAAATACATTAAATTTATTTTACA
TTATAATAAGATTCATCACTTTCTCAAAATTTTGAAACCGGAAACGGAATTCAGCATGTTTTAATAAAAA
GATGAGCAACCGCGTTCTTACTAAATACGCAATTCTGATAATTTGGGAATTGTAGTTAGTAATTTTAAAT
TTAATCATTAAAACATACCAAAAAATGGGCCCGTTCCTTTTAACATTTTGCTCAGACCAAGTGAAAAAAA
CCTAAAACGCGCTCAAGAGGAACAACGGACGAGCTACACTCAAAATATTAATGAATGGGTAGTCAACTAT
TTTACTAGCGAACAAGCAAAAGCTTTGATCCATGGATCTCACGTAATGTAAAATAGTATCTTCCCTAAGC
ATTATTTTTTTGAGAAACAGGACGTTCATTATTGTTTGCCGCAAAGAATCCAAGTTCAAGGAAATTGAAT
ACAGACCAGAGTTGCTATTCGTTTGAATGTACATTGTGACCACAAGTCGGTTTTTGTTTCCTTCCAAATA
GTACCTTCAACACGAAGTTGTACATTTAGCACGGACAAAGAATCGACCAAAAAACCAAAATTAAATGTAG
AAGGAAAACCTTCAAGTCATTTTTTTTATAATAGCTCTTTTAAGTTCCATACTTTTTATCTCCGTAACAA
ATGCCAAAAGAAGCTAGTATAGACGAGAGCATTCAGCCAACAAATTTGCACAAGCAAAAATTGTTACCAA
TACAATTTAATATTTGTATGGGGAATTGAAACTATCTATAAAACATTCTTCATTGCTTGATTTTTAGTAA
CGTTCAGTAATCAAACGAGACTCTTCTACCAAAAATTCGTTGTGATCTGACAAGCACTTAAATGATGGAT
ACAGATTTTATACAAATGAAAGGTAGGATTAAACTGTAAATGAATTTTAAAGGCAGAAGACTCACGTACA
CTGCACCCAGCGATTCAATTGATGTCCAGGCCTCTTTTTTCCTACTGTATACAGAAGTATGAAGTCTGCA
GCCATAATAAAAGCGGTAGGAATGATCTATGTTTTCCGCAACGCGTCAACGATTCCATGAAGAGCCTCTC
TTATGAATATTCCTAATGATCAAAACTCTAAAAAAAGCTAAAAAGAATAATTTCGCAATGCTGAAAAGTT
GAAGAGTACGGTTACACGAAGCAGTACAGGAATTCGAAATCCATCGAAAACAACTATCCAGACAAACTGA

AAAGCTAAACAGACTACTCGTTCTTTCTAGTAGACAGTATACAGGTTTTGGAATAGCAAACTATACTTAA
GGCCTATGAATCATTTCCTTCAAACTTTCAAATCAATCACCAAGCCCTCAAGAACCGTTTGATTAATTTT
CCTTGTCAACCTTCTAAGCATGGGCGTTGCGTTGAGTATAAACAAAACAGCAATAAATCCGAGTATACAT
GAATTGTTTCCTTCTTCAAACAAAACGCTGTTACCTTTGCGCGTATGATGACTTCCCATACTTACAAAAA
TTTTCAAAACCTAACA

>5
ACAGATACAATTAAGAATAACTTGTAATGGTATTCACATTACTAATAAACTTCTAAATTCATCTGTTTGG
GTATTCATTACATATTCATCAACTAAGCAAGTCTTACATGCGTCCAAATTTGTTTAAGACAAGAAAATAT
ACCATCTATGCTTAATAAACAAACTTCATCTCTTCTAGTACGCAAATAAATACATTAAATTTATTTTACA
TTATAATAAGATTCATCACTTTCTCAAAATTTTGAAACCGGAAACGGAATTCAGCATGTTTTAATAAAAA
GATGAGCAACCGCGTTCTTACTAAATACGCAATTCTGATAATTTGGGAATTGTAGTTAGTAATTTTAAAT
TTAATCATTAAAACATACCAAAAAATGGGCCCGTTCCTTTTAACATTTTGCTCAGACCAAGTGAAAAAAA
CCTAAAACGCGCTCAAGAGGAACAACGGACGAGCTACACTCAAAATATTAATGAATGGGTAGTCAACTAT
TTTACTAGCGAACAAGCAAAAGCTTTGATCCATGGATCTCACGTAATGTAAAATAGTATCTTCCCTAAGC
ATTATTTTTTTGAGAAACAGGACGTTCATTATTGTTTGCCGCAAAGAATCCAAGTTCAAGGAAATTGAAT
ACAGACCAGAGTTGCTATTCGTTTGAATGTACATTGTGACCACAAGTCGGTTTTTGTTTCCTTCCAAATA
GTACCTTCAACACGAAGTTGTACATTTAGCACGGACAAAGAATCGACCAAAAAACCAAAATTAAATGTAG
AAGGAAAACCTTCAAGTCATTTTTTTTATAATAGCTCTTTTAAGTTCCATACTTTTTATCTCCGTAACAA
ATGCCAAAAGAAGCTAGTATAGACGAGAGCATTCAGCCAACAAATTTGCACAAGCAAAAATTGTTACCAA
TACAATTTAATATTTGTATGGGGAATTGAAACTATCTATAAAACTTTCTTCATTGCTTGATTTTTAGTAA
CGTTCAGTAATCAAACGAGACTCTTCTACCAAAAATTCGTTGTGATCTGACAAGCACTTAAATGATGGAT
ACAGATTTTATACAAATGAAAGGTAGGATTAAACTGTAAATGAATTTTAAAGGCAGAAGACTCACGTACA
CTGCACCCAGCGATTCAATTGATGTCCAGGCCTCTTTTTTCCTACTGTATACAGAAGTATGAAGTCTGCA
GCCATAATAAAAGCGGTAGGAATGATCTATGTTTTCCGCAACGCGTCAACGATTCCATGAAGAGCCTCTC
TTATGAATATTCCTAATGATCAAAACTCTAAAAAAAGCTAAAAAGAATAATTTCGCAATGCTGAAAAGTT
GAAGAGTACGGTTACACGAAGCAGTACAGGAATTCGAAATCCATCGAAAACAACTATCCAGACAAACTGA
AAAGCTAAACAGACTACTCGTTCTTTCTAGTAGACAGTATACAGGTTTTGGAATAGCAAACTATACTTAA
GGCCTATGAATCATTTCCTTCAAACTTTCAAATCAATCACCAAGCCCTCAAGAACCGTTTGATTAATTTT
CCTTGTCAACCTTCTAAGCATGGGCGTTGCGTTGAGTATAAACAAAACAGCAATAAATCCGAGTATACAT
GAATTGTTTCCTTCTTCAAACAAAACGCTGTTACCTTTGCGCGTATGATGACTTCCCATACTTACAAAAA
TTTTCAAAACCTAACA

>6
ACAGATACAATTAAGAATAACTTGTAATGGTATTCACATTACTAATAAACTTCTAAATTCATCTGTTTGG
GTATTCATTACATATTCATCAACTAAGCAAGTCTTACATGCGTCCAAATTTGTTTAAGACAAGAAAATAT
ACCATCTATGCTTAATAAACAAACTTCATCTCTTCTAGTACGCAAATAAATACATTAAATTTATTTTACA
TTATAATAAGATTCATCACTTTCTCAAAATTTTGAAACCGGAAACGGAATTCAGCATGTTTTAATAAAAA
GATGAGCAACCGCGTTCTTACTAAATACGCAATTCTAATAATTTGGGAATTGTAGTTAGTAATTTTAAAT
TTAATCATTAAAACATACCAAAAAATGGGCCCGTTCCTTTTAACATTTTGCTCAGACCAAGTGAAAAAAA
CCTAAAACGCGCTCAAGAGGAACAACGGACGAGCTACACTCAAAATATTAATGAATGGGTACTCAACTAT
TTTACTAGCGAACAAGCAAAAGCTTTGATCCATGGATCTCACGTAATGTAAAATAGTATCTTCCCTAAGC
ATTATTTATTTGAGAAACAGGACGTTCATTATTGTTTGCCGCAAAGAATCCAAGTTCAAGGAAATTGAAT
ACAGACAAGAGTTGCTATTCGTTTGAATGTACATTGTGACCACAAGTCGGTTTTTGTTTCCTTCCAAATA
GTACCTTCAACACGAAGTTGTACATTTAGCACGGACAAAGAATCGACCAAAAAACCAAAATTAAATGTAG
AAGGAAAACCTTCAAGTCATTTTTTTTATAATAGCTCTTTTAAGTTCCATACTTTTTATCTCCGTAACAA
ATGCCAAAAGAAGCTAGTATAGACGAGAGCATTCAGCCAACAAATTTGCACAAGCAAAAATTGTTACCAA
TACAATTTAATATTTGTATGGGGAATTGAAACTATCTATAAAACTTTCTTCATTGCTTGATTTTTAGTAA
CCTTCAGTAATCAAACGAGACTCTTCTACCAAGAATTCGTTGTGATCTGACAAGCACTTAAATGATGGAT
ACAGAATTTATACAAATGAAAGGTAGGATTAAACTGTAAATGAACTTTAAAGGCAGAAGACTCACGTACA
CTACACCCAGCGATTCAATTGATGTCCAGGCCTCTTTTTTCCTACTGTATACAGAAGTATGAAGTCTGCA
GCCATAATAAAAGCGGTAGGAATGATCTATGTTTTCCGCAACGCGTCAACGATTCCATGAAGAGCCTCTC
TTATGAATGTTCCTAATGATCAAAACTCTAAAAAAAGCTAAAAAGAATAATTTCGCAATGCTGAAAAGTT
GAAGAGTACGGTTACACGAAGCAGTACAGGAATTCGAAATCCATCGAAAACAACTATCCAGACAAACTGA
AAAGCTAAACAGACTACTCGTTCTTTCTAGTAGACAGTATACAGGTTTTGGAATAGCAAACTATACTTAA
GGCCTATGAATCATTTCCTTCAAACTTTCAAATCAATCACCAAGTCCTCAAGAACCGTTTGATTAATTTT
CCTTGTCAACCTTCTAAGCATGGGCGTTGCGTTGAGTATAAACAAAACAGCAATAAATCCGAGTATACAT
GAATTGTTTCCTTCTTCAAACAAAACGCTGTTACCTTTGCGCGTATGATGACTTCCCATACATACAAAAA

TTTTCAAAACCTAATA

>7
ACAGATACAATTAAGAATAACTTGTAATGGTATTCACATTACTAATAAACTTCTAAATTCATCTGTTTGG
GTATTCATTACATATTCATCAACTAAGCAAGTCTTACATGCGTCCAAATTTGCTTAAGACAAGAAAATAT
ACCATCTATGCTTAATAAACAAACTTCATCTCTTCTAGTACGCAAATAAATACATTAAATTTATTTTACA
TTATAATAAGATTCATCACTTTCTCAAAATTTTGAAACCGGAAACGGAATTCAGCATGTTTTAATAAAAA
GATGAGCAACCGCGTTCGTACTAAATACGCAATTCTAATAATTTGGGAATTGTAGTTAGTAATTTTAAAT
TTAATCATTAAAACATACCAAAAAATGGGCCCGTTCCTTTTAACATTTTGCTCAGACCAAGTGAAAAAAA
CCTAAAACGCGCTCAAGAGGAACAACGGACGAGCTACACTCAAAATATTAATGAATGGGTAGTCAACTAT
TTTACTAGCGAACAAGCAAAAGCTTTGATCCATGGATCTCACGTAATGTAAAATAGTATCTTCCCTAAGC
ATTATTTATTTGAGAAACAGGACGTTCATTATTGTTTGCCGCAAAGAATCCAAGTTCAAGGAAATTGAAT
ACAGACAAGAGTTGCTATTCGTTTGAATGTACATTGTGACCACAAGTCGGTTTTTGTTTCCTTCCAAATA
GTACCTTCAACACGAAGTTGTACATTTAGCACGGACAAAGAATCGACCAAAAAACCAAAATTAAATGTAG
AAGGAAAACCTTCAAGTCATTTTTTTTATAATAGCTCTTTTAAGTTCCATACTTTTTATCTCCGTAACAA
ATGCCAAAAGAAGCTAGTATAGACGAGAGCATTCAGCCAACAAATTTGCACAAGCAAAAATCGTTACCAA
TACAATTTAATATTTGTATGGGGAATTGAAACTATCTATAAAACTTTCTTCATTGCTTGATTTTTAGTAA
CCTTCAGTAATCAAACAAGACTCTTCTACCAAAAATTCGTTGTGATCTGACAAGCACTTAAATGATGGAT
ACAGAATTTATACAAATGAAAGGTAGGATTAAACTGTAAATGAATTTTAAAGGCAGAAGACTCACGTACA
CTACACCCAGCGATTCAATTGATGTCCAGGCCTCTTTTTCCCTACTGTATACAGAAGTATGAAGTCTGCA
GCCATAATAAAAGCGGTAGGAATGATCTATGTTTTCCGCAACGCGTCAACGATTCCATGAAGAGCCTCTC
TTATGAATGTTCCTAATGATCAAAACTCTAAAAAAAGCTAAAAAGAATAATTTCGCAATGCTGAGAAGTT
GAAGAGTACGGTTACACGAAGCAGTACAGGAATTCGAAATCCATCGAAAACAACTATCCAGACAAACTGA
AAAGCTAAACAGACTACTCGTTCTTTCTAGTAGACAGTATACAGGTTTTGGAATAGCAAACTATACTTAA
GGCCTATGAATCATTTCCTTCAAACTTTCAAATCAATCACCAAGCCCTCAAGAACCGTTTGATTAATTTT
CCTTGTCAACCTTCTAAGCATGGGCGTTGCGTTGAGTATAAACAAAACAGCAATAAATCCGAGTATACAT
GAATTGTTTCCTTCTTCAAACAAAACGCTGTTACCTTTGCGCGTATGATGACTTCCCATACTTACAAAAA
TTTTCAAAACCTAATA

>8
ACAGATACAATTAAGAATAACTTGTAATGGTATTCACATTACTAATAAACTTCTAAATTCATCTGTTTGG
GTATTCATTACATATTCATCAACTAAGCAAGTCTTACATGCGTCCAAATTTGTTTGAGACAAGAAAATAT
ACCATCTATGCTTAATAAACAAACTTCATCTCTTCTAGTACGCAAATAAATACATTAAATTTATTTTACA
TTATAATAAGATTCATCACTTTCTCAAAATTTTGAAACCGGAAACGGAATTCAGCATGTTTTAATAAAAA
GATGAGCAACCGCGTTCGTACTAAATACGCAATTCTAATAATTTGGGAATTGTAGTTAGTAATTTTAAAT
TTAATCATTAAAACATACCAAAAAATGGGCCCGTTCCTTTTAACATTTTGCTCAGACCAAGTGAAAAAAA
CCTAAAACGCGCTCAAGAGGAACAACGGACGAGCTACACTCAAAATATTAATGAATGGGTAGTCAATTAT
TTTACTAGCGAACAAGCAAAAGCTTTGATCCATGGATCTCACGTAATGTAAAATAGTATCTTCCCTAAGC
ATTATTTATTTGAGAAACAGGACGTTCATTATTGTTTGCCGCAAAGAATCCAAGTTCAAGGAAATTGAAT
ACAGACAAGAGTTGCTATTCGTTTGAATGTACATTGTGACCACAAGTCGGTTTTTGTTTCCTTCCAAATA
GTACCTTCAACACGAAGTTGTACATTTAGCACGGACAAAGAATCGACCAAAAAACCAAAATTAAATGTAG
AAGGAAAACCTTCAAGTCATTTTTTTTATAATAGCTCTTTTAAGTTCCATACTTTTTATCTCCGTAACAA
ATGCCAAAAGAAGCTAGTATAGACGAGAGCATTCAGCCAACAAATTTGCACAAGCAAAAATCGTTACCAA
TACAATTTAATATTTGTATGGGGAATTGAAACTATCTATAAAACTTTCTTCATTGCTTGATTTTTAGTAA
CCTTCAGTAATCAAACAAGACTCTTCTACCAAAAATTCGTTGTGATCTGACAAGCACTTAAATGATGGAT
ACAGAATTTATACAAATGAAAGGTAGGATTAAACTGTAAATGAATTTTAAAGGCAGAAGACTCACGTACA
CTACACCCAGCGATTCAATTGATGTCCAGGCCTCTTTTTCCCTACTGTATACAGAAGTATGAAGTCTGCA
GCCATAATAAAAGCGGTAGGAATGATCTATGTTTTCCGCAACGCGTCAACGATTCCATGAAGAGCCTCTC
TTATGAATGTTCCTAATGATCAAAACTCTAAAAAAAGCTAAAAAGAATAATTTCGCAATGCTGAGAAGTT
GAAGAGTACGGTTACACGAAGCAGTACAGGAATTCGAAATCCATCGAAAACAACTATCCAGACAAACTGA
AAAGCTAAACAGACTACTCGTTCTTTCTAGTAGACAGTATACAGGTTTTGGAATAGCAAACTATACTTAA
GGCCTATGAATCATTTCCTTCAAACTTTCAAATCAATCACCAAGCCCTCAAGAACCGTTTGATTAATTTT
CCTTGTCAACCTTCTAAGCATGGGCGTTGCGTTGAGTATAAACAAAACAGCAATAAATCCGAGTATACAT
GAATTGTTTCCTTCTTCAAACAAAACGCTGTTACCTTTGCGCGTATGATGACTTCCCATACTTACAAAAA
TTTTCAAAACCTAATA

>9
ACAGATACAATTAAGAATAACTTGTAATGGTATTCACATTACTAATAAACTTCTAAATTCATCTGTTTGG

GTATTCATTACATATTCATCAACTAAGCAAGTCTTACATGCGTCCAAATTTGTTTAAGACAAGAAAATAT
ACCATCTATGCTTAATAAACAAACTTCATCTCTTCTAGTACGCAAATAAATACATTAAATTTATTTTACA
TTATAATAAGATTCATCACTTTCTCAAAATTTTGAAACCGGAAACGGAATTCAGCATGTTTTAATAAAAA
GATGAGCAACCGCGTTCTTACTAAATACGCAATTCTGATAATTTGGGAATTGTAGTTAGTAATTTTAAAT
TTAATCATTAAAACATACCAAAAAATGGGCCCGTTCCTTTTAACATTTTGCTCAGACCAAGTGAAAAAAA
CCTAAAACGCGCTCAAGAGGAACAACGGACGAGCTACACTCAAAATATTAATGAATGGGTAGTCAACTAT
TTTACTAGCGAACAAGCAAAAGCTTTGATCCATGGATCTCACGTAATGTAAAATAGTATCTTCCCTAAGC
ATTATTTATTTGAGAAACAGGACGTTCATTATTGTTTGCCGCAAAGAATCCAAGTTCAAGGAAATTGAAT
ACAGACAAGAGTTGCTATTCGTTTGAATGTACATTGTGACCACAAGTCGGTTTTTGTTTCCTTCCAAATA
GTACCTTCAACACGAAGTTGTACATTTAGCACGGACAAAGAATCGACCAAAAAACCAAAATTAAATGTAG
AAGGAAAACCTTCAAGTCATTTTTTTTATAATAGCTCTTTTAAGTTCCATACTTTTTATCTCCGTAACAA
ATGCCAAAAGAAGCTAGTATAGACGAGAGCATTCAGCCAACAAATTTGCACAAGCAAAAATTGTTACCAA
TACAATTTAATATTTGTATGGGGAATTGAAACTATCTATAAAACTTTCTTCATTGCTTGATTTTTAGTAA
CCTTCAGTAATCAAACGAGACTCTTCTACCAAAAATTCGTTGTGATCTGACAAGCACTTAAATGATGGAT
ACAGAATTTATACAAATGAAAGGTAGGATTAAACTGTAAATGAACTTTAAAGGCAGAAGACTCACGTACA
CTACACCCAGCGATTCAATTGATGTCCAGGCCTCTTTTTTCCTACTGTATACAGAAGTATGAAGTCTGCA
GCCATAATAAAAGCGGTAGGAATGATCTATGTTTTCCGCAACGCGTCAACGATTCCATGAAGAGCCTCTC
TTATGAATGTTCCTAATGATCAAAACTCTAAAAAAAGCTAAAAAGAATAATTTCGCAATGCTGAAAAGTT
GAAGAGTACGGTTACACGAAGCAGTACAGGAATTCGAAATCCATCGAAAACAACTATCCAGACAAACTGA
AAAGCTAAACAGACTACTCGTTCTTTCTAGTAGACAGTATACAGGTTTTGGAATAGCAAACTATACTTAA
GGCCTATGAATCATTTCCTTCAAACTTTCAAATCAATCACCAAGTCCTCAAGAACCGTTTGATTAATTTT
CCTTGTCAACCTTCTAAGCATGGGCGTTGCGTTGAGTATAAACAAAACAGCAATAAATCCGAGTATACAT
GAATTGTTTCCTTCTTCAAACAAAACGCTGTTACCTTTGCGCGTATGATGACTTCCCATACATACAAAAA
TTTTCAAAACCTAATA

>10
ACAGATACAATTAAGAATAACTTGTAATGGTATTCACATTACTAATAAACTTCTAAATTCATCTGTTTGG
GTATTCATTACATATTCATCAACTAAGCAAGTCTTACATGCGTCCAAATTTGTTTAAGACAAGAAAATAT
ACCATCTATGCTTAATAAACAAACTTCATCTCTTCTAGTACGCAAATAAATACATTAAATTTATTTTACA
TTATAATAAGATTCATCACTTTCTCAAAATTTTGAAACCGGAAACGGAATTCAGCATGTTTTAATAAAAA
GATGAGCAACCGCGTTCTTACTAAATACGCAATTCTGATAATTTGGGAATTGTAGTTAGTAATTTTAAAT
TTAATCATTAAAACATACCAAAAAATGGGCCCGTTCCTTTTAACATTTTGCTCAGACCAAGTGAAAAAAA
CCTAAAACGCGCTCAAGAGGAACAACGGACGAGCTACACTCAAAATATTAATGAATGGGTAGTCAACTAT
TTTACTAGCGAACAAGCAAAAGCTTTGATCCACGGATCTCACGTAATGTAAAATAGTATCTTCCCTAAGC
ATTATTTTTTTGAGAAACAGGACGTTCATTATTGTTTGCCGCAAAGAATCCAAGTTCAAGGAAATTGAAT
ACAGACCAGAGTTGCTATTCGTTTGAATGTACATTGTGACCACAAGTCGGTTTTTGTTTCCTTCCAAATA
GTACCTTCAACACGAAGTTGTACATTTAGCACGGACAAAGAATCGACCAAAAAACCAAAATTAAATGTAG
AAGGAAAACCTTCAAGTCATTTTTTTTATAATAGCTCTTTTAAGTTCCATACTTTTTATCTCCGTAACAA
ATGCCAAAAGAAGCTAGTATAGACGAGAGCATTCAGCCAACAAATTTGCACAAGCAAAAATTGTTACCAA
TACAATTTAATATTTGTATGGGGAATTGAAACTATCTATAAAACTTTCTTCATTGCTTGATTTTTAGTAA
CGTTCAGTAATCAAACGAGACTCTTCTACCAAAAATTCGTTGTGATCTGACAAGCACTTAAATGATGGAT
ACAGATTTTATACAAATGAAAGGTAGGATTAAACTGTAAATGAATTTTAAAGGCAGAAGACTCACGTACA
CTGCACCCAGCGATTCAATTGATGTCCAGGCCTCTTTTTTCCTACTGTATACAGAAGTATGAAGTCTGCA
GCCATAATAAAAGCGGTAGGAATGATCTATGTTTTCCGCAACGCGTCAACGATTCCATGAAGAGCCTCTC
TTATGAATATTCCTAATGATCAAAACTCTAAAAAAAGCTAAAAAGAATAATTTCGCAATGCTGAAAAGTT
GAAGAGTACGGTTACACGAAGCAGTACAGGAATTCGAAATCCATCGAAAACAACTATCCAGACAAACTGA
AAAGCTAAACAGACTACTCGTTCTTTCTAGTAGACAGTATACAGGTTTTGGAATAGCAAACTATACTTAA
GGCCTATGAATCATTTCCTTCAAACTTTCAAATCAATCACCAAGCCCTCAAGAACCGTTTGATTAATTTT
CCTTGTCAACCTTCTAAGCATGGGCGTTGCGTTGAGTATAAACAAAACAGCAATAAATCCGAGTATACAT
GAATTGTTTCCTTCTTCAAACAAAACGCTGTTACCTTTGCGCGTATGATGACTTCCCATACTTACAAAAA
TTTTCAAAACCTAACA

>11
ACAGATACAATTAAGAATAACTTGTAATGGTATTCACATTACTAATAAACTTCTTAATTCATCTGTTTGG
GTATTCATTACATATTCATCAACTAAGCAAGTCTTACATGCGTCCAAATTTGTTTAAGACAAGAAAATAT
ACCATCTATGCTTAATAAACAAACTTCATCTCTTCTAGTACGCAAATAAATACATTAAATTTATTTTACA
TTATAATAAGATTCATCACTTTCTCAAAATTTTGAAACCGGAAACGGAATTCAGCATGTTTTAATAAAAA
GATGAGCAACCGCGTTCTTACTAAATACGCAATTCTAATAATTTGGGAATTGTAGTTAGTAATTTTAAAT

TTAATCATTAAAACATACCAAAAAATGGGCCCGTTCCTTTTAACATTTTGCTCAGACCAAGTGAAAAAAA
CCTAAAACGCGCTCAAGAGGAACAACGGACGAGCTACACTCAAAATATTAATGAATGGGTAGTCAACTAT
TTTACTAGCGAACAAGCAAAAGCTTTGATCCATGGATCTCACGTAATGTAAAATAGTATCTTCCCTAAGC
ATTATTTATTTGAGAAACAGGACGTTCATTATTGTTTGCCGCAAAGAATCCAAGTTCAAGGAAATTGAAT
ACAGACAAGAGTTGCTATTCGTTTGAATGTACATTGTGACCACAAGTCGGTTTTTGTTTCCTTCCAAATA
GTACCTTCAACACGAAGTTGTACATTTAGCACGGACAAAGAATCGACCAAAAAACCAAAATTAAATGTAG
AAGGAAAACCTTCAAGTCATTTTTTTTATAATAGCTCTTTTAAGTTCCATACTTTTTATCTCCGTAACAA
ATGCCAAAAGAAGCTAGTATAGACGAGAGCATTCAGCCAACAAATTTGCACAAGCAAAAATTGTTACCAA
TACAATTTAATATTTGTATGGGGAATTGAAACTATCTATAAAACTTTCTTCATTGCTTGATTTTTAGTAA
CCTTCAGTAATCAAACGAGACTCTTCTACCAAAAATTCGTTGTGATCTGACAAGCACTTAAATGATGGAT
ACAGAATTTATACAAATGAAAGGTAGGATTAAACTGTAAATGAACTTTAAAGGCAGAAGACTCACGTACA
CTACACCCAGCGATTCAATTGATGTCCAGGCCTCTTTTTTCCTACTGTATACAGAAGTATGAAGTCTGCA
GCCATAATAAAAGCGGTAGGAATGATCTATGTTTTCCGCAACGCGTCAACGATTCCATGAAGAGCCTCTC
TTATGAATGTTCCTAATGATCAAAACTCTAAAAAAAGCTAAAAAGAATAATTTCGCAATGCTGAAAAGTT
GAAGAGTACGGTTACACGAAGCAGTACAGGAATTCGAAATCCATCGAAAACAACTATCCAGACAAACTGA
AAAGCTAAACAGACTACTCGTTCTTTCTAGTAGACAGTATACAGGTTTTGGAATAGCAAACTATACTTAA
GGCCTATGAATCATTTCCTTCAAACTTTCAAATCAATCACCAAGTCCTCAAGAACCGTTTGATTAATTTT
CCTTGTCAACCTTCTAAGCATGGGCGTTGCGTTGAGTATAAACAAAACAGCAATAAATCCGAGTATACAT
GAATTGTTTCCTTCTTCAAACAAAACGCTGTTACCTTTGCGCGTATGATGACTTCCCATACTTACAAAAA
TTTTCAAAACCTAATA

>12
ACAGATACAATTAAGAATAACTTGTAATGGTATTCACATTACTAATAAACTTCTAAATTCATCTGTTTGG
GTATTCATTACATATTCATCAACTAAGCAAGTCTTACATGCGTCCAAATTTGTTTAAGACAAGAAAATAT
ACCATCTATGCTTAATAAACAAACTTCATCTCTTCTAGTACGCAAATAAATACATTAAATTTATTTTACA
TTATAATAAGATTCATCACTTTCTCAAAATTTTGAAACCGGAAACGGAATTCAGCATGTTTTAATAAAAA
GATGAGCAACCGCGTTCTTACTAAATACGCAATTCTAATAATTTGGGAATTGTAGTTAGTAATTTTAAAT
TTAATCATTAAAACATACCAAAAAATGGGCCCGTTCCTTTTAACATTTTGCTCAGACCAAGTGAAAAAAA
CCTAAAACGCGCTCAAGAGGAACAACGGACGAGCTACACTCAAAATATTAATGAATGGGTAGTCAACTAT
TTTACTAGCGAACAAGCAAAAGCTTTGATCCATGGATCTCACGTAATGTAAAATAGTATCTTCCCTAAGC
ATTATTTATTTGAGAAACAGGACGTTCATTATTGTTTGCCGCAAAGAATCCAAGTTCAAGGAAATTGAAT
ACAGACAAGAGTTGCTATTCGTTTGAATGTACATTGTGACCACAAGTCGGTTTTTGTTTCCTTCCAAATA
GTACCTTCAACACGAAGTTGTACATTTAGCACGGACAAAGAATCGACCAAAAAACCAAAATTAAATGTAG
AAGGAAAACCTTCTAGTCATTTTTTTTATAATAGCTCTTTTAAGTTCCATACTTTTTATCTCCGTAACAA
ATGCCAAAAGAAGCTAGTATAGACGAGAGCATTCAGCCAACAAATTTGCACAAGCAAAAATTGTTACCAA
TACAATTTAATATTTGTATGGGGAATTGAAACTATCTATAAAACTTTCTTCATTGCTTGATTTTTAGTAA
CCTTCAGTAATCAAACGAGACTCTTCTACCAAAAATTCGTTGTGATCTGACAAGCACTTAAATGATGGAT
ACAGAATTTATACAAATGAAAGGTAGGATTAAACTGTAAATGAACTTTAAAGGCAGAAGACTCACGTACA
CTACACCCAGCGATTCAATTGATGTCCAGGCCTCTTTTTTCCTACTGTATACAGAAGTATGAAGTCTGCA
GCCATAATAAAAGCGGTAGGAATGATCTATGTTTTCCGCAACGCGTCAACGATTCCATGAAGAGCCTCTC
TTATGAATGTTCCTAATGATCAAAACTCTAAAAAAAGCTAAAAAGAATAATTTCGCAATGCTGAAAAGTT
GAAGAGTACGGTTACACGAAGCAGTACAGGAATTCGAAATCCATCGAAAACAACTATCCAGACAAACTGA
AAAGCTAAACAGACTACTCGTTCTTTCTAGTAGACAGTATACAGGTTTTGGAATAGCAAACTATACTTAA
GGCCTATGAATCATTTCCTTCAAACTTTCAAATCAATCACCAAGTCCTCAAGAACCGTTTGATTAATTTT
CCTTGTCAACCTTCTAAGCATGGGCGTTGCGTTGAGTATAAACAAAACAGCAATAAATCCGAGTATACAT
GAATTGTTTCCTTCTTCAAACAAAACGCTGTTACCTTTGCGCGTATGATGACTTCCCATACATACAAAAA
TTTTCAAAACCTAATA

>13
ACAGATACAATTAAGAATAACTTGTAATGGTATTCACATTACTAATAAACTTCTTAATTCATCTGTTTGG
GTATTCATTACATATTCATCAACTAAGCAAGTCTTACATGCGTCCAAATTTGTTTAAGACAAGAAAATAT
ACCATCTATGCTTAATAAACAAACTTCATCTCTTCTAGTACGCAAATAAATACATTAAATTTATTTTACA
TTATAATAAGATTCATCACTTTCTCAAAATTTTGAAACCGGAAACGGAATTCAGCATGTTTTAATAAAAA
GATGAGCAACCGCGTTCTTACTAAATACGCAATTCTAATAATTTGCGAATTGTAGTTAGTAATTTTAAAT
TTAATCATTAAAACATACCAAAAAATGGGCCCGTTCCTTTTAACATTTTGCTCAGACCAAGTGAAAAAAA
CCTAAAACGCGCTCAAGAGGAACAACGGACGAGCTACACTCAAAATATTAATGAATGGGTAGTCAACTAT
TTTACTAGCGAACAAGCAAAAGCTTTGATCCATGGATCTCACGTAATGTAAAATAGTATCTTTCCTAAGC
ATTATTTATTTGAGAAACAGGACGTTCATTATTGTTTGCCGCAAAGAATCCAAGTTCAAGGAAATTGAAT

ACAGACAAGAGTTGCTATTCGTTTGAATGTACATTGTGACCACAAGTCGGTTTTTGTTTCCTTCCAAATA
GTACCTTCAACACGAAGTTGTACATTTAGCACGGACAAAGAATCGACCAAAAAACCAAAATTAAATGTAG
AAGGAAAACCTTCAAGTCATTTTTTTTATAATAGCTCTTTTAAGTTCCATACTTTTTATCTCCGTAACAA
ATGCCAAAAGAAGCTAGTATAGACGAGAGCATTCAGCCAACAAATTTGCACAAGCAAAAATTGTTACCAA
TACAATTTAATATTTGTATGGGGAATTGAAACTATCTATAAAACTTTCTTCATTGCTTGATTTTTAGTAA
CCTTCAGTAATCAAACGAGACTCTTCTACCAAAAATTCGTTGTGATCTGACAAGCACTTAAATGATGGAT
ACAGAATTTATACAAATGAAAGGTAGGATTAAACTGTAAATGAACTTTAAAGGCAGAAGACTCACGTACA
CTACACCCAGCGATTCAATTGATGTCCAGGCCTCTTTTTTCCTACTGTATACAGAAGTATGAAGTCTGCA
GCCATAATAAAAGCGGTAGGAATGATCTATGTTTTCCGCAACGCGTCAACGATTCCATGAAGAGCCTCTC
TTATGAATGTTCCTAATGATCAAAACTCTAAAAAAAGCTAAAAAGAATAATTTCGCAATGCTGAAAAGTT
GAAGAGTACGGTTACACGAAGCAGTACAGGAATTCGAAATCCATCGAAAACAACTATCCAGACAAACTGA
AAAGCTAAACAGACTACTCGTTCTTTCTAGTAGACAGTATACAGGTTTTGGAATAGCAAACTATACTTAA
GGCCTATGAATCATTTCCTTCAAACTTTCAAATCAATCACCAAGTCCTCAAGAACCGTTTGATTAATTTT
CCTTGTCAACCTTCTAAGCATGGGCGTTGCGTTGAGTATAAACAAAACAGCAATAAATCCGAGTATACAT
GAATTGTTTCCTTCTTCAAACAAAACGCTGTTACCTTTGCGCGTATGATGACTTCCCATACTTACAAAAA
TTTTCAAAACCTAATA

>14
ACAGATACAATTAAGAATAACTTGTAATGGTATTCACATTACTAATAAACTTCTAAATTCATCTGTTTGG
GTATTCATTACATATTCATCAACTAAGCAAGTCTTACATGCGTCCAAATTTGTTTAAGACAAGAAAATAT
ACCATCTATGCTTAATAAACAAACTTCATCTCTTCTAGTACGCAAATAAATACATTAAATTTATTTTACA
TTATAATAAGATTCATCACTTTCTCAAAATTTTGAAACCGGAAACGGAATTCAGCATGTTTTAATAAAAA
GATGAGCAACCGCGTTCTTACTAAATACGCAATTCTGATAATTTGGGAATTGTAGTTAGTAATTTTAAAT
TTAATCATTAAAACATACCAAAAAATGGGCCCGTTCCTTTTAACATTTTGCTCAGACCAAGTGAAAAAAA
CCTAAAACGCGCTCAAGAGGAACAACGGACGAGCTACACTCAAAATATTAATGAATGGGTAGTCAACTAT
TTTACTAGCGAACAAGCAAAAGCTTTGATCCATGGATCTCACGTAATGTAAAATAGTATCTTCCCTAAGC
ATTATTTTTTTGAGAAACAGGACGTTCATTATTGTTTGCCGCAAAGAATCCAAGTTCAAGGAAATTGAAT
ACAGACCAGAGTTGCTATTCGTTTGAATGTACATTGTGACCACAAGTCGGTTTTTGTTTCCTTCCAAATA
GTACCTTCAACACGAAGTTGTACATTTAGCACGGACAAAGAATCGACCAAAAAAACAAAATTAAATGTAG
AAGGAAAACCTTCAAGTCATTTTTTTTATAATAGCTCTTTTAAGTTCCATACTTTTTATCTCCGTAACAA
ATGCCAAAAGAAGCTAGTATAGACGAGAGCATTCAGCCAACAAATTTGCACAAGCAAAAATTGTTACCAA
TACAATTTAATATTTGTATGGGGAATTGAAACTATCTATAAAACTTTCTTCATTGCTTGATTTTTAGTAA
CGTTCAGTAATCAAACGAGACTCTTCTACCAAAAATTCGTTGTGATCTGACAAGCACTTAAATGATGGAT
ACAGATTTTATACAAATGAAAGGTAGGATTAAACTGTAAATGAATTTTAAAGGCAGAAGACTCACGTACA
CTGCACCCAGCGATTCAATTGATGTCCAGGCCTCTTTTTTCCTACTGTATACAGAAGTATGAAGTCTGCA
GCCATAATAAAAGCGGTAGGAATGATCTATGTTTTCCGCAACGCGTCAACGATTCCATGAAGAGCCTCTC
TTATGAATATTCCTAATGATCAAAACTCTAAAAAAAGCTAAAAAGAATAATTTCGCAATGCTGAAAAGTT
GAAGAGTACGGTTACACGAAGCAGTACAGGAATTCGAAATCCATCGAAAACAACTATCCAGACAAACTGA
AAAGCTAAACAGACTACTCGTTCTTTCTAGTAGACAGTATACAGGTTTTGGAATAGCAAACTATACTTAA
GGCCTATGAATCATTTCCTTCAAACTTTCAAATCAATCACCAAGCCCTCAAGAACCGTTTGATTAATTTT
CCTTGTCAACCTTCTAAGCATGGGCGTTGCGTTGAGTATAAACAAAACAGCAATAAATCCGAGTATACAT
GAATTGTTTCCTTCTTCAAACAAAACGCTGTTACCTTTGCGCGTATGATGACTTCCCATACTTACAAAAA
TTTTCAAAACCTAACA

TER1 + 30

>1
TTATTGAGAAAGTGGATTCAATCAGCTATATGTTAAGCATTAGTAATTGAAGAGGTCTCTCATTTTACTTT
AATCGAAGCTGCTATGGAATTTCGACTTTATGGCTGATTAGAAGCGTGTACTGTATACTCACCTCGTTTAA
CATTTCTTTGACTCGAATTCGCAATTGGTCTTCACGACCCATTCCAGCTTCCCGTGAAGCTTGTTCTGGTA
AATGAATAGCAAGTTTTTCTTGTATGGCTTGACGAAACACCGATTTTACGTAACTGTAATCGTTGATACTT
ATAAAGTGTTAAAATCAGATTTTCTAATTTCAAATTAAAAGCATAAATAAAAGCTCTAAACTACATGGGAT
TATGAAACACACCTTTCCAATGCAATTTTTGGTAAAACGCTAGCATCTCCGTGAGACGACATGAATGCTAG
AAAGAAATGTTTTTCTTTAAGAATATATGATTAATAGATTAGAATATATTAAGAGAAAGTTAAATTTTTAG
CTGAGAGATAGAAATCATTAGTCTGAAAAGAATTTGGATGTGATAGTA
>2
TTATTGAGAAAGTGGATTCAATCAGCTATATGTTAAGCATTAGTAATTGAAGAGGTCTCT
CATTTTACTTTAATCGAAGCTGCTATGGAATTTCGACTTTATGGCTGATTAGAAGCGTGT

ACTGTATACTCACCTCGTTTAACATTTCTTTGACTCGAATTCGCAATTGGTCTTCACGAC
CCATTCCAGCTTCCCGTGAAGCTTGTTCTGGTAAATGAATAGCAAGTTTTTCTTGTATGG
CTTGGCGAAACACCGATTTTACGTAACTGTAATCGTTGATACTTAGAAAGTGTTAAAATC
AGATTTTCTAATTTCAAATTAAAAGCATAAATAAAAGCTCTAAACTACAATGGGATTATG
AAACACACCTTTCCAATGCAATTTTTGGTAAAACGCTAGCATCTCCGTGAGACGACATGA
ATGCTAGAAAGAAATGATTTTCTTTAAGAATATATGATTAATAGATTAGAATATATTAAG
AGAAAGTTAAATTTTTAGCTGAGAGATAGAAATCATTAGTCTGAAAAGAATTTGGATGTG
ATAGTA
>3
TTATTGAGAAAGTGGATTCAATCAGCTATATGTTAAGCATTAGTAATTGGAGAGGTCTCT
CATTTTACTTTAATCGAAGCTGCTATGGAATTTCGACTTTATGGCTGATTAGAAGCGTGT
ACTGTATACTCACCTCGTTTAACATTTCTTTGACTCGAATTCGCAATTGGTCTTCACGAC
CCATTCCAGCTTCCCGTGAAGCTTGTTCTGGTAAATGAATAGCAAGTTTTTCTTGTATGG
CTTGACGAAACACCGATTTTACGTAACTGTAATCGTTGATACTTATAAAGTGTTAAAATC
AGATTTTCTAATTTCAAATTAAAAGCATAAATAAAAGCTCTAAACTACA-TGGGATTATG
AAACACACCTTTCCAATGCAATTTTTGGTAAAACGCTAGCATCTCCGTGAGACGACATGA
ATGCTAGAAAGAAATGTTTTTCTTTAAGAATATATGATTAATAGATTAGAATATATTAAG
AGAAAGTTAAATTTTTAGCTGAGAGATAGAAATCATTAGTCTGAAAAGAATTTGGATGTG
ATAGTA
>4
TTATTGAGAAAGTGGATTCAATCAGCTATATGTTAAGCATTAGTAATTGAAGAGGTCTCT
CATTTTACTTTAATCGAAGCTGCTATGGAATTTCGACTTTATGGCTGATTAGAAGCGTGT
ACTGTATACTCACCTCGTTTAACATTTCTTTGACTCGAATTCGCAATTGGTCTTCACGAC
CCATTCCAGCTTCCCGTGAAGCTTGTTCTGGTAAATGAATAGCAAGTTTTTCTTGTATGG
CTTGACGAAACACCGATTTTACGTAACTGTAATCGTTGATACTTATAAAGTGTTAAAATC
AGATTTTCTAATTTCAAATTAAAAGCATAAATAAAAGCTCTAAACTACAATGGGATTATG
AAACACACCTTTCCAATGCAATTTTTGGTAAAACGCTAGCATCTCCGTGAGACGACATGA
ATGCTAGAAAGAAATGATTTTCTTTAAGAATATATGATTAATAGATTAGAATATATTAAG
AGAAAGTTAAATTTTTAGCTGAGAGATAGAAATCATTAGTCTGAAAAGAATTTGGATGTG
ATAGTA
>5
TTATTGAGAAAGTGGATTCAATCAGCTATATGTTAAGCATTAGTAATTGAAGAGGTCTCT
CGTTTTACTTTAATCGAAGCTGCTATGGAATTTCGACTTTATGGCTGATTAGAAGCGTGT
ACTGTATACTCACCTCGTTTAACATTTCTTTGACTCGAATTCGCAATTGGTCTTCACGAC
CCATTCCAGCTTCCCGTGAAGCTTGTTCTGGTAAATGAATAGCAAGTTTTTCTTGTATGG
CATGGCGAAACACCGATTTTACGTAACTGTAATCGTTGATACTTAGAAAGTGTTAAAATC
AGATTTTCTAATTTCAAATTAAAAGCATAAATAAAAGCTCTAAACTACAATGGGATTATG
AAACACACCTTTCCAATGCAATTTTTGGTAAAACGCTAGCATCTCCGTGAGACGACATGA
ATGCTAGAAAGAAATGATTTTCTTTAAGAATATATGATTAATAGATTAGAATATATTAAG
AGAAAGTTAAATTTTTAGCTGAGAGATAGAAATCATTAGTCTGAAAAGAATTTGGATGTG
ATAGTA
>6
TTATTGAGAAAGTGGATTCAATCAGCTATATGTTAAGCATTAGTAATTGAAGAGGTCTCT
CATTTTACTTTAATCGAAGCTGCTATGGAATTTCGACTTTATGGCTGATTAGAAGCGTGT
ACTGTATACTCACCTCGTTTAACATTTCTTTGACTCGAATTCGCAATTGGTCTTCACGAC
CCATTCCAGCTTCCCGTGAAGCTTGTTCTGGTAAATGAATAGCAAGTTTTTCTTGTATGG
CTTGACGAAACACCGATTTTACGTAACTGTAATCGTTGATACTTATAAAGTGTTAAAATC
AGATTTTCTAATTTCAAATTAAAAGCATAAATAAAAGCTCTAAACTACAATGGGATTATG
AAACACACCTTTCCAATGCAATTTTTGGTAAAACGCTAGCATCTCCGTGAGACGACATGA
ATGCTAGAAAGAAATGATTTTCTTTAAGAATATATGATTAATAGATTAGAATATATTAAG
AGAAAATTAAATTTTTAGCTGAGAGATAGAAATCATTAGTCTGAAAAGAATTTGGATGTG
ATAGTA

TER1 + 100

>1
ATCAAGAAACGTTCCCGCTACTGTGCAAGTTGCGCTTGATGAATACGCTCAAAATCCCTCTACTGCATCT
GAGACACTAGTAAACAAGGAGCTTGCTAATTTTTCATCTATAAAGGAAGCTGTTTCGAAAACTCTAGAAC

TGCGGGAAAAAGTGCGGGCCCTTGAATGCGATGTCGAAATCCAAAAGCAAACTGTCCAATATCAAATTTC
CAATGCAGTTAAAGAAAATTCAAATACACTTTCAGAGCAAATGAAAAATTTGGAAAGTGAGTTAAATTCG
TCAAAAATAAAAAATGAGTCCTTACTTAATGAACGAAATTTACTGAAAGAAATGTTAGCTACTTCTCGAA
GCAGTATTTTGTCTCACAATTCTTCCGCGGGCAATATTGATGACAAAATGAAATCCATTGATGAATCTAC
AAGAGAACTTGAGAAAAATTACGAAGTTTATCGTAACGAAATGACAGCTATTCAGGAATCCTTGTCTAAG
CGAAATCAGGATTTGTTGAGTGAGATGGAAGCGATACGTAAAGAGTTAGAAAATTCCAAATATCAGCAGC
AGTTATCTACTGATAGATTAACTAATGCTAATAATGACGTTGAAGCCTT

>2
ATCAAGAAACGTTCCCGCTACTGTGCAAGTTGCGCTTGATGAATACGCTCAAAATCCCTCTACTGCATCT
GAGACACTAGTAAACAAGGAGCTTGCTAATTTTTCATCTATAAAGGAAGCTGTTTCGAAAACTCTAGAAC
TGCGGGAAAAAGTGCGGGCCCTTGAATGCGATGTTGAAATCCAAAAGCAAACTGTCCAATATCAAATTTC
CAATGCAGTTAAAGAAAATTCAAATACACTTTCAGAGCAAATCAAAAATTTGGAAAGTGAGTTAAATTCG
TCAAAAATAAAAAATGAGTCCTTACTTAATGAACGAAATTTACTGAAAGAAATGTTAGCTACTTCTCGAA
GCAGTATTTTGTCTCACAATTCTTCCGCGGGCAATATTGATGACAAAATGAAATCCATTGATGAATCTAC
AAGAGAACTTGAGAAAAATTACGAAGTTTATCGTAACGAAATGACAGCTATTCAGGAATCCTTGTCTAAG
CGAAATCAGGATTTGTTGAGTGAGATGGAAGCAATACGTAAAGAGTTAGAAAATTCCAAATATCAGCAGC
AGTTATCTACTGATAGATTAACTAATGCTAATAATGACGTTGAAGCCTT

>3
ATCAAGAAACGTTCCCGCTACTGTGCAAGTTGCGCTTGATGAATACGCTCAAAATCCCTCTACTGCATCT
GAGACACTAGTAAACAAGGAGCTTGCTAATTTTTCATCTATAAAGGAAGCTGTTTCGAAAACTCTAGAAC
TGCGGGAAAAAGTGCGGGCCCTTGAATGCGATGTCGAAATCCAAAAGCAAACTGTCCAATATCAAATTTC
CAATGCAGTTAAAGAAAATTCAAATACACTTTCAGAGCAAATCAAAAATTTGGAAAGTGAGTTAAATTCG
TCAAAAATAAAAAATGAGTCCTTACTTAATGAACGAAATTTACTGAAAGAAATGTTAGCTACTTCTCGAA
GCAGTATTTTGTCTCACAATTCTTCCGCGGGCAATATTGATGACAAAATGAAATCCATTGATGAATCTAC
AAGAGAACTTGAGAAAAATTACGAAGTTTATCGTAACGAAATGACAGCTATTCAGGAGTCCTTGTCTAAG
CGAAATCAGGATTTGTTGAGTGAGATGGAAGCGATACGTAAAGAGTTAGAAAATTCCAAATATCAGCAGC
AGTTATCTACTGATAGATTAACTAATGCTAATAATGACGTTGAAGCCTT

>4
ATCAAGAAACGTTCCCGCTACTGTGCAAGTTGCGCTTGATGAATACGCTCAAAATCCCTCTACTGCATCT
GAGACACTAGTAAACAAGGAGCTTGCTAATTTTTCATCTATAAAGGAAGCTGTTTCGAAAACTCTAGAAC
TGCGGGAAAAAGTGCGGGCCCTTGAATGCGATGTTGAAATCCAAAAGCAAACTGTCCAATATCAAATTTC
CAATGCAGTTAAAGAAAATTCAAATACACTTTCAGAGCAAATCAAAAATTTGGAAAGTGAGTTAAATTCG
TCAAAAATAAAAAATGAGTCCTTACTTAATGAACGAAATTTACTGAAAGAAATGTTAGCTACTTCTCGAA
GCAGTATTTTGTCTCACAATTCTTCCGCGGGCAATATTGATGACAAAATGAAATCCATTGATGAATCTAC
AAGAGAACTTGAGAAAAATTACGAAGTTTATCGTAACGAAATGACAGCTATTCAGGAATCCTTGTCTAAG
CGAAATCAGGATTTGTTGAGTGAGATGGAAGCGATACGTAAAGAGTTAGAAAATTCCAAATATCAGCAGC
AGTTATCTACTGATAGATTAACTAATGCTAATAATGACGTTGAAGCCTT

>5
ATCAAGAAACGTTCCCGCTACTGTGCAAGTTGCGCTTGATGAATACGCTCAAAATCCCTCTACTGCATCT
GAGACACTAGTAAACAAGGAGCTTGCTAATTTTTCATCTATAAAGGAAGCTGTTTCGAAAACTCTAGAAC
TGCGGGAAAAAGTGCGGGCCCTTGAATGCGATGTCGAAATCCAAAAGCAAACTGTCCAATATCAAATTTC
CAATGCAGTTAAAGAAAATTCAAATACACTTTCAGAGCAAATGAAAAATTTGGAAAGTGAGTTAAATTCG
TCAAAAATAAAAAATGAGTCCTTACTTAATGAACGAAATTTACTGAAAGAAATGTTAGCTACTTCTCGAA
GCAGTATTTTGTCTCACAATTCTTCCGCGGGCAATATTGATGACAAAATGAAATCCATTGATGAATCTAC
AAGAGAACTTGAGAAAAATTACGAAGTTTATCGTAACGAAATGACAGCTATTCAGGAATCCTTGTCTAAG
CGAAATCAGGATTTGTTGAGTGAGATGGAAGCGATACGTAAAGAGTTAGAAAATTCCAAATATCAGCAGC
AGTTATCTACTGATAGATTAGCTAATGCTAATAATGACGTTGAAGCCTT

CC2L

>1
CAGCACGGACGGTAAGCTCGCGATGGGCGCGATGCATGGGATTACGGGTTTGGAAAGCAACGACGCGATT
CCAGTTGTTACGCTGGAAGTCAGAGCGAAGTTGAGCAGGTGAGTAGCGGTACTCTACGAAATCGAAATGG
CGAATAGGAGTAACGGCTTGAAGTTTACCACCAACATACACATTTCCAGCACGACCAAACAAGTAGTCGA

CGGCAGGGTGGGCGCGATCATTAGCACCAAATACCTTTTCAGCTTCGTTCGCCTTATCTGGGGTATACTT
GTCCTCAACAGTCAAAATGGCAATAACAGTTTGCCCATCCCTAGGATCTAATAATGCAACACGATCACCG
GCCTTCAAAGAATCGGCCTGCGATTCATTGAGGTCCAAGGTGATAGGGATGGGAAAGACCTCGCCAGTGC
TGAGACGCAAATTCTCGACGACATTCAAATAATCCTTTTGGTTCATGAATCCGTCTAATGGACTGAAACC
TCCGTTCAAAATGAGTTCCACATCGCAAAACTGACGTTCCGATAGTACAATTTTGGGAAGGCTAGTAGCC
TCCTGTTCCAGTTGTTCTCTCAGAGGAGCATCTCTGGCGTTAAGATCTTTCAACAAAGCTTTAGTCATTT
TGACACCCCAAATCGTAAAGGACCTGCTT

>2
CAGCACGGACGGTAAGCTCACGATGGGCGCGATGCATGGGATTACGGGTTTGGAAAGCAACGACGCGATT
CCAGTTGTTACGCTGGAAGTCAGAGCGAAGTTGAGCAGGTGAGTAGCGGTACTCTACGAAATCGAAATGG
CGAATAGGAGTAACGGCTTGAAGTTTACCACCAACATACACATTTCCAGCACGACCAAACAAGTAGTCGA
CGGCAGGGTGGGCGCGATCATTAGCACCAAATACCTTTTCAGCTTCGTTCGCCTTATCTGGGGTATACTT
GTCCTCAACAGTCAAAATGGCAATAACAGTTTGCCCATCCCTAGGATCTAATAATGCAACACGATCACCG
GCCTTCAAAGAATCGGCCTGCGATTCATTGAGGTCCAAGGTGATAGGGATGGGAAAGACCTCGCCAGTGC
TGAGACGCAAATTCTCGACGACATTCAAATAATCCTTTTGGTTCATGAATCCGTCTAATGGACTGAAACC
TCCGTTCAAAATGAGTTCCACATCGCAAAACTGACGTTCCGATAGTACAATTTTGGGAAGGCTAGTAGCC
TCCTGTTCCAGTTGTTCTCTCAGAGGAGCATCTCTGGCGTTAAGATCTTTCAACAAAGCTTTAGTCATTT
TGACACCCCAAATCGTAAAGGACCTGCTT

>3
CAGCACGGACGGTAAGCTCACGATGGGCGCGATGCATGGGATTACGGGTTTGGAAAGCAACGACGCGATT
CCAGTTGTTACGCTGGAAGTCAGAGCGAAGTTGAGCAGGTGAGTAGCGGTACTCTACGAAATCGAAATGG
CGAATAGGAGTAACGGCTTGAAGTTTACCACCAACATACACATTTCCAGCACGATCAAACAAGTAGTCGA
CGGCAGGGTGGGCGCGATCATTAGCACCAAATACCTTTTCAGCTTCGTTCGCCTTATCTGGGGTATACTT
GTCCTCAACAGTCAAAATGGCAATAACAGTTTGCCCATCCCTAGGATCTAATAATGCAACACGATCACCG
GCCTTCAAAGAATCGGCCTGCGATTCATTGAGGTCCAAGGTGATAGGGATGGGAAAGACCTCGCCAGTGC
TGAGACGCAAATTCTCGACGACATTCAAATAATCCTTTTGGTTCATGAATCCGTCTAATGGACTGAAACC
TCCGTTCAAAATGAGTTCCACATCGCAAAACTGACGTTCCGATAGTACAATTTTGGGAAGGCTAGTAGCC
TCCTGTTCCAGTTGTTCTCTCAGAGGAGCATCTCTGGCGTTAAGATCTTTCAACAAAGCTTTAGTCATTT
TGACACCCCAAATCGTAAAGGACCTGCTT

>4
CAGCACGGACGGTAAGCTCACGATGGGCGCGATGCATGGGATTACGGGTTTGGAAAGCAACGACGCGATT
CCAGTTGTTACGCTGGAAGTCAGAGCGAAGTTGAGCAGGTGAGTAGCGGTACTCTACGAAATCGAAATGG
CGAATAGGAGTAACGGCTTGAAGTTTACCACCAACATACACATTTCCAGCACGACCAAACAAGTAGTCGA
CGGCAGGGTGGGCGCGATCATTAGCACCAAATACCTTTTCAGCTTCGTTCGCCTTATCTGGGGTATACTT
GTCCTCAACAGTCAAAATGGCAATAACAGTTTGCCCATCCCTAGGATCTAATAATGCAACACGATCACCG
GCCTTCAAAGAATCGGCCTGCGATTCATTGAGGTCCAAGGTGATAGGGATGGGAAAGACCTCGCCAGTGC
TGAGACGCAAATTCTCGACGACATTCAAATAATCCTTTTGGTTCATGAATCCGTCTAATGGACTGAAACC
TCCGTTCAAAATGAGTTCCACATCGCAAAACTGACGTTCCGATAGTACAATTTTGGGAAGGCTAGTAGCC
TCCTGTTCCAGTTGTTCTCTCAGAGGAGCATCTCTGGCGTTAAGATCTTTCAACAAAGCTTTAGTCATTT
TGACACCCCAAATCGTAAAGGACCTGGTT

>5
CAGCACGGACGGTAAGCTCACGATGGGCGCGATGCATGGGATTACGGGTTTGGAAAGCAACGACGCGATT
CCAGTTGTTACGCTGGAAGTCAGAGCGAAGTTGAGCAGGTGAGTAGCGGTACTCTACGAAATCGAAATGG
CGAATAGGAGTAACGGCTTGAAGTTTACCACCAACATACACATTTCCAGCACGACCAAACAAGTAGTCGA
CGGCAGGGTGGGCGCGATCATTAGCACCAAATACCTTTTCAGCTTCGTTCGCCTTATCTGGGGTATACTT
GTCCTCAACAGTCAAAATGGCAATAACAGTTTGCCCATCCCTAGGATCTAATAATGCAACACGATCACCG
GCCTTCAAAGAATCGGCCTGCGATTCATTGAGGTCCAAGGTGATAGGGATGGGAAAGACCTCGCCAGTGC
TGAGACGCAAATTCTCGACGACATTCAAGTAATCCTTTTGGTTCATGAATCCGTCTAATGGACTGAAACC
TCCGTTCAAAATGAGTTCCACATCGCAAAACTGACGTTCCGATAGTACAATTTTGGGAAGGCTAGTAGCC
TCCTGTTCCAGTTGTTCTCTCAGAGGAGCATCTCTGGCGTTAAGATCTTTCAACAAAGCTTTAGTCATTT
TGACACCCCAAATCGTAAAGGACCTGCTT

CC2R

>1
GTGCCTTTATATTCTTTAACAAGGCTATATGTTTTAGCTTTGTAAGAAGGATGGCGATCTCGTACAGCGG
TACGATGTCCCAAGACACCACCCAAGACACCAATGAGAACACTACAAATTAAAGCGACTATAAAAATTAA
TATCCAAATAAAGTAACGTCGGTACCAAGGCAATAACTTTTTCTCTGCAGCCTTTTCGTTAAAGGCTTCA
TTGTTTTCGTTATTATTATTGCTATCAAGGTTTTCATGGTCATTATCGTGATCATCAATTTCAGTGTCTG
TATGATTGTCGATAACATCATTAGTTGACTCGTTCTTGCGTGCCGGGTCGTAAATATTCTCAAAGCTAGC
AGAGGACAATGCAGCAGATGAGAGACTATGGCGTGAATCTGTAGTGGAATCAGGAATACCCATTGCAAAA
GTAGTAAAAGTTAATCAAATTTCAACAAATTAAAGAAGGGCGAGAAAAATCAAAAAGAAAGAAAAGATAA
GAATAAAAAGAGCGGGAAGTAATGAGACACGATCAAATAAAAAAATGCCAATCTGGTGAGCTATGCACCA
AGTTCAGAAATGGAAATAAAATGCTAGTAAAAATAACCAAACTCAAGGATATCTTGTTAAAGGGGAATAA
AAAAGGGAAATATGGGATAATGCTTTGTAGAGATGAGAAATGCGGTTTAAAATGAAAATAATTATTATCG
AAAGACGATAGATAGGGAG

>2
GTGCCTTTATATTCTTTAACAAGGCTATATGTTTTAGCTTTGTAAGAAGGATGGCGATCTCGTACAGCGG
TACGATGTCCCAAGACACCACCCAAGACACCAATGAGAACACTACAAATTAAAGCGACTATAAAAATTAA
TATCCAAATAAAGTAACGTCGGTACCAAGGCAATAACTTTTTCTCTGCAGCCTTTTCGTTAAAGGCTTCA
TTGTTTTCGTTATTATTATTGCTATCAAGGTTTTCATGGTCATTATCGTGATCATCAATTTCAGTGTCTG
TATGATTGTCGATAACATCATTAGTTGACTCGTTCTTGCGTGCCGGGTCGTAAATATTCTCAAAGCTAGC

AGAGGACAATGCAGCAGATGAGAGACTATGGCGTGAATCTGTCGTGGAATCAGGAATACCCATTGCAAAA
GTAGTAAAAGTTAATCAAATTTCAACAAATTAAAGAAGGGCGAGAAAAATCAAAAAGAAAGAAAAGATAA
GAATAAAAAGAGCGGGAAGTAATGAGACACGATCAAATAAAAAAATGCCAATCTGGTGAGCTATGCACCA
AGTTCAGAAATGGAAATAAAATGCTAGTAAAAATAACCAAACTCAAGGATATCTTGTTAAAGGGGAATAA
AAAAGGGAAATATGGGATAATGCTTTGTAGAGATGAGAAATGCGGTTTAAAATGAAAATAATTATTATCG
AAAGACGATAGATAGGGAG

>3
GTGCCTTTATATTCTTTAACAAGGCTATATGTTTTAGCTTTGTAAGAAGGATGGCGATCTCGTACAGCGG
TACGATGTCCCAAGACACCACCCAAGACACCAATGAGAACACTACAAATTAAAGCGACTATAAAAATTAA
TATCCAAATAAAGTAACGTCGGTACCAAGGCAATAACTTTTTCTCTGCAGCCTTTTCGTTAAAGGCTTCA
TTGTTTTCGTTATTATTATTGCTATCAAGGTTTTCATGGTCATTATCGTGATCATCAATTTCAGTGTCTG
TATGATTGTCGATAACATCATTAGTTGACTCGTTCTTGCGTGCCGGGTCGTAAATATTCTCAAAGCTAGC
AGAGGACAACGCAGCAGATGAGAGACTATGGCGTGAATCTGTAGTGGAATCAGGAATACCCATTGCAAAA
GTAGTAAAAGTTAATCAAATTTCAACAAATTAAAGAAGGGCGAGAAAAATCAAAAAGAAAGAAAAGATAA
GAATAAAAAGAGCGGGAAGTAATGAGACACGATCAAATAAAAAAATGCCAATCTGGTGAGCTATGCACCA
AGTTCAGAAATGGAAATAAAATGCTAGTAAAAATAACCAAACTCAAGGATATCTTGTTAAAGGGGAATAA
AAAAGGGAAATATGGGATAATGCTTTGTAGAGATGAGAAATGCGGTTTAAAATGAAAATAATTATTATCG
AAAGACGATAGATAGGGAG

>4
GTGCCCTTATATTCTTTAACAAGGCTATATGTTTTAGCTTTGTAAGAAGGATGGCGATCTCGTACAGCGG
TACGATGTCCCAAGACACCACCCAAGACACCAATGAGAACACTACAAATTAAAGCGACTATAAAAATTAA
TATCCAAATAAAGTAACGTCGGTACCAAGGCAATAACTTTTTCTCTGCAGCCTTTTCGTTAAAGGCTTCA
TTGTTTTCGTTATTATTATTGCTATCAAGGTTTTCATGGTCATTATCGTGATCATCAATTTCAGTGTCTG
TATGATTGTCGATAACATCATTAGTTGACTCGTTCTTGCGTGCCGGGTCGTAAATATTCTCAAAGCTAGC
AGAGGACAATGCAGCAGATGAGAGACTATGGCGTGAATCTGTAGTGGAATCAGGAATACCCATTGCAAAA
GTAGTAAAAGTTAATCAAATTTCAACAAATTAAAGAAGGGCGAGAAAAATCAAAAAGAAAGAAAAGATAA
GAATAAAAAGAGCGGGAAGTAATGAGACACGATCAAATAAAAAAATGCCAATCTGGTGAGCTATGCACCA
AGTTCAGAAATGGAAATAAAATGCTAGTAAAAATAACCAAACTCAAGGATATCTTGTTAAAGGGGAATAA
AAAAGGGAAATATGGGATAATGCTTTGTAGAGATGAGAAATGCGGTTTAAAATGAAAATAATTATTATCG
AAAGACGATAGATAGGGAG

>5
GTGCCTTTATATTCTTTAACAAGGCTATATGTTTTAGCTTTGTAAGAAGGATGGCGATCTCGTACAGCGG
TACGATGTCCCAAGACACCACCCAAGACACCAATGAGAACACTACAAATTAAAGCGACTATAAAAATTAA
TATCCAAATAAAGTAACGTCGGTACCAAGGCAATAACTTTTTCTCTGCAGCCTTTTCGTTAAAGGCTTCA
TTGTTTTCGTTATTATTATTGCTATCAAGGTTTTCATGGTCATTATCGTGATCATCAATTTCAGTGTCTG
TATGATTGTCGATAACATCATTAGTTGACTCGTTCTTGCGTGCCGGGTCGTAAATATTCTCAAAGCTAGC
AGAGGACAACGCAGCAGATGAGAGACTATGGCGTGAATCTGTAGTGGAATCAGGAATACCCATTGCAAAA
GTAGTAAAAGTTAATCAAATTTCAACAAATTAAAGAAGGGCGAGAAAAATCAAAAAGAAAGAAAAGATAA
GAATAAAAAGAGCGGGAAGTAATGAGACACGATCAAATAAAAATATGCCAATCTGGTGAGCTATGCACCA
AGTTCAGAAATGGAAATAAAATGCTAGTAAAAATAACCAAACTCAAGGATATCTTGTTAAAGGGGAATAA
AAAAGGGAAATATGGGATAATGCTTTGTAGAGATGAGAAATGCGGTTTAAAATGAAAATAATTATTATCG
AAAGACGATAGATAGGGAG

>6
GTGCCTTTATATTCTTTAACAAGGCTATATGTTTTAGCTTTGTAAGAAGGATGGCGATCTCGTACAGCGG
TACGATGTCCCAAGACACCACCCAAGACACCAATGAGAACACTACAAATTAAAGCGACTATAAAAATTAA
TATCCAAATAAAGTAACGTCGGTACCAAGGCAATAACTTTTTCTCTGCAGCCTTTTCGTTAAAGGCTTCA
TTGTTTTCGTTATTATTATTGCTATCAAGGTTTTCATGGTCATTATCGTGATCATCAATTTCAGTGTCTG
TATGATTGTCGATAACATCATTAGTTGACTCGTTCTTGCGTGCCGGGTCGTAAATATTCTCAAAGCTAGC
AGAGGACAACGCAGCAGATGAGAGACTATGGCGTGAATCTGTAGTGGAATCAGGAATACCCATTGCAAAA
GTAATAAAAGTTAATCAAATTTCAACAAATTAAAGAAGGGCGAGAAAAATCAAAAAGAAAGAAAAGATAA
GAATAAAAAGAGCGGGAAGTAATGAGACACGATCAAATAAAAAAATGCCAATCTGGTGAGCTATGCACCA
AGTTCAGAAATGGAAATAAAATGCTAGTAAAAATAACCAAACTCAAGGATATCTTGTTAAAGGGGAATAA
AAAAGGGAAATATGGGATAATGCTTTGTAGAGATGAGAAATGCGGTTTAAAATGAAAATAATTATTATCG
AAAGACGATAGATAGGGAG

>7
GTGCCCTTATATTCTTTAACAAGGCTATATGTTTTAGCTTTGTAAGAAGGATGGCGATCTCGTACAGCGG
TACGATGTCCCAAGACACCACCCAAGACACCAATGAGAACACTACAAATTAAAGCGACTATAAAAATTAA
TATCCAAATAAAGTAACGTCGGTACCAAGGCAATAACTTTTTCTCTGCAGCCTTTTCGTTAAAGGCTTCA
TTGTTTTCGTTATTATTATTGCTATCAAGGTTTTCATGGTCATTATCGTGATCATCAATTTCAGTGTCTG
TATGATTGTCGATAACATCATTAGTTGACTCGTTCTTGCGTGCCGGGTCGTAAATATTCTCAAAGCTAGC
AGAGGACAACGCAGCAGATGAGAGACTATGGCGTGAATCTGTAGTGGAATCAGGAATACCCATTGCAAAA
GTAGTAAAAGTTAATCAAATTTCAACAAATTAAAGAAGGGCGAGAAAAATCAAAAAGAAAGAAAAGATAA
GAATAAAAAGAGCGGGAAGTAATGAGACACGATCAAATAAAAAAATGCCAATCTGGTGAGCTATGCACCA
AGTTCAGAAATGGAAATAAAATGCTAGTAAAAATAACCAAACTCAAGGATATCTTGTTAAAGGGGAATAA
AAAAGGGAAATATGGGATAATGCTTTGTAGAGATGAGAAATGCGGTTTAAAATGAAAATAATTATTATCG
AAAGACGATAGATAGGGAG

Central core chromosome II

>1
AGATTTGAGCAATAATTTCCTAAAACATTTACAATTAATGGGTTATTTTTTTTTT-CTAAAAATTGAAGG
AGCTTGTTTGATCTATTCCGTTATTAGTATGTAAATTTAAAAAATCGTATCCCTAACTAATTCATACTTA
AACATAATATGCAGATTTTAAAATAATTGTCCATATAGATAACACGCGGAATACTTAGAAAAGTAGAATT
GGCCGAAGCCAAAAAGGAAACATAGAAATCAACCAGGACTAACCAATGCTTCTTCATATTAACGAGTAAC
GCGGCTTACGCATCACAAAGCAGTACGTGTGAATAAATATTATTGGAAAACATTTTTCTTTAACCAGCTA
AACCGCTTAAAGAAAAATGAATAAGATACAAATTTTCAGGTTTTTGCTTTGATGCTAAAAACTCGTCGAA
TTCTAAAACCAAGAATTAAACGTACCCTTAAACTTTTTTTTTT-GATTTTCGTACTAATAATTATCGGAG
TTCGCGTTTTCATTTTAAAAAAA-GGTTTAGTAAATCTCAAATTGCGATACGAACAATGATAATGATATT
TTCAGATAAACGTTACTTAATAGCATCTTATAGTCATGGGCCATCTCAATATTACCATTAAACTTTTAAG
CAAAAAGATCATTTCTATTTTAAAAAACCTAATAAAAATTACCTTTTGTAAGTATACGTTTGCTAAACAA
TAAAAGAACGTAATACTAA

>2
AGATTTGAGCAATAATTTCCTAAAACATTTACAATTAATGGGTTATTTTTTTTTT-CTAAAAATTGAAGG
AGCTTGTTTGATCTATTCCGTTATTAGTATGTAAATTTAAAAAATCGTATCCCTAACTAATTCATACTTA
AACATAATATGCAGATTTTAAAATAATTGTCCATATAGATAACACGCGGAATACTTAGAAAAGTAGAATT
GGCCGAAGCCAAAAAGGAAACATAGAAATCAACCAGGACTAACCAATGCTTCTTCATATTAACGAGTAAC
GCGGCTTACGCATCACAAAGCAGTACGTGTGAATAAATATTATTGGAAAACATTTTTCTTTAACCAGCTA
AACCGCTTAAAGAAAAATGAATAAGATACAAATTTTCAGGTTTTTGCTTTGATGCTAAAAACTCGTCGAA
TTCTAAAACCAAGAATTAAACGTACCCTTAAACTTTTTTTTTT-GATTTTCGTACTAATAATTATCGGAG
TTCGCGTTTTCATTTTAAAAAAAAGGTTTAGTAAATCTCAAATTGCGATACGAACAATGATAATGATATT
TTCAGATAAACGTTACTTAATAGCATCTTATAGTCATGGGCCATCTCAATATTACCATTAAACTTTTAAG
CAAAAAGATCATTTCTATTTTAAAAAACCTAATAAAAATTACCTTTTGTAAGTATACGTTTGCTAAACAA
TAAAAGAACGTAATACTAA

>3
AGATTTGAGCAATAATTTCCTAAAACATTTACAATTAATGGGTTATTTTTTTTTT-CTAAAAATTGAAGG
AGCTTGTTTGATCTATTCCGTTATTAGTATGTAAATTTAAAAAATCGTATCCCTAACTAATTCATACTTA
AACATAATATGCAGATTTTAAAATAATTGTCCATATAGATAACACGCGGAATACTTAGAAAAGTAGAATT
GGCCGAAGCCAAAAAGGAAACATAGAAATCAACCAGGACTAACCAATGCTTCTTCATATTAACGAGTAAC
GCGGCTTACGCATCACAAAGCAGTACGTGTGAATAAATATTATTGGAAAACATTTTTCTTTAACCAGCTA
AACCGCTTAAAGAAAAATGAATAAGATACAAATTTTCAGGTTTTTGCTTTGATGCTAAAAACTCGTCGAA
TTCTAAAACCAAGAATTAAACGTACCCTTAAACTTTTTTTTT--GATTTTCGTACTAATAATTATCGGAG
TTCGCGTTTTCATTTTAAAAAAA-GATTTAGTAAATCTCAAATTGCGATACGAACAATGATAATGATATT
TTCAGATAAACGTTACTTAATAGCATCTTATAGTCATGGGCCATCTCAATATTACCATTAAACTTTTAAG
CAAAAAGATCATTTCTATTTTAAAAAACCTAATAAAAATTACCTTTTGTAAGTATACGTTTGCTAAACAA
TAAAAGAACGTAATACTAA

>4
AGATTTGAGCAATAATTTCCTAAAACATTTACAATTAATGGGTTATTTTTTTTTT-CTAAAAATTGAAGG
AGCTTGTTTGATCTATTCCGTTATTAGTATGTAAATTTAAAAAATCGTATCCCTAACTAATTCATACTTA
AACATAATATGCAGATTTTAAAATAATTGTCCATATAGATAACACGCGGAATACTTAGAAAAGTAGAATT
GGCCGAAGCCAAAAAGGAAACATAGAAATCAACCAGGACTAACCAATGCTTCTTCATATTAACGAGTAAC
GCGGCTTACGCATCACAAAGCAGTACGTGTGAATAAATATTATTGGAAAACATTTTTCTTTAACCAGCTA
AACCGCTTAAAGAAAAATGAATAAGATACAAATTTTCAGGTTTTTGCTTTGATGCTAAAAACTCGTCGAA
TTCTAAAACCAAGAATTAAACGTACCCTTAAACTTTTTTTTTT-GATTTTCGTACTAATAATTATCGGAG
TTCGCGTTTTCATTTTAAAAAAA-GGTTTAGTAAATCTCAAATTGCGA-----ACAATGATAATGATATT
TTCAGATAAACGTTACTTAATAGCATCTTATAGTCATGGGCCATCTCAATATTACCATTAAACTTTTAAG
CAAAAAGATCATTTCTATTTTAAAAAACCTAATAAAAATTACCTTTTGTAAGTATACGTTTGCTAAACAA
TAAAAGAACGTAATACTAA

>5
AGATTTGAGCAATAATTTCCTAAAACATTTACAATTAATGGGTTATTTTTTTTTT-CTAAAAATTGAAGG
AGCTTGTTTGATCTATTCCGTTATTAGTATGTAAATTTAAAAAATCGTATCCCTAACTAATTCATACTTA
AACATAATATGCAGATTTTAAAATAATTGTCCATATAGATAACACGCGGAATACTTAGAAAAGTAGAATT
GGCCGAAGCCAAAAAGGAAACATAGAAATCAACCAGGACTAACCAATGCTTCTTCATATTAACGAGTAAC
GCGGCTTACGCATCACAAAGCAGTACGTGTGAATAAATATTATTGGAAAACATTTTTCTTTAACCAGCTA
AACCGCTTAAAGAAAAATGAATAAGATACAAATTTTCAGGTTTTTGCTTTGATGCTAAAAACTCGTCGAA
TTCTAAAACCAAGAATTAAACGTACCCTTAAACTTTTTTTTTT-GATTTTCGTACTAATAATTATCGGAG
TTCGCGTTTTCATTTTAAAAAAA-GGTTTAGTAAATCTCAAATTGCGATACGAACAATGATAATGATATT
TTCAGATAAACGTTACTTAATAGCATCTTATAGTCATGGGCCATCTCAATATTACCATTAAACTTTTAAG
CAAAAAGATCATTTCTATTTTAAAAAACCTAATAAAAATTACCTTTTGTAAGTATACGTTTGCCAAACAA
TAAAAGAACGTAATACTAA

>6
AGATTTGACCAATAATTTCCTAAAACATTTACAATTAATGCGTTATTTTTTTTTTTCTAAAAATTGAAGG
AGCTTGTTTGATCTATTCCGTTATTAGTATGTAAATTTAGAAAATCGTATCCCTAACTAATTCATACTTA
AACCTAATATGCAGATTTTAAAATAATAGTCCATAAAGATAACACACGGAATACTTAAAAAAGTAGAATT
GGCCGAAGCCAAAAAGGAAACATAGAAATCAACCAGGACTAACCAATGCTTCTTCATATTAACGAGTAAC
--GGCTTACGCATCACAAAGCAGTACATGTGAATAAATATTATTGGAAAACATTTTTCTTTAACCAGCTA
AACCGCTTAAAGAAAAATGAATAAGATACAAATCTTCAGGTTTTTGCTTTGATGCTAAAAACTCGTCGAA
TTTTAAAACCAAGAATTAAACGTACCCTTCAACTTTTTTTTTTTGATTTTCGTACTAATGATTATCGGAG
TTCGCGTTTTCATTTTAAAAAAA-GGTTTAGTAAATCTCAAATTGCGATACGAACAATGATAATGATATT
TTCAGATAAACGTTACTTAATAGCATCTTATAGTCATGGGCCATCTCAATATTACCATTAAACTTTTAAG
CAAAAAGATCATTTCTATTTTAAAAAATCTAATAAAAATTACCTTTTGTAAGTATACGTTTGCTAAACAA
TAAAAGAACATATTACTAA

>7
AGATTTGAGCAATAATTTCCTAAAACATTTACAATTAATGGGTTATTTTTTTTTT-CTAAAAATTGAAGG
AGCTTGTTTGATCTATTCCGTTATTAGTATGTAAATTTAAAAAATCGTATCCCTAACTAATTCATACTTA
AACATAATATGCAGATTTTAAAATAATTGTCCATATAGATAACACGCGGAATACTTAGAAAAGTAGAATT
GGCCGAAGCCAAAAAGGAAACATAGAAATCAACCAGGACTAACCAATGCTTCTTCATATTAACGAGTAAC
GCGGCTTACGCATCACAAAGCAGTACGTGTGAATAAATATTATTGGGAAACATTTTTCTTTAACCAGCTA
AACCGCTTAAAGAAAAATGAATAAGATACAAATTTTCAGGTTTTTGCTTTGATGCTAAAAACTCGTCGAA
TTCTAAAACCAAGAATTAAACGTACCCTTAAACTTTTTTTTTT-GATTTTCGTACTAATAATTATCGGAG
TTCGCGTTTTCATTTTAAAAAAA-GGTTTAGTAAATCTCAAATTGCGA-----ACAATGATAATGATATT
TTCAGATAAACGTTACTTAATAGCATCTTATAGTCATGGGCCATCTCAATATTACCATTAAACTTTTAAG
CAAAAAGATCATTTCTATTTTAAAAAACCTAATAAAAATTACCTTTTGTAAGTATACGTTTGCTAAACAA
TAAAAGAACGTAATACTAA

>8
AAATTTGAGCAATAATTTCCTAAAACATTTACAATTAATGGGTTATTTTTTTTTT-CTAAAAATTGAAGG
AGCTTGTTTGATCTATTCCGTTATTAGTATGTAAATTTAAAAAATCGTATCCCTAACTAATTCATACTTA
AACATAATATGCAGATTTTAAAATAATTGTCCATATAGATAACACGCGGAATACTTAGAAAAGTAGAATT
GGCCGAAGCCAAAAAGGAAACATAGAAATCAACCAGGACTAACCAATGCTTCTTCATATTAACGAGTAAC
GCGGCTTACGCATCACAAAGCAGTACGTGTGAATAAATATTATTGGAAAACATTTTTCTTTAACCAGCTA
AACCGCTTAAAGAAAAATGAATAAGATACAAATTTTCAGGTTTTTGCTTTGATGCTAAAAACTCGTCGAA
TTCTAAAACCAAGAATTAAACGTACCCTTAAACTTTTTTTTTTT-GATTTCGTACTAATAATTATCGGAG
TTCGCGTTTTCATTTTAAAAAAA-GGTTTAGTAAATCTCAAATTGCGATACGAACAATGATAATGATATT
TTCAGATAAACGTTACTTAATAGCATCTTATAGTCATGGGCCATCTCAATATTACCATTAAACTTTTAAG
CAAAAAGATCATTTCTATTTTAAAAAACCTAATAAAAATTACCTTTTGTAAGTATACGTTTGCTAAACAA
TAAAAGAACGTAATACTAA

>9
AGATTTGACCAATAATTTCCTAAAACATTTACAATTAATGCGTTATTTTTTTTTTTCTAAAAATTGAAGG
AGCTTGTTTGATCTATTCCGTTATTAGTATGTAAATTTAGAAAATCGTATCCCTAACTAATTCATACTTA
AACCTAATATGCAGATTTTAAAATAATAGTCCATAAAGATAACACGCGGAATACTTAAAAAAGTAGAATT
GGCCGAAGCCAAAAAGGAAACATAGAAATCAACCAGGACTAACCAATGCTTCTTCATATTAACGAGTAAC
--GGCTTACGCATCACAAAGCAGTACATGTGAATAAATATTATTGGAAAACATTTTTCTTTAACCAGCTA
AACCGCTTAAAGAAAAATGAATAAGATACAAATCTTCAGGTTTTTGCTTTGATGCTAAAAACTCGTCGAA
TTTTAAAACCAAGAATTAAACGTACCCTTCAACTTTTTTTTTTTTGATTTCGTACTAATGATTATCGGAG
TTCGCGTTTTCATTTTAAAAAAA-GGTTTAGTAAATCTCAAATTGCGATACGAACAATGATAATGATATT
TTCAGATAAACGTTACTTAATAGCATCTTATAGTCATGGGCCATCTCAATATTACCATTAAACTTTTAAG
CAAAAAGATCATTTCTATTTTAAAAAATCTAATAAAAATTACCTTTTGTAAGTATACGTTTGCTAAACAA
TAAAAGAACATATTACTAA

>10
AGATTTGAGCAATAATTTCCTAAAACATTTACAATTAATGGGTTATTTTTTTTTT-CTAAAAATTGAAGG
AGCTTGTTTGATCTATTCCGTTATTAGTATGTAAATTTAAAAAATCGTATCCCTAACTAATTCATACTTA
AACATAATATGCAGATTTTAAAATAATTGTCCATATAGATAACACGCGGAATACTTAGAAAAGTAGAATT
GGCCGAAGCCAAAAAGGAAACATAGAAATCAACCAGGACTAACCAATGCTTCTTCATATTAACGAGTAAC
GCGGCTTACGCATCACAAAGCAGTACGTGTGAATAAATATTATTGGAAAACATTTTTCTTTAACCAGCTA
AACCGCTTAAAGAAAAATGAATAAGATACAAATTTTCAGGTTTTTGCTTTGATGCTAAAAACTCGTCGAA
TTCTAAAACCAAGAATTAAACGTACCCTTAAACTTTTTTTTTTT-GATTTCGTACTAATAATTATCGGAG
TTCGCGTTTTCATTTTAAAAAAA-GGTTTAGTAAATCTCAAATTGCGATACGAACAATGATAATGATATT
TTCAGATAAACGTTACTTAATAGCATCTTATAGTCATGGGCCATCTCAATATTACCATTAAACTTTTAAG
CAAAAAGATCATTTCTATTTTAAAAAACCTAATAAAAATTACCTTTAGTAAGTATACGTTTGCTAAACAA
TAAAAGAACGTAATACTAA

Table S3   Primers used to make probes used in hybridization analysis

Name	Sequence	Chromosome	Position
CENDistal1LF	CCCAAGAAACGTCATGTGTG	1	196555-196574
CENDistal1LR	CTACCAGGAGCCTTCAGTGC	1	197476-197457
CENDistal1RF	CACTTCGTGCAAAGCAAAAA	1	4745750-4745769
CENDistal1RR	ACAATTGCCAACCTCCTCAC	1	4746658-4746639
CENDistal2LF	TTGGTCTCGAAGGAGCATCT	2	751963-751982
CENDistal2LR	ATTCCACCAATTTCCCAACA	2	752829- 752810
CENDistal2RF	TTCGATAGGTCGGATTCCAG	2	3761023-3761042
CENDistal2RR	ATCTCTTGCCAACCACCAAC	2	3761895-3761876
CENDistal3LF	TCAAACAATCCATCGGACAA	3	501513-501532
CENDistal3LR	GATGCGGAAGCTAAATCGAG	3	502474-502455
CENDistal3RF	GTGACGTAAATGCCCGAGTT	3	2004488-2004507
CENDistal3RR	AGATTCGGGTTTCAGTGGTG	3	2005446-2005465
CENproximal1LR	AAAGGCTGTCGGTGTTATGG	1	3733956-3733937
CENproximal1LF	CGGTGAACCTGACAATGATG	1	3733183-3733202
CENproximal1RF	CGTCAATCCTCAATTCAGAGTTCC	1	3792899-3792922
CENproximal1RR	TATGTCAAAGAAGCCTCGATAAAGAAG	1	3793712-3793738
CENproximal2LF	GATCGATATCACCAGGCTTGGTC	2	1572261-1572283
CENproximal2LR	GCGAGAAATTGCCAATTGCCGAC	2	1573046-1573068
CENproximal2RF	AAGTGACATCACAAGTCCGACTTC	2	1650281-1650304
CENproximal2RL	AACTTGGAAACCAATAAAATTGAG	2	1651324-1651301
CENproximal3LF	AATAGGGAAGCCGATTGTTCTTACAGATGT	3	1061733-1061762
CENproximal3LR	TATCTATGGAAAGCATTAGAACGA	3	1062572-1062549
CENproximal3RF	GTTCGTCAATTTATTAATCATTAG	3	1145013-1145036
CENproximal3RR	AACCCCTTACACCTCCTCAAGATC	3	1145732-1145709

Table S4 Primers used to amplify DNA for diversity analys

Sequence	Primer F	Sequence	Primer R	Sequence
SPBC660.16 intron 3	SPBC660L	gccgacaaggggtaagtttt	SPBC660R	ttacctttggcttcgtttgc
Central core of centromere of chromosome II	CNT21L	cacagaagcgagacatgttt	CNT21R	ggataagacacttcgccaaaa
TER	TRC19F	tgtttgatctacctcgtttatattca	TRC1409R	aaaaagaggcctggacatca
TER	TRC667F	aacctaaaacgcgctcaaga	TRC1983R	ttgaatgtaatgttagcccttaaaa
CEN2:left flank	CENFlank2LF	gatcgatatcaccaggcttggtc	CENFlank2LR	gcgagaaattgccaattgccgac
CEN2:right flank	CENFlank2iRF	gcactgctgttacggtcaag	CENFlank2iRR	aagctggttggctttttatcc
TER; I-3113975-3114540	TRCflank1F	gccagatcaaatggctcaat	TRCflank1R	acaacgttgggatcaagagg
TER; I-3194538-3195201	100TRCFlank1F	ttaaacgctcttgcgtcctt	100TRCFlank1R	ctcaccgcatctcgtactca

Table S5 The proliferative lag (time to initiate proliferation), proliferative rate (population doubling time) and proliferative efficiency of natural *S. pombe* isolates over 42 environments

Table S5 is available for download at http://www.g3journal.org/lookup/suppl/doi:10.1534/g3.111.001123/-/DC1 as a text file.

The proliferative lag (time to initiate proliferation), proliferative rate (population doubling time) and proliferative efficiency (change in population density) were extracted from high density growth curves (n=2) of natural *S. pombe* isolates (N=2) over 42 environments. The data set shows proliferation variables relative the *S. cerevisiae* universal type strain BY4741, log2 [isolate/BY4741], in each environment.
