# Supplementary material for: A Geographically Diverse Collection of Schizosaccharomyces pombe Isolates Shows Limited Phenotypic Variation but Extensive Karyotypic Diversity
Source: G3 (Bethesda). 2011 Dec 1;1(7):615–26. doi: 10.1534/g3.111.001123 (PMC3276172; doi:10.1534/g3.111.001123)
Supplement: Supporting Information [file supp_1.7.615_FigureS1.pdf]

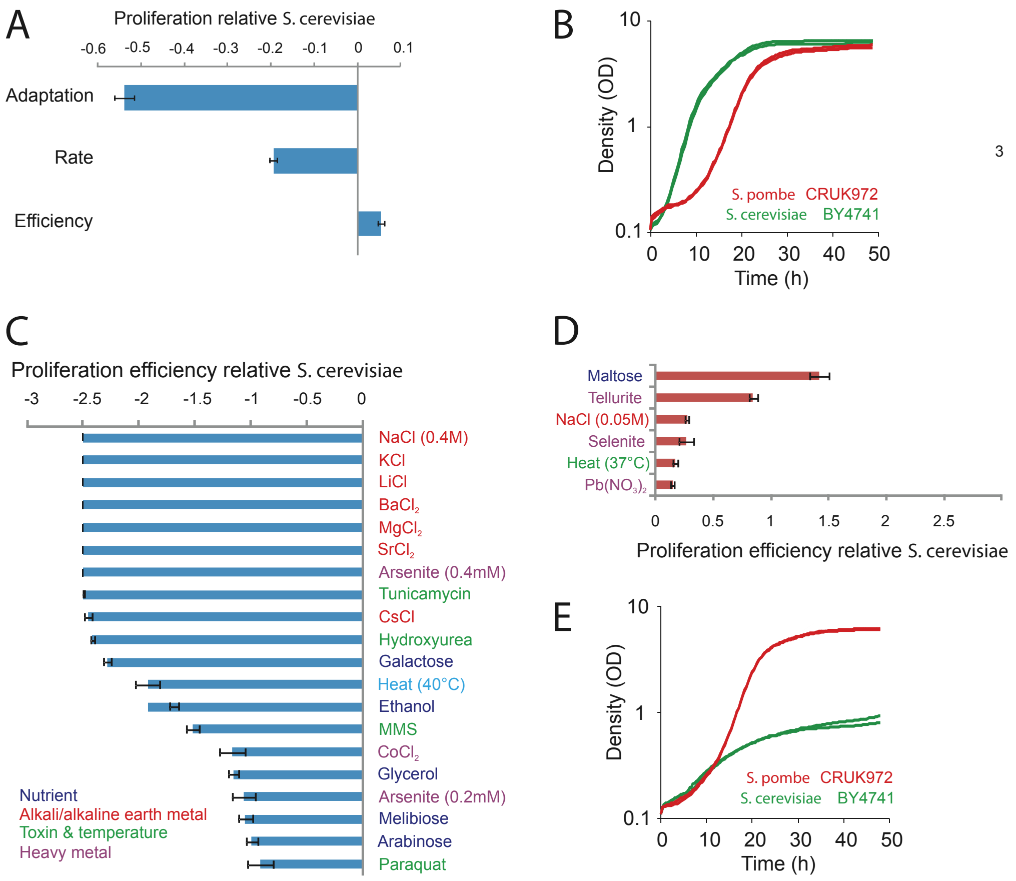

Figure S1 *S. pombe* proliferates more slowly than *S. cerevisiae* in a wide range of environments. A-B) *S. pombe* shows a much longer mitotic lag phase and a slower rate of mitotic proliferation than the *S. cerevisiae* universal type strain BY4741 in basal conditions (Synthethic Defined medium). A) The proliferative lag, rate and efficiency of *S. pombe* natural isolates in basal conditions were calculated and put in relation to the corresponding measures of BY4741 (Log2 [isolate/BY4741]). Mean and Standard Error of the Mean are displayed. B) Proliferation of the *S. pombe* type strain 972h- (N=2) in basal conditions. BY4741 (N=2) is shown as reference. C) Top 20 environments in which the *S. pombe* proliferative efficiency is inferior to that of the *S. cerevisiae* universal type strain BY4741. A relative proliferative efficiency, (Log2 [isolate/BY4741]), was calculated for all *S. pombe* natural isolates. Means and Standard Error of the Means for each environment are displayed. D) Top 5 environments in which the *S. pombe* proliferative efficiency is superior to that of the *S. cerevisiae* universal type strain BY4741. A relative proliferative efficiency, (Log2 [isolate/BY4741]), was calculated for all *S. pombe* natural isolates. Mean and Standard Error of the Mean for each environment are displayed. E) Proliferation of the *S. pombe* type strain 972h- (N=2) using maltose as sole carbon source. BY4741 (N=2) is shown as reference.
