# Supplementary material for: A Geographically Diverse Collection of Schizosaccharomyces pombe Isolates Shows Limited Phenotypic Variation but Extensive Karyotypic Diversity
Source: G3 (Bethesda). 2011 Dec 1;1(7):615–26. doi: 10.1534/g3.111.001123 (PMC3276172; doi:10.1534/g3.111.001123)
Supplement: Supporting Information [file supp_1.7.615_FigureS2.pdf]

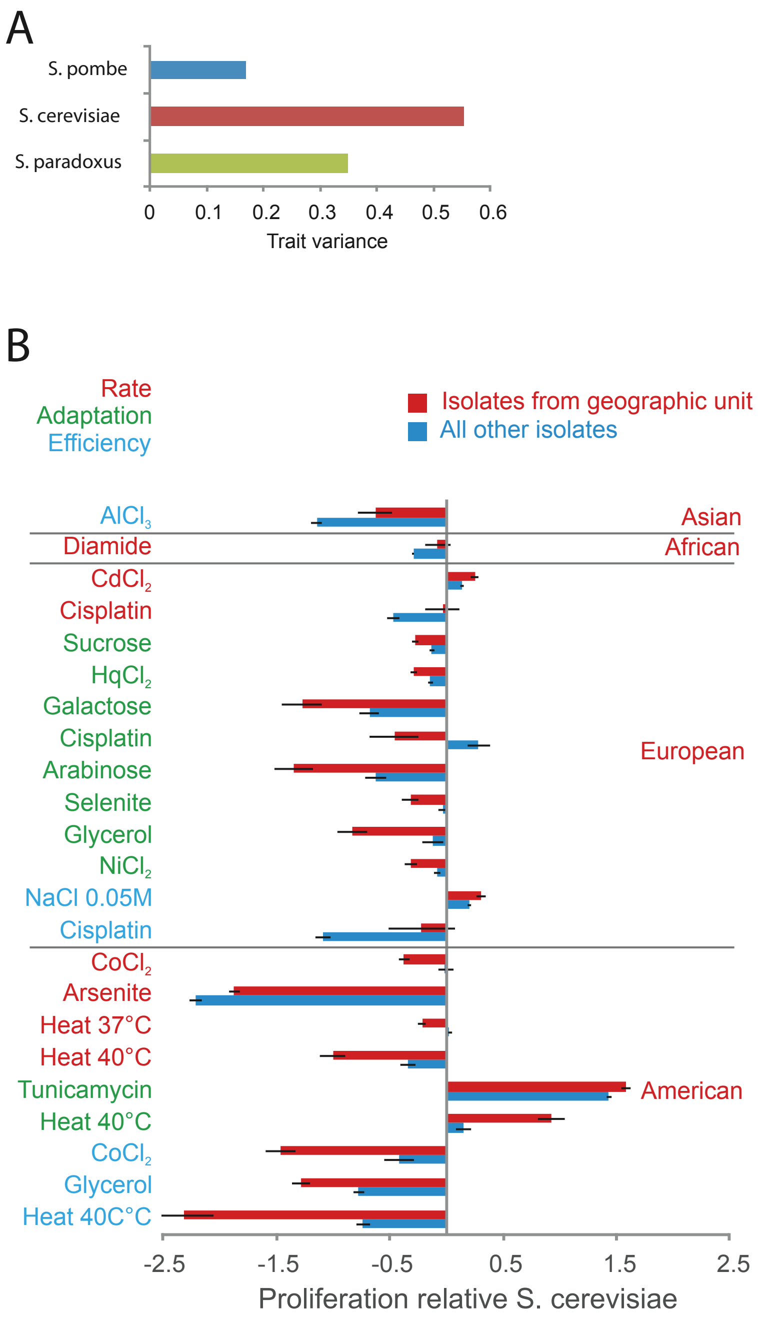

Figure S2 Natural trait variation in *S. pombe* is defined by geographic boundaries. A) Trait variation in natural isolates *S. pombe* is substantially smaller than trait variation in *S. cerevisiae* and *S. paradoxus*. Trait variance within *S. pombe* was calculated for each trait separately and a mean over all traits was formed, excluding environments in which more than 50% of S. pombe isolates failed to proliferate. Trait variance in *S. cerevisiae* and *S. paradoxus* was obtained from (ref) in which the mitotic proliferation in a similar, but wider array of environments was quantified using an identical experimental set-up. Error bars represent standard errors of the mean. B) Traits for which *S. pombe* isolates from one geographic area differ significantly (Students ttest, FDR=5%) from all other *S. pombe* isolates. Means and Standard Error of the Means are displayed.
