# Supplementary material for: A Geographically Diverse Collection of Schizosaccharomyces pombe Isolates Shows Limited Phenotypic Variation but Extensive Karyotypic Diversity
Source: G3 (Bethesda). 2011 Dec 1;1(7):615–26. doi: 10.1534/g3.111.001123 (PMC3276172; doi:10.1534/g3.111.001123)
Supplement: Supporting Information [file supp_1.7.615_FigureS3.pdf]

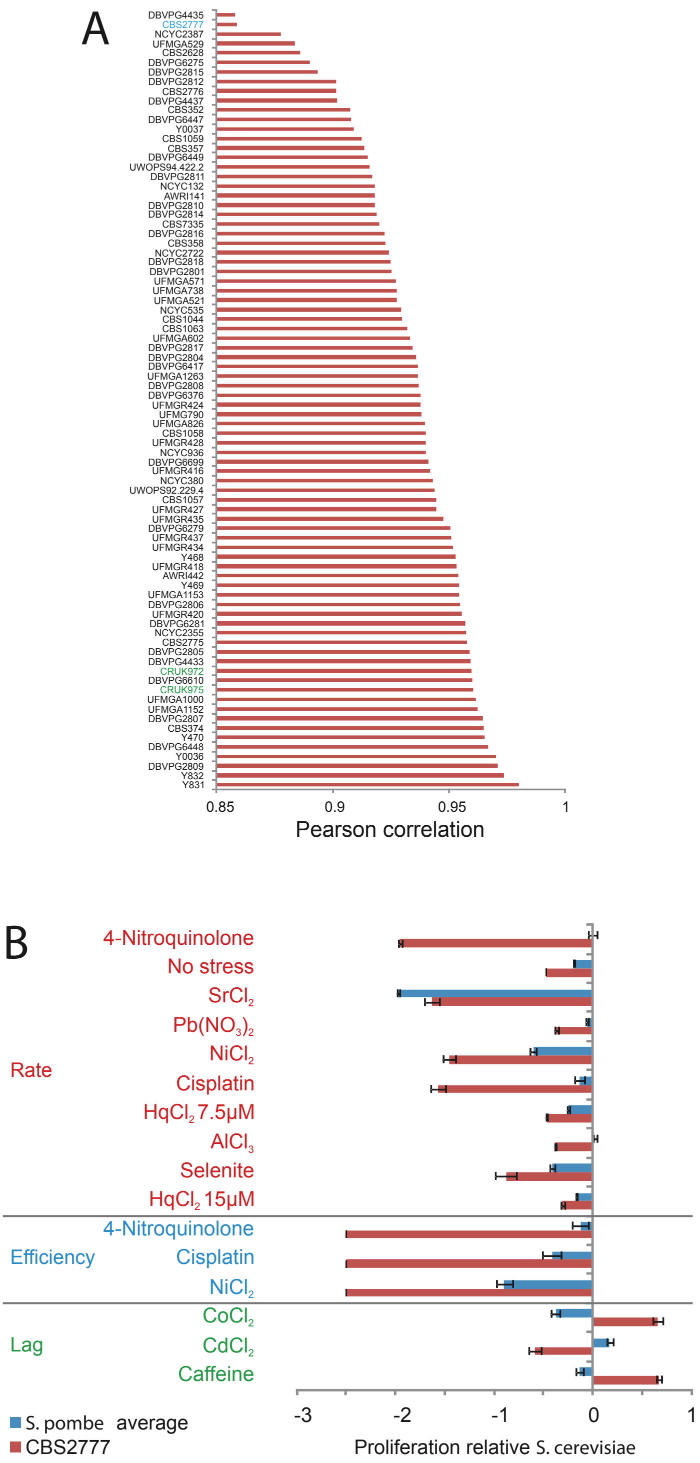

Figure S3 The *S. pombe* karyotype extreme, CBS2777, shows abnormal proliferation patterns. A) *S. pombe* mean trait profile was calculated over all traits and the similarity (Pearson correlation) between the mean trait profile and the trait profile of each individual *S. pombe* isolate was calculated. Isolates were ranked according to degree of similarity. The *S. pombe* karyotype extreme CBS2777 (blue) and the universal reference strains 972h- and 975h+ (green) are indicated with color. B) Traits for which the *S. pombe* karyotype extreme CBS2777 (N=2) differ significantly (Students ttest, FDR=5%) from all other *S. pombe* isolates. Means and Standard Error of the Means are displayed.
