# Supplementary material for: A Geographically Diverse Collection of Schizosaccharomyces pombe Isolates Shows Limited Phenotypic Variation but Extensive Karyotypic Diversity
Source: G3 (Bethesda). 2011 Dec 1;1(7):615–26. doi: 10.1534/g3.111.001123 (PMC3276172; doi:10.1534/g3.111.001123)
Supplement: Supporting Information [file supp_1.7.615_FigureS4.pdf]

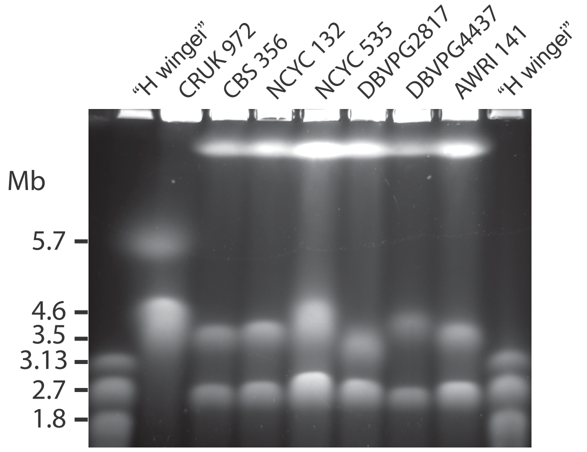


**Figure S4** A karyotype re-arrangement shared between the *S. pombe* type strain CBS 356 and five other strains. DNA was extracted from the *S. pombe* laboratory strain CRUK 972 , the type strain, CBS 356 and five other strains, NCYC 132, NCYC535, DBVPG2817, DBVPG4437 and AWRI 141 and analysed by pulsed field gel electrophoresis and ethidium bromide staining. The markers used in the flanking tracks were obtained from Bio-Rad and marketed as chromosomal DNA derived from *Hansenula wingei*. However we have prepared chromosomal DNA from the *Hansenula wingei* type strain and do not observe the same pattern, thus the label is in parentheses. The variability of the chromosome 2 is characteristic of the presence of the rDNA.
