# Supplementary material for: A Geographically Diverse Collection of Schizosaccharomyces pombe Isolates Shows Limited Phenotypic Variation but Extensive Karyotypic Diversity
Source: G3 (Bethesda). 2011 Dec 1;1(7):615–26. doi: 10.1534/g3.111.001123 (PMC3276172; doi:10.1534/g3.111.001123)
Supplement: Supporting Information [file supp_1.7.615_FigureS5.pdf]

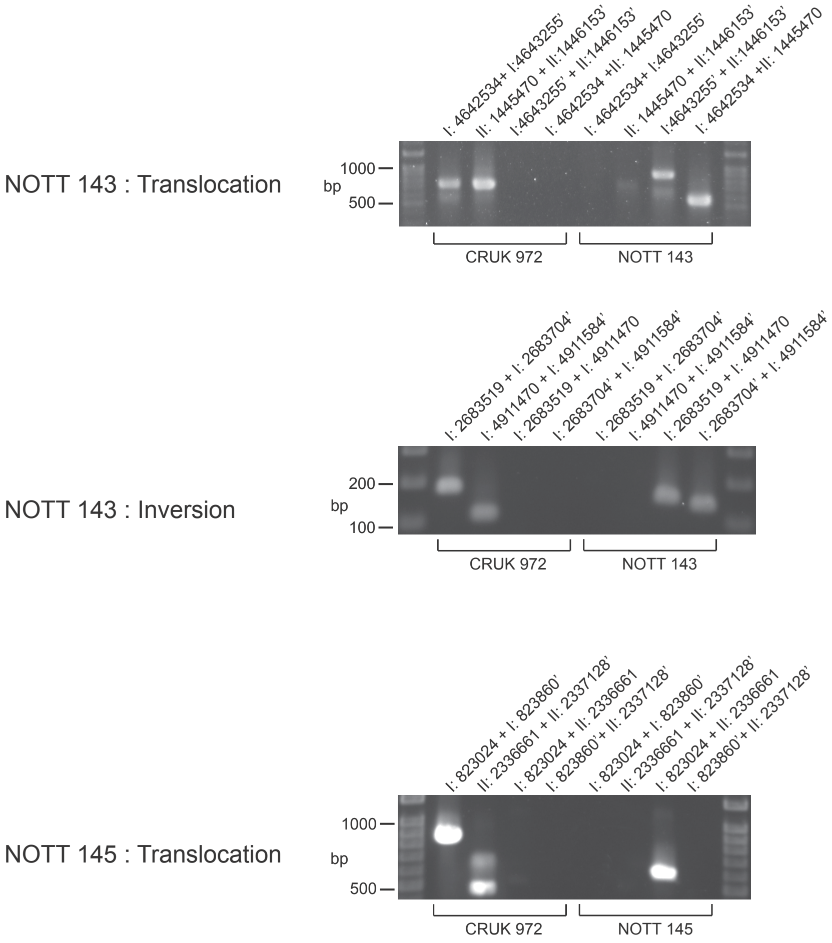

Figure S5 Identification of the sites of re-arrangements in NOTT 143 and NOTT 145. DNA was analysed by PCR using the indicated primers for the three re-arrangements identified in the CGH analysis of strains NOTT 143 and NOTT 145. CRUK 972 DNA was used as the control. The numbers refer to the positions of the 5' residue of the respective primers on the assembly of the laboratory strain. The ' indicates that the primer corresponds to the complementary strand of the laboratory strain assembly.
