# Supplementary material for: A Geographically Diverse Collection of Schizosaccharomyces pombe Isolates Shows Limited Phenotypic Variation but Extensive Karyotypic Diversity
Source: G3 (Bethesda). 2011 Dec 1;1(7):615–26. doi: 10.1534/g3.111.001123 (PMC3276172; doi:10.1534/g3.111.001123)
Supplement: Supporting Information [file supp_1.7.615_FigureS6.pdf]

Central Core Centromere II

A

Position

Haplotype: abundance — Positions: 1621091, 1621094, 1621110, 1621127, 1621146, 1621261, 1621265, 1621288, 1621290, 1621324, 1621340, 1621354, 1621381, 1621420, 1621477, 1621497, 1621522, 1621523, 1621606, 1621615, 1621628, 1621636, 1621660, 1621694, 1621748, 1621763, 1621795, 1621799

```
1:26    T C A A G TCGTA C - T - T G A T C GC C C A A T T - C C C
3:1     . . . . . ..... T - . - . . . . . .. . . . . . . - . . .
2:1     . . . . . ..... . T . - . . . . . .. . . . . . . - . . .
5:1     . . G . . ..... . - . - . . . . . .. . . . . . . - . . .
8:1     . . . . . ..... . - . - . . . . . .. . . . . . . - . . T
4:1     . . . . . ----- . - . - . . . . . .. . . . . . . - . . .
7:1     . . . . . ----- . - . - . . . C . .. . . . . . . - . . .
10:1    . . . T . ..... . - . - . . . . . .. . . . . . . - . . .
6:6     A T . . A ..... . - C A G A G . T -- T T T T G C A G G .
9:1     A T . . A ..... . - C A G A G . T -- T C T T G C A G G .
```

B

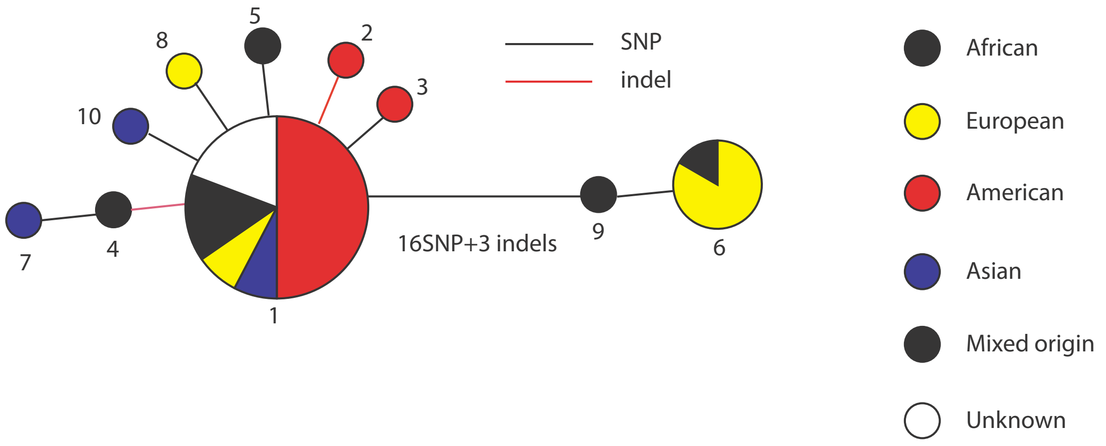

Figure S6 SNPs and haplotype structure in the central core of *S. pombe* chromosome II. A, The sequences shown in the text were analysed using the FABbox suite to identify informative residues B. The sequences shown in A were analysed for their relatedness using Network (Version 4.6.0.0, Fluxus Technology http://www.fluxus-engineering.com/sharenet.htm) to produce the network of haplotype relationships based on a median joining network using default parameters. The numbers adjacent to the nodes in the network refer to the specific central core haplotypes listed in A. The areas of the individual nodes are proportional to the numbers of compound haplotypes that contain the particular central core haplotype. The colors refer to the geographical origins of the respective haplotypes as indicated. Also detailed are the numbers and types of mutation separating the respective nodes.
