# Supplementary material for: A Geographically Diverse Collection of Schizosaccharomyces pombe Isolates Shows Limited Phenotypic Variation but Extensive Karyotypic Diversity
Source: G3 (Bethesda). 2011 Dec 1;1(7):615–26. doi: 10.1534/g3.111.001123 (PMC3276172; doi:10.1534/g3.111.001123)
Supplement: Supporting Information [file supp_1.7.615_FigureS7.pdf]

CEN2 L; 659bp
Haplotype: abundance

Haplotype: abundance	**1572349**	1572524	1572778	1572987
1:17	G	C	A	C
2:19	A	.	.	.
3:1	A	T	.	.
4:2	A	.	.	G
5:1	A	.	G	.

CEN2 R; 719bp
Haplotype: abundance

Haplotype: abundance	1658038	**1658392**	1658425	**1658455**	1658566
1:23	T	T	A	G	A
2:3	.	.	C	.	.
3:4	.	C	.	.	.
4:1	C	.	.	.	.
5:1	.	C	.	.	T
6:7	.	C	.	A	.
7:1	C	C	.	.	.

CEN2 compound haplotypes
Haplotype: abundance

Haplotype: abundance	**1572349**	**1621091**	**1658392**	**1658455**
1:17	G	T	T	G
2:6	A	T	C	G
3:10	A	T	T	G
4:7	A	A	C	A

Figure S7 SNPs flanking the centromere and inormative SNPs used in four gamete test. The figure illustrates the positions of the SNPs identified in the sequences flanking the centromere of chromosome II. Informative SNPs are shown in bold. The four compound haplotypes composed of informative SNPs are indicated below.
