# Supplementary material for: A Geographically Diverse Collection of Schizosaccharomyces pombe Isolates Shows Limited Phenotypic Variation but Extensive Karyotypic Diversity
Source: G3 (Bethesda). 2011 Dec 1;1(7):615–26. doi: 10.1534/g3.111.001123 (PMC3276172; doi:10.1534/g3.111.001123)
Supplement: Supporting Information [file supp_1.7.615_FigureS9.pdf]

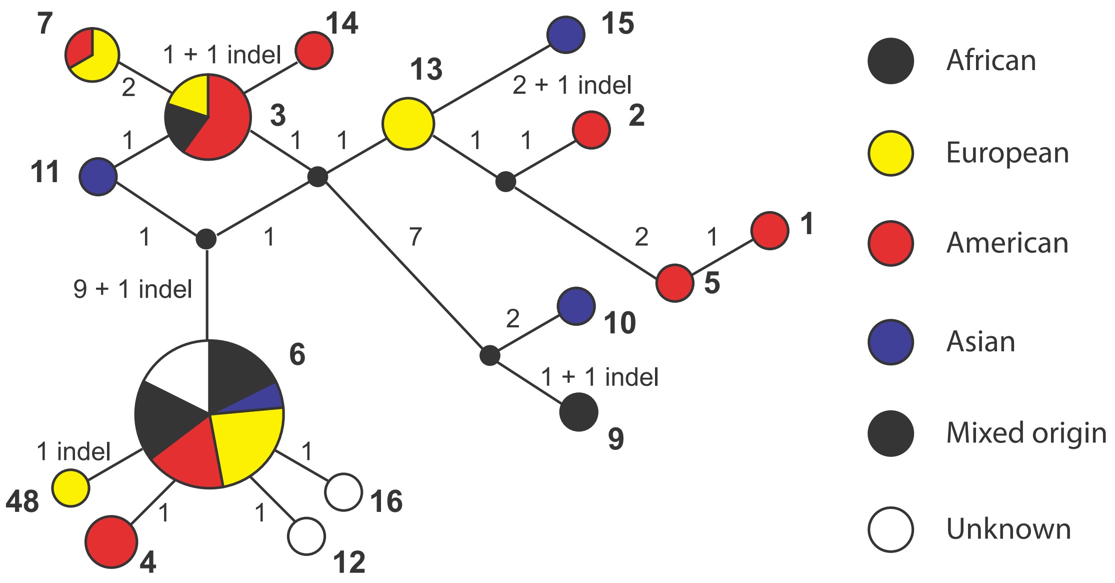


**Figure S9** Network of haplotypes at the TER gene. The sequences shown in figure S8 were analysed as in Figure S6. The numbers in bold refer to the haplotypes indicated in S8. The areas of the individual nodes are proportional to the numbers of compound haplotypes that contain the particular haplotype. The numbers between adjacent to the branches refer to the number of SNPs or indels separating the respective nodes. The colors refer to the geographical origins of the respective haplotypes as indicated.
