# Supplementary material for: A Geographically Diverse Collection of Schizosaccharomyces pombe Isolates Shows Limited Phenotypic Variation but Extensive Karyotypic Diversity
Source: G3 (Bethesda). 2011 Dec 1;1(7):615–26. doi: 10.1534/g3.111.001123 (PMC3276172; doi:10.1534/g3.111.001123)
Supplement: Supporting Information [file supp_1.7.615_FigureS10.pdf]

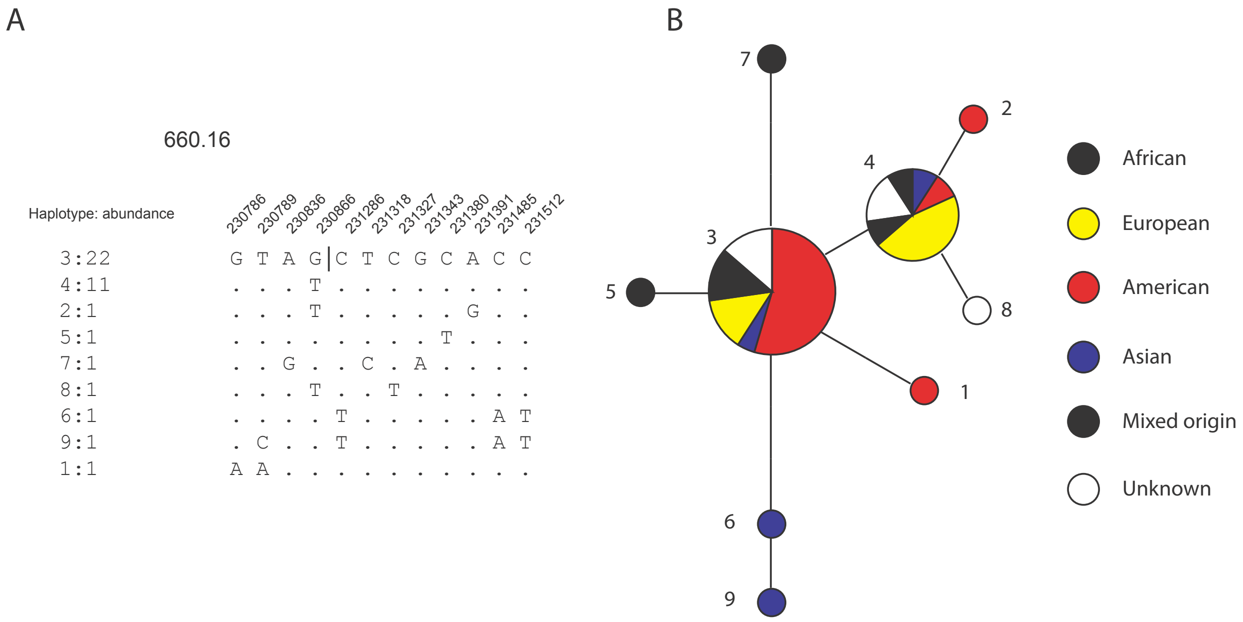


**Figure S10** SNPs identified in intron 3 of the SPBC660.16 gene. A**.** The figure illustrates the positions of the SNPs identified in the third intron of the SPBC660.16 gene. **B.** The sequences shown in A were analysed as in Figure S6. The numbers refer to the haplotypes indicated in A. The areas of the individual nodes are proportional to the numbers of compound haplotypes that contain the particular haplotype. The colors refer to the geographical origins of the respective haplotypes as indicated. The numbers of mutation separating the respective nodes are proportional to the distances between them.
