# Supplementary material for: A Geographically Diverse Collection of Schizosaccharomyces pombe Isolates Shows Limited Phenotypic Variation but Extensive Karyotypic Diversity
Source: G3 (Bethesda). 2011 Dec 1;1(7):615–26. doi: 10.1534/g3.111.001123 (PMC3276172; doi:10.1534/g3.111.001123)
Supplement: Supporting Information [file supp_1.7.615_FileS1.pdf]

File S1

Supporting Text

1 Strain collection

We used the web, the scientific literature and personal contacts to identify strain collections holding isolates of fission yeast. This led to a collection which includes 84 isolates of *S. pombe* (supplementary data Table 1). Each isolate in our collection retains its original designation. All but one of the strains were haploid as judged by DNA staining. The one strain; NCYC 2355, that was diploid yielded isogenic haploid clones upon re-streaking and we worked with one of these and for this reason it is marked with an asterisk. DBVPG 4433 is listed as *Zygosaccharomyces* but was found to contain fission yeast upon colony purification by GL.

2 Structural Analysis of re-arranged chromosomes

2.1 Analysis of Nott 143, Nott 145 and other strains containing re-arrangements involving sequences on chromosomes I and II of the laboratory strain.

As shown in the main text chromosome 1 in NOTT 143 was a derivative of chromosome II and chromosome 2 was the reciprocal chromosome I derivative. Micro-array analysis showed that the translocated sequences on NOTT 143 chromosome 1 were derived from two discontinuous regions of laboratory strain chromosome I; from the telomere of the left arm of chromosomes I to sequences between 2,683,519 and 2,683,704 and from sequences between 4,642,534 and 4,643,255 to sequences between 4,911,470 and 4,911,584. This pattern suggested a compound re-arrangement in which sequences from a variant of chromosome I containing a large pericentric inversion were translocated onto chromosome II. We used the micro-array data to design primers flanking hypothetical breakpoints and confirmed this compound re-arrangement by PCR (supplementary data; figure 2) and determined the identity of the breakpoint sequences and in particular the existence of a 2,227, 883 bp inversion across the centromere of chromosome I of the laboratory strain with respect to NOTT 143. The breakpoint on NOTT 143 chromosome 2 disrupted the gene SPAC30C2.07-1 and generated a truncated fusion protein of unknown functional significance (see below). We wanted to determine whether the inversion was ancestral or derived and we therefore analysed the entire collection for the presence of the inversion by PCR. All of the strains in the collection with the exception of DBVPG2805, NCYC 3092, DBVPG6610, DBVPG 4433, DBVPG 6279, DBVPG 6699, CRUK 972, CRUK 975 contained the sequence organization seen in NOTT 143 with respect to the inversion breakpoints. This suggested that the sequence arrangement in the laboratory strain was a derived trait and we confirmed this by comparison with the arrangement of the relevant sequences in *S. octosporus* and *S. cryophilus* (not shown). In addition to the inversion NOTT 145 was re-arranged by a translocation of between II R and I L giving rise to a derived chromosome 1 that was larger than the laboratory strain chromosome I and a derived chromosome 3 that at 3.2Mb was

slightly smaller than the rDNA containing chromosome. We were able to identify the breakpoint on NOTT 145 chromosome 3 but not on chromosome 1. We used two sets of primers suggesting that the failure to identify the chromosome 1 breakpoint may be due non-reciprocity. In NOTT 145 the chromosome 3 breakpoint was associated with a C-terminal truncation of SPBC24C6.09C-1. Otherwise all breakpoints were reciprocal with respect to the laboratory strain sequence with the exception of the chromosome I inversion, which involved the loss of a single residue (below). Filter hybridization analysis indicated that the re-arrangements in NOTT 140 and 142 were simple reciprocal translocations and they were not characterized further. Chromosome 1 in CBS 356 contained all of the chromosome II sequences used in the hybridization analysis as well as both of the distal sequences from chromosome I. Chromosome 2 of CBS 356 corresponds to chromosome III of the laboratory strain and chromosome 3 to a deleted derivative of chromosome I. We have not carried out any further analysis of this strain. Micro-array analysis of the chromosomes of NOTT 138 show that a 1.7Mb block of sequence from chromosome II has been transposed onto chromosome I to generate a derivative of chromosomes I and II corresponding to chromosome 1 and 3 of NOTT 138. Chromosome 1 of NOTT 140 contains sequences from the centromere proximal regions of chromosome I, distal I R and distal II L while NOTT 140 chromosome 2 contains sequences from the proximal regions of chromosome II, distal II R. and distal I L This result is suggests a exchange of material that is normally present on the distal left arms of chromosomes I and II of the laboratory strain giving rise to a derived II that is about 600kb smaller than chromosome II of the laboratory strain and a derived I that is larger than laboratory strain chromosome I by a similar amount. NOTT 140 contains the ancestral pericentric inversion of chromosome I and so the I R sequences are now on the left arm of the chromosome 1 in this strain.

2.2 Sequences of breakpoints in strains NOTT 143 and NOTT 145

NOTT 143: chromosome 1 breakpoint

```
I: 4642748
ccaaacatcagatcagtaagtggagcatttgccgagtccctaagcacactatctcctttctg/
I: 4642687
II: 1445844
/tatatttattgctactatcgcttttagaacatcatgttattaataatccgtaactagaccgtttaat
II: 1445910
```

NOTT 143: chromosome 2 breakpoint

```
II: 1445778
cctgtgcatgatttacttttaaattcagggttaattaattactttccatcatttcctcctctccat/
II: 1445844 / I: 4642686
/accatccacatgattagagtcaaacaaaacagttttttttcggtctccgatgtncttgaancgagga
caaga
I: 4642617
```

Region of homology highlighted

Disruption of SPAC30C2.07-1 (842 amino acids in CRUK 972) by the introduction of premature stop codon

I-4,642,495

atgcttcatttttatttcattcaggatcttcatcaaatagaaac**tcatcgccgaaagaatcctatga gcttctt**
M L H F L F H S G S S S N R N **S S P K E S Y E L L**
catggtttggacaaacaatatcaaagtactaaggatgtaacgtttcgtcttgtcctcgttcaagacat cggagac
H G L D K Q Y Q S T K D V T F R L V L V Q D I G D

I: 4642686 / II: 1445844

cgaaaaaaaactgttttgtttgactctaatcatgtggatggt/atggagaggaggaaatga 210
R K K T V L F D S N H V D G M E R R K * 69 amino acids

NOTT 145: chromosome 3 breakpoint

II:2336713
ctaaaattcgccattcaaatacttgggacatcctttacccttaggggtacgaaggataatcatgggcc
atctgggtttgatttcttctctgttaacacgagcgcgatgttggatgtcatgaatgcgttcaacggcc
caatc<u>**cat**</u>a/
II: 2336858 / I: 823412
/taattacgtggagctaccttaaattcaattaggtagtttaaaatctatcccagaaagctaacttact
gagaagtcctcgctaaaccatattagcgcatgtagcatttaatgaattattcgtgtggatattagata
actgatttaggttgatttttgtagcttatacaattnccttttttctttttggtataataaagccaaaat
cgtaaacaaagatgaattggaataaacattctttcacaatacttccacgagttcancatcgccatgtg
cgaacctcgttatatctttgaccntcgaaattcttgnagtccctgtcc
I: 823099
Potential new ATG indicated giving a C terminal product of SPBC24C6.09C-1
1596 nt; 532 amino acids truncation of CRUK 972 protein

Common chromosome I inversion (2,227,883 bp) breakpoints:

Left breakpoint

I: 2683567
tgttttcaaaatataaccacgggtaagtaatacagttggcatacaacatttgggtaacc
toccata / I:2683632 / I: 4911514
gcaagctagatctagattaatgctataggcatacnaaac
I: 4911476

Right breakpoint: underlined residue lost

I: 2684324
Agatcttagcaaggtttcggatatgtcatgggtaaagttcgatngctttgtgcatctccgttagcagc
tgcatcaagcgagtatccaacgatgatcatctcgattcgttttgtgtgttcatgaaacccacgccttg
cgtatgaagttgcccactttgtatgacaatgagataatcttacactgcaataaggccatatacccgta
attggtgggnacnaccactcacattccaaagtccaactcaatagtgaagaaattcatcattggcaaca
agtcatcaacacttcgttgctgttggacccttctaccaaacgttctttattgacaactttgaacggct
tcggctgcnttcccgagccctgtaacgcctatctttcatgtggctcggaatgtaaaccagaaactcag
acaaataccgctgttgatcgcgctgatgatatttgtttatcgaggctgtgttcatttttcttagtcat
tttagcgtaacgaactgacaccgaaaaatatcaagcaatcaagaagatccatctgtccaagcaccaac
cacatctttgctcgataatcaaacttggattaccgatagttaaaggtgaaggatcggttttgctggtt
tcattcgattttccaaacatcgtcgattcagtagactggggttgagtcagtatccaagccattcttgc
gctcaattgataat/<u>**t**</u> / I: 2683632 / I: 4911515
gtcatataacgaccagatttactcttgaccgacttcatagcagcataattagcntntccnnngncata
aaagaaaaaatttgctatctccactctcacaaggttagcgaaggtttgtgcatttgacagcaggttg
gttaaattcgttaagcttccaagagatataaatatagtggctcgaatttgacttatccagtcagtcat
tcattgctgaagttgttgggttggccag I: 4911745

3 Quantifying natural trait variation in *S. pombe*;

Strains were stored in 20% glycerol at -80° C. Strains were subjected to high throughput phenotyping by micro-cultivation (n=2) in an array of environments essentially as described (PMID: 12489126, PMID:18721464). First, strains were inoculated in 350 µl of YES medium (5% yeast extract, 3% glucose, 225mg/L histidine, 225mg/L adenine, 225mg/L leucine and 225mg/L uracil) and incubated in two serial rounds of pre-cultivation for 48h-72 h at 30C°. For experimental runs, strains were inoculated to an OD of 0.05 - 0.1 in 350 µl of YES medium (3% glucose was replaced by 3% of alternative carbon sources where indicated) in the relevant environments and micro-cultivated for 48 or 72h in a Bioscreen analyzer C (Growth curve Oy, Finland). Environments were: 4-nitroquinolone (0.3µg/ml), $AlCl_3$ (0.2mg/ml), arabinose (3%; carbon source), Arsenite (0.2, 2mM) $BaCl_2$ (25mM), Caffeine (1.5mg/ml), $CdCl_2$ (25µM), Cisplatin (100µg/ml), $CoCl_2$ (0.25mM), CsCl (12.5mg/ml), $CuCl_2$ (5mM), Cycloheximide (0.05 µg/ml), Diamide (1.4mM), EMS (0.3%), Ethanol (3%; carbon source), Galactose (3%; carbon source), Glycerol (3%, carbon source), Heat (37, 40°C), $HqCl_2$ (7.5 15µM), Hydroxyurea (8mg/ml), KCl (1.45 M), LiCl (225mM), Maltose (3%; carbon source), Melibiose (3%; carbon source), $MgCl_2$ (1.25mM), MMS (0.0015%), $MnCl_2$ (0.1mM), NaCl (0.05, 0.1, 0.4 M), $NiCl_2$ (0.75mM), Paraquat (200µg/ml), $Pb(NO_3)_2$ 0.4mM, Selenite (0.1mM), $SrCl_2$ (25mg/ml), Sucrose (3%; carbon source), Tellurite (30.5mM) and Tunicamycin (1µg/ml). All incubations were at 30°C (±0.1°C), except for when heat stress was applied (see above). Pre-heating time was set to ten minutes. Plates were subjected to shaking at highest shaking intensity with 60s of shaking every other minute. Optical density (OD) was recorded using a wide band (450-580 nm) filter every 20 minutes. Strains were run in duplicates on separate plates and normalized to four replicates of the *S. cerevisiae* universal type strain BY4741 in randomised (once) positions on each plate. The plate layout was kept unchanged between duplicates and runs. Runs affected by systematic technical problems were discarded as were isolates severely affected by flocculation (four isolates).

Data analysis: Optical density measurements (OD) were calibrated in the following way:

i) Background correction was achieved by subtracting the SD medium background of 0.067.

ii) Non-linearity of optical density and population density at higher population densities was compensated for by calibration of each OD measurement as: calibrated OD = OD + $0.8324*OD^3$ (PMID: 14676322, PMID: 12489126)

iii) Noise reduction was achieved by a moving average smoothening: each OD measurement, $X_{i,smoothened} = (X_{i-1}+X_i+X_{i+1})/3$.

iv) Confounding effects from stationary phase proliferation curve collapses was compensated for by removing all negative slopes (if $X_{i+1} < X_i$ then set $X_{i+1} = X_i$).

From calibrated proliferation curves, each fitness variable was calculated as:

Proliferation lag: Growth measurements were LN transformed, slopes were calculated between every pair of measurements spaced 140min apart, intercepts between each calculated slope and the extended horizontal line given by the start OD (average of initial five measurements) were calculated and the mean of the two highest intercepts was taken as the length of the lag phase. Proliferation lags longer than 48h were set to 48h.

Proliferation rate: Growth measurements were LN transformed. Slopes were calculated between every pair of measurements spaced 40min apart along the curve (no slopes were calculated from the eight initial time points to filter for digitalisation effects), the top two slopes were discarded to exclude possible artefacts and a mean was calculated from the third to the eight highest slopes. Population doubling time was calculated as LN(2) divided by the mean. Population doubling times longer than 48h were set to 48h.

Proliferation efficiency: The difference between end OD (last measurement) and initial OD (average of initial five measurements) was calculated and taken at face value as a measure of total change in population density. No proliferation efficiency was calculated from growth curves for which no stationary phase was reached, defined as curves with a coefficient of variation over the last five measurements >2%. Proliferation efficiency lower than 0.05 OD units were set to 0.05 OD units.

The calculated fitness variables were transferred onto log scale (LN_2). To normalize fitness variables across plates, runs and instruments, each fitness variable was related to the corresponding measure from four reference strains (BY4741) included on the same plate. Relative fitness measures for each strain and trait, LSC_{ij}, were calculated as:

$$\mathrm{LSC}_{ij} = \frac{\sum_{r=1}^{2}\left[\left[\frac{1}{10}\sum_{k=1}^{10}\log\left(wt_{kj}^{r}\right)\right] - \log\left(x_{ij}^{r}\right)\right]}{2}$$

where wt_{kj} is the fitness variable of the k:th measurement of the wildtype for trait j, x_{ij} is the measure of strain i for trait j and r indicates the run. The LSC for proliferation efficiency was inverted in order to maintain directionality between fitness variables.
