# Supplementary material for: A Geographically Diverse Collection of Schizosaccharomyces pombe Isolates Shows Limited Phenotypic Variation but Extensive Karyotypic Diversity
Source: G3 (Bethesda). 2011 Dec 1;1(7):615–26. doi: 10.1534/g3.111.001123 (PMC3276172; doi:10.1534/g3.111.001123)
Supplement: Supporting Information [file supp_1.7.615_TableS1.pdf]

Table S1 Strains used in this study

Strain number	Source-Collection	Date of isolation	Source	Location isolated
UWOPS 92.229.4	MA Lachance UWOPS	unknown to us	Tequilla	Mexico, Jalisco
UWOPS 94.422.2	MA Lachance UWOPS	unknown to us	Tequilla	Mexico, Jalisco
UFMG 790=CBS10459	Carlos Rosa UFMG	1996	Must of Brazilian cachaça;	Brazil:Vicosa
UFMG R435=CBS10458	Carlos Rosa UFMG	1999	Frozen pulp of Eugenia uniflora;	Brazil: Aracaju
UFMG A1263=CBS10469	Carlos Rosa UFMG	1996	Must of Brazilian cachaça;	Brazil:Vicosa
UFMG A602=CBS10460	Carlos Rosa UFMG	1996	Must of Brazilian cachaça;	Brazil: Belo Horizonte
UFMG R420=CBS10472	Carlos Rosa UFMG	1996	Frozen pulp of Eugenia uniflora	Brazil; Aracaju
UFMG A1153=CBS10468	Carlos Rosa UFMG	1996	Must of Brazilian cachaça;	Brazil: Salinas
UFMG R434=CBS10476	Carlos Rosa UFMG	1999	Frozen pulp of Eugenia uniflora	Brazil: Aracaju
UFMG A826=CBS10465	Carlos Rosa UFMG	1996	Must of Brazilian cachaça;	Brazil: Belo Horizonte
UFMG R416=CBS10470	Carlos Rosa UFMG	1999	Frozen pulp of Eugenia uniflora	Brazil: Aracaju
UFMG R428=CBS10475	Carlos Rosa UFMG	1999	Frozen pulp of Eugenia uniflora	Brazil: Aracaju
UFMG A1152=CBS10467	Carlos Rosa UFMG	1996	Must of Brazilian cachaça;	Brazil: Salinas
UFMG R424=CBS10473	Carlos Rosa UFMG	1999	Frozen pulp of Eugenia uniflora	Brazil: Aracaju
UFMG A571=CBS10463	Carlos Rosa UFMG	1996	Must of Brazilian cachaça;	Brazil: Belo Horizonte
UFMG A529=CBS10462	Carlos Rosa UFMG	1996	Must of Brazilian cachaça;	Brazil: Belo Horizonte
UFMG A1000=CBS10465	Carlos Rosa UFMG	1996	Must of Brazilian cachaça;	Brazil: Belo Horizonte
UFMG R427=CBS10474	Carlos Rosa UFMG	1999	Frozen pulp of Eugenia uniflora	Brazil: Aracaju
UFMG R437=CBS10477	Carlos Rosa UFMG	1999	Frozen pulp of Eugenia uniflora	Brazil: Aracaju
UFMG R418=CBS10471	Carlos Rosa UFMG	1999	Frozen pulp of Eugenia uniflora	Brazil: Aracaju
UFMG A521=CBS10461	Carlos Rosa UFMG	1996	Must of Brazilian cachaça;	Brazil: Belo Horizonte
UFMG A738=CBS10464	Carlos Rosa UFMG	1996	Must of Brazilian cachaça;	Brazil: Belo Horizonte
NCYC 2387=DBVPG 6275 = CBS5557 (T of Schiz. malidevorans)	Steve James NCYC	11/11/1985	Listan grapes	Spain
NCYC 2355-1*	Steve James NCYC	1991	Steve James NCYC	Japan

CBS 356 =DBVPG6277 (T of Schiz. pombe var. pombe)	Steve James NCYC	1922	Arak (aniseed-flavoured distilled alcoholic drink)	Eastern mediterraenen
NCYC 380= CBS 10392	Steve James NCYC	1953	Raw cane sugar	Unknown
NCYC 132= CBS 10391	Steve James NCYC	1921	Millet Beer	
NCYC 936= CBS 10394	Steve James NCYC	1979	Toddy (Palm wine)	Sri Lanka
NCYC 683 = CBS 10393	Steve James NCYC	1966	Fermenting apple juice	Unknown
DBVPG6699-1*	Gianni Litti	01/04/1990	Lychee fruit	Indochina
Y0036	Neil Jolly ARC Infruitec	unknown to us	Wine	South Africa
Y0037	Neil Jolly ARC Infruitec	unknown to us	Wine	South Africa
CBS2628	Steve James NCYC	1952	Palm wine	Pakistan
CBS2775	Steve James NCYC	1957	Fermenting molasses	Japan
CBS5680=DBVPG6448	Steve James NCYC	1965	Apple	Poland
CBS5682=DBVPG6376	Steve James NCYC	1965	Bantu beer	South Africa
CBS7335	Steve James NCYC	1988	Alpechín	Spain
NCYC535 = CBS 4100	Steve James NCYC	1959	Unknown	Unknown
DBVPG2801	Steve James NCYC	1939	Lagby (drink from date Palm)	Tunisia
DBVPG2804	Steve James NCYC	1963	Wine	Malta
DBVPG2805	Steve James NCYC	1963	Wine	Malta
DBVPG2806	Steve James NCYC	1963	Wine	Malta
DBVPG2807	Steve James NCYC	1963	Grape must treated with SO2	Malta
DBVPG2808	Steve James NCYC	1963	Grape must treated with SO2	Malta
DBVPG2809	Steve James NCYC	1963	Grape must treated with SO2	Malta
DBVPG2810	Steve James NCYC	1963	Wine	Malta
DBVPG2811	Steve James NCYC	1966	Grape must treated with SO2	Sicily, Italy
DBVPG2812	Steve James NCYC	1966	Grape must treated with SO2	Sicily, Italy
DBVPG2814	Steve James NCYC	1966	Grape must treated with SO2	Sicily, Italy
DBVPG2815	Steve James NCYC	1966	Grape must treated with SO2	Sicily, Italy
DBVPG2816	Steve James NCYC	1966	Grape must treated with SO2	Sicily, Italy
DBVPG2817	Steve James NCYC	1966	Grape must treated with SO2	Sicily, Italy
DBVPG2818	Steve James NCYC	1966	Grape must treated with SO2	Sicily, Italy
DBVPG4433*	Steve James NCYC	12/04/1996	Unknown	Germany
DBVPG4435	Steve James NCYC	12/04/1996	Unknown	Italy
DBVPG4437	Steve James NCYC	12/04/1996	Unknown	Italy
DBVPG6610	Steve James NCYC	08/08/1986	Lab strain lys1	Unknown
Y468	Gert Marais CAMS	unknown to us	Cape wines	South Africa
Y469	Gert Marais CAMS	unknown to us	Unknown	Unknown
Y470	Gert Marais CAMS	unknown to us	Unknown	Unknown
Y831	Gert Marais CAMS	unknown to us	Industrial glucose;	South Africa
Y832	Gert Marais CAMS	unknown to us	Industrial glucose:	South Africa
CBS374 = DBVPG6418	Gert Marais CAMS	1928	Molasses	Delft
972	Jacky Hayles CRUK	1947	Rotten wine	France
975	Jacky Hayles CRUK	1947	Rotten wine	France

DBVPG 6275 = CBS5557 (T of Sciz maledivorans)	Steve James NCYC	11/11/1985	Listan grapes	Spain
DBVPG6279= CBS1042 (T of Schiz. liquefaciens)	Steve James NCYC	11/11/1985	Sulfited grape juice	Unknown
DBVPG6281=CBS1061	Steve James NCYC	11/11/1985	Cane-sugar molasses	Unknown
DBVPG6417=CBS355	Steve James NCYC	10/16/1987	Cane-sugar molasses	Unknown
DBVPG6447=CBS1043	Steve James NCYC	10/31/1987	Cane-sugar molasses	Unknown
DBVPG 6449=CBS1062	Steve James NCYC	10/31/1987	Cane-sugar molasses	Unknown
CBS358=DPVPG6374	Vincent Robert CBS	1922	Unknown	Unknown
CBS2777	Vincent Robert CBS	1957	Fermenting molasses	Japan
CBS1058	Vincent Robert CBS	1949	Molasses	Java, Indonesia
CBS357=DBVPG6280	Vincent Robert CBS	1912	Cane-sugar molasses	Jamaica
CBS2776	Vincent Robert CBS	1957	Fermenting molasses	Japan
CBS 352= DBVPG 6373	Vincent Robert CBS	1923	Batavian arrak factory	Indonesia
CBS1057= DBVPG6375	Vincent Robert CBS	1933	Brewer's yeast	Skane, Sweden
CBS1059	Vincent Robert CBS	1949	Cane sugar	Mauritius
CBS1044	Vincent Robert CBS	1927	Cane-sugar molasses	Unknown
L2470	Giani Litti	unknown to us	Wine	Chile
AWRI141	Eveline Bartowsky AWRI	25/06/1945	Vineyard	Eden Valley, South Australia
AWRI442	Eveline Bartowsky AWRI	1951	Haslegrove Wines	McLaren Vale, South Australia
CBS1063=DBVPG6450	Vincent Robert CBS	1934	Cane-sugar molasses	Unknown
Y468-subclone WRAB NOTT133				
Y468-subclone WRAB NOTT134				
Y468-subclone WRAB NOTT135				
Y468-subclone WRAB NOTT136				
Y470-subclone WRAB NOTT137				
Y470-subclone WRAB NOTT138				
Y470-subclone WRAB NOTT139				
Y470-subclone WRAB NOTT140				
Y831-subclone WRAB NOTT141				
Y831-subclone WRAB NOTT142				
Y832-subclone WRAB NOTT143				
Y832-subclone WRAB NOTT144				
Y832-subclone WRAB NOTT145				
Y832-subclone WRAB NOTT146				
Y468-subclone WRAB NOTT147				
CBS374-subclone WRAB NOTT148				

* DBVPG 6699 is listed as *Saccharomyces boulardi* however GL found the original isolate to be impure, sub-cloned it and found it to contain a yeast resembling *S. pombe* which we have listed here as DBVPG 6699-1. DBVPG 4433 is listed as *S. pombe* in the DBVPG catalogue which we have confirmed here, however it is also listed as being identical to NCYC 3092 which is listed as *Zygsaccharomyces*. We have not established the source of this discrepancy.
