# Supplementary material for: A Geographically Diverse Collection of Schizosaccharomyces pombe Isolates Shows Limited Phenotypic Variation but Extensive Karyotypic Diversity
Source: G3 (Bethesda). 2011 Dec 1;1(7):615–26. doi: 10.1534/g3.111.001123 (PMC3276172; doi:10.1534/g3.111.001123)
Supplement: Supporting Information [file supp_1.7.615_TableS2.pdf]

Table S2 Assorting 84 strains of *S. pombe* into 40 groups with shared haplotypes.

Strain	hap. no.	660	TER1	TER1+30	TER1+100	CC2L	CC2R	CCC2
UWOPS 92.229.4	**1**	1	1	1	1	1	1	1
**UWOPS 94.422.2**	**2**	2	2	2	1	1	1	1
UFMG A529, UFMG 790, UFMG A826	**3**	3	3	3	1	1	2	1
UFMG R416, UFMG R418, UFMG R420, UFMG R424, UFMG R435	**4**	3	4	1	2	1	1	1
UFMG R427	**5**	3	4	1	2	2	3	3
UFMG A1263	**6**	3	5	3	1	1	2	1
UFMG A521, UFMG A571, UFMG A602	**7**	3	3	3	1	2	1	1
UFMG A1000, UFMG A1153,	**8**	3	6	1	1	2	1	1
UFMG R434	**9**	3	6	1	2	3	1	2
UFMG R428	**10**	3	7	2	2	2	3	1
UFMG A1152	**11**	3	8	1	1	2	1	1
UFMG R437	**12**	3	6	1	2	1	1	1
UFMG A738	**13**	3	3	1	1	1	2	1
NCYC 683, NCYC 2387, DBVPG4435, AWRI 442	**14**	4	6	1	1	1	1	1
NCYC 936, NCYC 2355-1,	**15**	3	9	4	1	4	4	4
**CBS 356, NCYC 132, NCYC535, DBVPG2817, DBVPG4437, AWRI 141**	**16**	5	3	2	1	5	5	5
NCYC 380, CBS 1063, **DBVPG 6281**, CBS 355, **DBVPG 6417**	**17**	3	6	1	2	2	1	1
DBVPG4433, DBVPG 6279, DBVPG6610, DBVPG 6699, Y0036, Y0037, CRUK 972, CRUK 975, Y468, Y469,	**18**	4	6	1	2	2	6	6
CBS 2628	**19**	6	10	4	3	4	7	7
CBS 2775, CBS 2776, **CBS 2777**	**20**	3	11	2	1	1	1	1
CBS 5680	**21**	4	7	2	2	2	1	1
CBS 5682	**22**	7	6	2	1	1	1	1
CBS 7335	**23**	8	12	1	4	1	1	1
DBVPG2801	**24**	3	3	1	5	2	3	8
DBVPG2805	**25**	3	6	1	2	2	6	6
DBVPG2804, DBVPG2806, DBVPG2807, DBVPG2808, DBVPG2809	**26**	4	6	1	1	2	6	6
DBVPG2810	**27**	4	13	5	1	2	6	6
DBVPG2811, DBVPG2812, DBVPG2814, DBVPG2815, DBVPG2816, DBVPG2818	**28**	4	6	1	4	2	6	6
**Y470***	**29**	4	6	5	1	1	6	1
**Y831*, Y832***	**30**	3	6	2	1	2	6	9
CBS374	**31**	3	7	2	2	2	6	6
DBVPG 6447, DBVPG 6449	**32**	3	6	2	1	2	1	1
CBS 358	**33**	3	6	1	1	1	1	1

CBS 1058	**34**	4	6	1	1	2	3	10
CBS 357	**35**	3	14	2	1	1	1	1
CBS 352	**36**	9	15	6	1	1	1	1
CBS 1057	**37**	4	6	1	2	2	1	1
CBS 1059	**38**	4	6	1	2	1	1	1
CBS 1044	**39**	3	16	1	1	2	1	1
L2470	**40**	4	13	5	1	1	1	1

Highlighted in bold are strains that contain translocations detected in the survey. Underlined are strains with the 2.3Mb derived inversion of chromosome I originally identified in CRUK 972. The asterisks indicated strains containing mixtures of karyotypes.

Sequences of haplotypes shown in table S2.

660.16 intron

```
>1
TAAAGCTACAATGATGTTAGCATATTCACATTCGATATGATACAAATAATGTTAAAACAAGGAAAGGTTA
ATGCTCATGAGCCCGGGAAAGCAAGCAAAAAATAAAGACAAAATAATCATAAGTGTGAGTGACAGACAAA
AACAAGATGAACCGAAAACTCCCCGCGAGAGAATCTGATAACGAAATTTTTACCCAACAAACCGGAACGC
CGTGCCATAACAAGTGAGAAAAACTAGGATTGTTTTTCTTACATGCAAAACTCGGACATTTTTGATCACG
ATGCAAAAATAGGGCGCGGGTAAGAAAGAGACAAAAACAAATCGTCGGATAAGTTGATGAGTCATCGTTT
TCGAGGGTCGCTCGTTATCATAGACACATCTACGCTATAACAACACAAAAAGCATGGTAAATGGACAAAA
CCAACACACGCGACTCACCTTACAACGTAGCGCAGTGGAATAACGCAATAGCTATGCAAACGTTATACCA
ACAGAGCAAAAACATTGTAAGAATACACATGCCCGACACAACGGATGGTTGCAAAGAAATCGAGAGTGAA
ACCGCATCGATTACAAACAGGTTTTGAAGGAGCAAACCCGAAAGCAAGAGAAAAACCTTTTCCCATAGCT
ATGTGTTATGCCATATTGAAGAGTAGAAAATGGGAAAGAAATAGCATTTCTAATAAAGAAAGATACTTGT
CTTTGCTTGCTTTCACTTCGATACAAATGTTTTATTATGTCTT

>2
TAAAGCTACAATGATGTTAGCATATTCACATTCGATATGATACAAATAATGTTAAAACAAGGAAAGGTTA
ATGCTCATGAGCCCGGGAAAGCAAGCAAAAAATAAAGACAAAATAATCATAAGTGTGAGTGACAGACAAA
AACAAGATGAACCGAAAACCCCCCGCGAGAGAATCTGATAACGAAATTTTTACCCAACAAACCGGAACGC
CGTGCCATAACAAGTGAGAAAAACTAGGATTGTTTTTCTTACATGCAAAACTCGGACATTTTTGATCACG
ATGCAAAAATAGGGCGCGGGTAAGAAAGAGACAAAAACAAATCGTCGGATAAGTTGATGAGTCATCGTTT
TCGAGGGTCGCTCGTTATCATAGACACATCTACGCTATAACAACACAAAAAGCATGGTAAATGGACAAAA
CCAACACACGCGACTCACCTTACAACGTAGCGCAGTGGAATAACGCAATAGCTATGCAAACGTTATACCA
ACAGAGCAAAAACATTGTAAGAATACACATGCCCGACACAACGGATGGTTGCAAAGAAATCGAGAGTGAA
ACCGCATCGATTACAAACAGGTTTTGAAGGAGCAAACCCGAAAGCAAGAGAAAAACATTTTCCCATAGCT
ATGTGTTATGCCATATTGAAGAGTAGAAAATGGGAAAGAAATAGCATTTCTAATAAAGAAAGAAACCTGT
CTTTGCTTGCTTTCACTTCGATACAAATGTTTTATTATGTCTT

>3
TAAAGCTACAATGATGTTAGCATATTCACATTCGATATGATACAAATAATGTTAAAACAAGGAAAGGTTA
ATGCTCATGAGCCCGGGAAAGCAAGCAAAAAATAAAGACAAAATAATCATAAGTGTGAGTGACAGACAAA
AACAAGATGAACCGAAAACTCCCCGCGAGAGAATCTGATAACGAAATTTTTACCCAACAAACCGGAACGC
CGTGCCATAACAAGTGAGAAAAACTAGGATTGTTTTTCTTACATGCAAAACTCGGACATTTTTGATCACG
ATGCAAAAATAGGGCGCGGGTAAGAAAGAGACAAAAACAAATCGTCGGATAAGTTGATGAGTCATCGTTT
TCGAGGGTCGCTCGTTATCATAGACACATCTACGCTATAACAACACAAAAAGCATGGTAAATGGACAAAA
CCAACACACGCGACTCACCTTACAACGTAGCGCAGTGGAATAACGCAATAGCTATGCAAACGTTATACCA
ACAGAGCAAAAACATTGTAAGAATACACATGCCCGACACAACGGATGGTTGCAAAGAAATCGAGAGTGAA
ACCGCATCGATTACAAACAGGTTTTGAAGGAGCAAACCCGAAAGCAAGAGAAAAACCTTTTCCCATAGCT
ATGTGTTATGCCATATTGAAGAGTAGAAAATGGGAAAGAAATAGCATTTCTAATAAAGAAAGAAACCTGT
CTTTGCTTGCTTTCACTTCGATACAAATGTTTTATTATGTCTT

>4
TAAAGCTACAATGATGTTAGCATATTCACATTCGATATGATACAAATAATGTTAAAACAAGGAAAGGTTA
ATGCTCATGAGCCCGGGAAAGCAAGCAAAAAATAAAGACAAAATAATCATAAGTGTGAGTGACAGACAAA
AACAAGATGAACCGAAAACTCCCCGCGAGAGAATCTGATAACGAAATTTTTACCCAACAAACCGGAACGC
CGTGCCATAACAAGTGAGAAAAACTAGGATTGTTTTTCTTACATGCAAAACTCGGACATTTTTGATCACG
ATGCAAAAATAGGGCGCGGGTAAGAAAGAGACAAAAACAAATCGTCGGATAAGTTGATGAGTCATCGTTT
TCGAGGGTCGCTCGTTATCATAGACACATCTACGCTATAACAACACAAAAAGCATGGTAAATGGACAAAA
CCAACACACGCGACTCACCTTACAACGTAGCGCAGTGGAATAACGCAATAGCTATGCAAACGTTATACCA
ACAGAGCAAAAACATTGTAAGAATACACATGCCCGACACAACGGATGGTTGCAAAGAAATCGAGAGTGAA
ACCGCATCGATTACAAACAGGTTTTGAAGGAGCAAACCCGAAAGCAAGAGAAAAACATTTTCCCATAGCT
ATGTGTTATGCCATATTGAAGAGTAGAAAATGGGAAAGAAATAGCATTTCTAATAAAGAAAGAAACCTGT
CTTTGCTTGCTTTCACTTCGATACAAATGTTTTATTATGTCTT
```

>5

TAAAGCTACAATGATGTTAGCATATTCACATTCGATATGATACAAATAATGTTAAAACAAGGAAAGGTTA
ATGCTCATGAGCCCGGGAAAGCAAGCAAAAAATAAAGACAAAATAATCATAAGTGTGAGTGACAGACAAA
AACAAGATGAACCGAAAACTCCCCGCGAGAAAATCTGATAACGAAATTTTTACCCAACAAACCGGAACGC
CGTGCCATAACAAGTGAGAAAAACTAGGATTGTTTTTCTTACATGCAAAACTCGGACATTTTTGATCACG
ATGCAAAAATAGGGCGCGGGTAAGAAAGAGACAAAAACAAATCGTCGGATAAGTTGATGAGTCATCGTTT
TCGAGGGTCGCTCGTTATCATAGACACATCTACGCTATAACAACACAAAAAGCATGGTAAATGGACAAAA
CCAACACACGCGACTCACCTTACAACGTAGCGCAGTGGAATAACGCAATAGCTATGCAAACGTTATACCA
ACAGAGCAAAAACATTGTAAGAATACACATGCCCGACACAACGGATGGTTGCAAAGAAATCGAGAGTGAA
ACCGCATCGATTACAAACAGGTTTTGAAGGAGCAAACCCGAAAGCAAGAGAAAAACCTTTTCCCATAGCT
ATGTGTTATGCCATATTGAAGAGTAGAAAATGGGAAAGAAATAGCATTTCTAATAAAGAAAGAAACCTGT
CTTTGCTTGCTTTCACTTCGATACAAATGTTTTATTATGTCTT

>6
TAAAGCTACAATGATGTTAGCATATTCACATTCGATATAATACAAATAATGTTAAAACAAGGAAATGTTA
ATGCTCATGAGCCCGGGAAAGCAAGCAAAAAATAAAGACAAAATAATCATAAGTGTGAGTGACAGACAAA
AACAAGATGAACCGAAAACTCCCCGCGAGAGAATCTGATAACGAAATTTTTACCCAACAAACCGGAACGC
CGTGCCATAACAAGTGAGAAAAACTAGGATTGTTTTTCTTACATGCAAAACTCGAACATTTTTGATCACG
ATGCAAAAATAGGGCGCGGGTAAGAAAGAGACAAAAACAAATCGTCGGATAAGTTGATGAGTCATCGTTT
TCGAGGGTCGCTCGTTATCATAGACACATCTACGCTATAACAACACAAAAAGCATGGTAAATGGACAAAA
CCAACACACGCGACTCACCTTACAACGTAGCGCAGTGGAATAACGCAATAGCTATGCAAACGTTATACCA
ACAGAGCAAAAACATTGTAAGAATACACATGCCCGACACAACGGATGGTTGCAAAGAAATCGAGAGTGAA
ACCGCATCGATTACAAACAGGTTTTGAAGGAGCAAACCCGAAAGCAAGAGAAAAACCTTTTCCCATAGCT
ATGTGTTATGCCATATTGAAGAGTAGAAAATGGGAAAGAAATAGCATTTCTAATAAAGAAAGAAACCTGT
CTTTGCTTGCTTTCACTTCGATACAAATGTTTTATTATGTCTT

>7
TAAAGCTACAATGATGTTAGCATATTCACATTCGATATGATACAAATAATGTTAAAACAAGGAAAGGTTA
ATGCTCATGAGCCCGGGAAAGCAAGCAAAAAATAAAGACAAAATAATCATAAGTGTGAGTGACAGACAAA
AACAAGATGAACCGAAAACTCCCCGCGAGAGAATCTGATAACGAAATTTTTACCCAACAAACCGGAATGC
CGTGCCATAACAAGTGAGAAAAGCTAGGATTGTTTTTCTTACATGCAAAACTCGGACATTTTTGATCACG
ATGCAAAAATAGGGCGCGGGTAAGAAAGAGACAAAAACAAATCGTCGGATAAGTTGATGAGTCATCGTTT
TCGAGGGTCGCTCGTTATCATAGACACATCTACGCTATAACAACACAAAAAGCATGGTAAATGGACAAAA
CCAACACACGCGACTCACCTTACAACGTAGCGCAGTGGAATAACGCAATAGCTATGCAAACGTTATACCA
ACAGAGCAAAAACATTGTAAGAATACACATGCCCGACACAACGGATGGTTGCAAAGAAATCGAGAGTGAA
ACCGCATCGATTACAAACAGGTTTTGAAGGAGCAAACCCGAAAGCAAGAGAAAAACCTTTTCCCATAGCT
ATGTGTTATGCCATATCGAAGAGTAGAAAATGGGAAAGAAATAGCATTTCTAATAAAGAAAGAAACCTGT
CTTTGCTTGCTTTCACTTCGATACAAATGTTTTATTATGTCTT

>8
TAAAGCTACAATGATGTTAGCATATTCACATTCGATATGATACAAATAATGTTAAAACAAGGAAAGGTTA
ATGCTCATGAGCCCGGGAAAGCAAGCAAAAAATAAAGACAAAATAATCATAAGTGTGAGTGACAGACAAA
AACAAGATGAACCGAAAACTCCCCGCGAGAGAATCTGATAACGAAATTTTTACCCAACAAACCGGAACGC
CGTGCCATAACAATTGAGAAAAACTAGGATTGTTTTTCTTACATGCAAAACTCGGACATTTTTGATCACG
ATGCAAAAATAGGGCGCGGGTAAGAAAGAGACAAAAACAAATCGTCGGATAAGTTGATGAGTCATCGTTT
TCGAGGGTCGCTCGTTATCATAGACACATCTACGCTATAACAACACAAAAAGCATGGTAAATGGACAAAA
CCAACACACGCGACTCACCTTACAACGTAGCGCAGTGGAATAACGCAATAGCTATGCAAACGTTATACCA
ACAGAGCAAAAACATTGTAAGAATACACATGCCCGACACAACGGATGGTTGCAAAGAAATCGAGAGTGAA
ACCGCATCGATTACAAACAGGTTTTGAAGGAGCAAACCCGAAAGCAAGAGAAAAACATTTTCCCATAGCT
ATGTGTTATGCCATATTGAAGAGTAGAAAATGGGAAAGAAATAGCATTTCTAATAAAGAAAGAAACCTGT
CTTTGCTTGCTTTCACTTCGATACAAATGTTTTATTATGTCTT

>9
TAAAGCTACAATGATGTTAGCATATTCACATTCGATATAATACAAATAATGTTAAAACAAGGAAATGTTA

ATGCTCATGAGCCCGGGAAAGCAAGCAAAAAATAAAGACAAAATAATCATAAGTGTGAGTGACAGACAAA
AACAAGATGAACCGAAAACTCCCCGCGAGAGAATCTGATAACGAAATTTTTACCCAACAAACCGGAACGC
CGTGCCATAACAAGTGAGAAAAACTAGGATTGTTTTTCTTACATGCAAAACTCGAACATTTTTGATCACG
ATGCAAAAATAGGGCGCGGGTAAGAAAGAGACAAAAACAAATCGTCGGATAAGTTGATGAGTCATCGTTT
TCGAGGGTCGCTCGTTATCATAGACACATCTACGCTATAACAACACAAAAAGCATGGTAAATGGACAAAA
CCAACACACGCGACTCACCTTACAACGTAGCGCAGTGGAATAACGCAATAGCTATGCAAACGTTATACCA
ACAGAGCAAAAACATTGTAAGAATACACATGCCCGACACAACGGATGGTTGCAAAGAAATCGAGAGTGAA
ACCGCATCGATTACAAACAGGTTTTGAAGGAGCAAACCCGAAAGCAAGAGAAAAACCTTTTCCCATAGCT
ATGTGTTATGCCATATTGAAGAGTAGAAAATGGGAAAGAAATAGCATTTCTAATAAAGAAAGAGACCTGT
CTTTGCTTGCTTTCACTTCGATACAAATGTTTTATTATGTCTT

TER1

>1
ACAGATACAATTAAGAATAACTTGTAATGGTATTCACATTACTAATAAACTTCTTAATTCGTCTGTTTGG
GTATTCATTACATATTCATCAACTAAGCAAGTCTTACATGCGTCCAAATTTGTTTAAGACAAGAAAATAT
ACCATCTATGCTTAATAAACAAACTTCATCTCTTCTAGTACGCAAATAAATACATTAAATTTATTTTACA
TTATAATAAGATTCATCACTTTCTCAAAATTTTGAAACCGGAAACGGAATTCAGCATGTTTTAATAAAAA
GATGAGCAACCGCGTTCTTACTAAATACGCAATTCTAATAATTTGGGAATTGTAGTTAGTAATTTTAAAT
TTAATCATTAAAACATACCAAAAAATGGGCCCGTTCCTTTTAACATTTTGCTCAGACCAAGTGAAAAAAA
CCTAAAACGCGCTCAAGAGGAACAACGGACGAGCTACACTCAAAATATTAATGAATGGGTAGTCAACTAT
TTTACTGGCGAACAAGCAAAAGCTTTGATCCATGGATCTCACGTAATGTAAAATAGTATCTTCCCTAAGC
ATTATTTATTTGAGAAACAGGACGTTCATTATTGTTTGCCGCAAAGAATCCAAGTTCAAGGAAATTGAAT
ACAGACAAGAGTTGCTATTCGTTTGAATGTACATTGTGACCACAAGTCGGTTTTTGTTTCCTTCCAAATA
GTACCTTCAACACGAAGTTGTACATTTAGCACGGACAAAGAATCGACCAAAAAACCAAAATTAAATGTAG
AAGGAAAACCTTCAAGTCATTTTTTTTATAATAGCTCTTTTAAGTTCCATACTTTTTATCTCCGTAACAA
ATGCCAAAAGAAGCTAGTATAGACGAGAGCATTCAGCCAACAAATTTGCACAAGCAAAAATTGTTACCAA
TACAATTTAATATTTGTATGGGGAATTGAAACTATCTATAAAACTTTCTTCATTGCTTGATTTTTAGTAA
CCTTCAGTAATCAAACGAGACTCTTCTACCAAAAATTCGTTGTGATCTGACAAGCACTTAAATGATGGAT
ACAGAATTTATACAAAAGAAAGGTAGGATTAAACTGTAAATGAACTTTAAAGGCAGAAGACTCACGTACA
CTACACCCAGCGATTCAATTGATGTCCAGGCCTCTTTTTTCCTACTGTATACAGAAGTATGAAGTCTGCA
GCCATAATAAAAGCGGTAGGAATGATCTATGTTTTCCGCAACGCGTCAACGATTCCATGAAGAGCCTCTC
TTATGAATGTTCCTAATGATCAAAACTCTAAAAAAAGCTAAAAAGAATAATTTCGCAATGCTGAAAAGTT
GAAGAGTACGGTTACACGAAGCAGTACAGGAATTCGAAATCCATCGAAAACAACTATCCAGACAAACTGA
AAAGCTAAACAGACTACTCGTTCTTTCTAGTAGACAGTATACAGGTTTTGGAATAGCAAACTATACTTAA
GGCCTATGAATCATTTCCTTCAAACTTTCAAATCAATCACCAAGTCCTCAAGAACCGTTTGATTAATTTT
CCTTGTCAACCTTCTAAGCATGGGCGTTGCGTTGAGTATAAACAAAACAGCAATAAATCCGAGTATACAT
GAATTGTTTCCTTCTTCAAACAAAACGCTGTTACCTTTGCGCGTATGATGACTTCCCATACTTACAAAAA
TTTTCAAAACCTAATA

>2
ACAGATACAATTAAGAATAACTTGTAATGGTATTCACATTACTAATAAACTTCTTAATTCATCTGTTTGG
GTATTCATTACATATTCATCAACTAAGCAAGTCTTACATGCGTCCAAATTTGTTTAAGACAAGAAAATAT
ACCATCTATGCTTAATAAACAAACTTCATCTCTTCTAGTACGCAAATAAATACATTAAATTTATTTTACA
TTATAATAAGATTCATCACTTTCTCAAAATTTTGAAACCGGAAACGGAATTCAGCATGTTTTAATAAAAA
GATGAGCAACCGCGTTCTTACTAAATACGCAATTCTAATAATTTGGGAATTGTAGTTAGTAATTTTAAAT
TTAATCATTAAAACATACCAAAAAATGGGCCCGTTCCTTTTAACATTTTGCTCAGACCAAGTGAAAAAAA
CCTAAAACGCGCTCAAGAGGAACAACGGACGAGCTACACTCAAAATATTAATGAATGGGTAGTCAACTAT
TTTACTAGCGAACAAGCAAAAGCTTTGATCCATGGATCTCACGTAATGTAAAATAGTATCTTCCCTAAGC
ATTAATTATTTGAGAAACAGGACGTTCATTATTGTTTGCCGCAAAGAATCCAAGTTCAAGGAAATTGAAT
ACAGACAAGAGTTGCTATTCGTTTGAATGTACATTGTGACCACAAGTCGGTTTTTGTTTCCTTCCAAATA
GTACCTTCAACACGAAGTTGTACATTTAGCACGGACAAAGAATCGACCAAAAAACCAAAATTAAATGTAG
AAGGAAAACCTTCAAGTCATTTTTTTTATAATAGCTCTTTTAAGTTCCATACTTTTTATCTCCGTAACAA
ATGCCAAAAGAAGCTAGTATAGACGAGAGCATTCAGCCAACAAATTTGCACAAGCAAAAATTGTTACCAA
TACAATTTAATATTTGTATGGGGAATTGAAACTATCTATAAAACTTTCTTCATTGCTTGATTTTTAGTAA
CCTTCAGTAATCAAACGAGACTCTTCTACCAAAAATTCGTTGTGATCTGACAAGCACTTAAATGATGGAT
ACAGAATTTATACAAAAGAAAGGTAGGATTAAACTGTAAATGAACTTTAAAGGCAGAAGACTCACGTACA

CTACACCCAGCGATTCAATTGATGTCCAGGCCTCTTTTTTCCTACTGTATACAGAAGTATGAAGTCTGCA
GCCATAATAAAAGCGGTAGGAATGATCTATGTTTTCCGCAACGCGTCAACGATTCCATGAAGAGCCTCTC
TTATGAATGTTCCTAATGATCAAAACTCTAAAAAAAGCTAAAAAGAATAATTTCGCAATGCTGAAAAGTT
GAAGAGTACGGTTACACGAAGCAGTACAGGAATTCGAAATCCATCGAAAACAACTATCCAGACAAACTGA
AAAGCTAAACAGACTACTCGTTCTTTCTAGTAGACAGTATACAGGTTTTGGAATAGCAAACTATACTTAA
GGCCTATGAATCATTTCCTTCAAACTTTCAAATCAATCACCAAGTCCTCAAGAACCGTTTGATTAATTTT
CCTTGTCAACCTTCTAAGCATGGGCGTTGCGTTGAGTATAAACAAAACAGCAATAAATCCGAGTATACAT
GAATTGTTTCCTTCTTCAAACAAAACGCTGTTACCTTTGCGCGTATGATGACTTCCCATACTTACAAAAA
TTTTCAAAACCTAATA

>3
ACAGATACAATTAAGAATAACTTGTAATGGTATTCACATTACTAATAAACTTCTAAATTCATCTGTTTGG
GTATTCATTACATATTCATCAACTAAGCAAGTCTTACATGCGTCCAAATTTGTTTAAGACAAGAAAATAT
ACCATCTATGCTTAATAAACAAACTTCATCTCTTCTAGTACGCAAATAAATACATTAAATTTATTTTACA
TTATAATAAGATTCATCACTTTCTCAAAATTTTGAAACCGGAAACGGAATTCAGCATGTTTTAATAAAAA
GATGAGCAACCGCGTTCTTACTAAATACGCAATTCTAATAATTTGGGAATTGTAGTTAGTAATTTTAAAT
TTAATCATTAAAACATACCAAAAAATGGGCCCGTTCCTTTTAACATTTTGCTCAGACCAAGTGAAAAAAA
CCTAAAACGCGCTCAAGAGGAACAACGGACGAGCTACACTCAAAATATTAATGAATGGGTAGTCAACTAT
TTTACTAGCGAACAAGCAAAAGCTTTGATCCATGGATCTCACGTAATGTAAAATAGTATCTTCCCTAAGC
ATTATTTATTTGAGAAACAGGACGTTCATTATTGTTTGCCGCAAAGAATCCAAGTTCAAGGAAATTGAAT
ACAGACAAGAGTTGCTATTCGTTTGAATGTACATTGTGACCACAAGTCGGTTTTTGTTTCCTTCCAAATA
GTACCTTCAACACGAAGTTGTACATTTAGCACGGACAAAGAATCGACCAAAAAACCAAAATTAAATGTAG
AAGGAAAACCTTCAAGTCATTTTTTTTATAATAGCTCTTTTAAGTTCCATACTTTTTATCTCCGTAACAA
ATGCCAAAAGAAGCTAGTATAGACGAGAGCATTCAGCCAACAAATTTGCACAAGCAAAAATTGTTACCAA
TACAATTTAATATTTGTATGGGGAATTGAAACTATCTATAAAACTTTCTTCATTGCTTGATTTTTAGTAA
CCTTCAGTAATCAAACGAGACTCTTCTACCAAAAATTCGTTGTGATCTGACAAGCACTTAAATGATGGAT
ACAGAATTTATACAAATGAAAGGTAGGATTAAACTGTAAATGAACTTTAAAGGCAGAAGACTCACGTACA
CTACACCCAGCGATTCAATTGATGTCCAGGCCTCTTTTTTCCTACTGTATACAGAAGTATGAAGTCTGCA
GCCATAATAAAAGCGGTAGGAATGATCTATGTTTTCCGCAACGCGTCAACGATTCCATGAAGAGCCTCTC
TTATGAATGTTCCTAATGATCAAAACTCTAAAAAAAGCTAAAAAGAATAATTTCGCAATGCTGAAAAGTT
GAAGAGTACGGTTACACGAAGCAGTACAGGAATTCGAAATCCATCGAAAACAACTATCCAGACAAACTGA
AAAGCTAAACAGACTACTCGTTCTTTCTAGTAGACAGTATACAGGTTTTGGAATAGCAAACTATACTTAA
GGCCTATGAATCATTTCCTTCAAACTTTCAAATCAATCACCAAGTCCTCAAGAACCGTTTGATTAATTTT
CCTTGTCAACCTTCTAAGCATGGGCGTTGCGTTGAGTATAAACAAAACAGCAATAAATCCGAGTATACAT
GAATTGTTTCCTTCTTCAAACAAAACGCTGTTACCTTTGCGCGTATGATGACTTCCCATACATACAAAAA
TTTTCAAAACCTAATA

>4
ACAGATACAATTAAGAATAACTTGTAATGGTATTCACATTACTAATAAACTTCTAAATTCATCTGTTTGG
GTATTCATTACATATTCATCAACTAAGCAAGTCTTACATGCGTCCAAATTTGTTTAAGACAAGAAAATAT
ACCATCTATGCTTAATAAACAAACTTCATCTCTTCTAGTACGCAAATAAATACATTAAATTTATTTTACA
TTATAATAAGATTCATCACTTTCTCAAAATTTTGAAACCGGAAACGGAATTCAGCATGTTTTAATAAAAA
GATGAGCAACCGCGTTCTTACTAAATACGCAATTCTGATAATTTGGGAATTGTAGTTAGTAATTTTAAAT
TTAATCATTAAAACATACCAAAAAATGGGCCCGTTCCTTTTAACATTTTGCTCAGACCAAGTGAAAAAAA
CCTAAAACGCGCTCAAGAGGAACAACGGACGAGCTACACTCAAAATATTAATGAATGGGTAGTCAACTAT
TTTACTAGCGAACAAGCAAAAGCTTTGATCCATGGATCTCACGTAATGTAAAATAGTATCTTCCCTAAGC
ATTATTTTTTTGAGAAACAGGACGTTCATTATTGTTTGCCGCAAAGAATCCAAGTTCAAGGAAATTGAAT
ACAGACCAGAGTTGCTATTCGTTTGAATGTACATTGTGACCACAAGTCGGTTTTTGTTTCCTTCCAAATA
GTACCTTCAACACGAAGTTGTACATTTAGCACGGACAAAGAATCGACCAAAAAACCAAAATTAAATGTAG
AAGGAAAACCTTCAAGTCATTTTTTTTATAATAGCTCTTTTAAGTTCCATACTTTTTATCTCCGTAACAA
ATGCCAAAAGAAGCTAGTATAGACGAGAGCATTCAGCCAACAAATTTGCACAAGCAAAAATTGTTACCAA
TACAATTTAATATTTGTATGGGGAATTGAAACTATCTATAAAACATTCTTCATTGCTTGATTTTTAGTAA
CGTTCAGTAATCAAACGAGACTCTTCTACCAAAAATTCGTTGTGATCTGACAAGCACTTAAATGATGGAT
ACAGATTTTATACAAATGAAAGGTAGGATTAAACTGTAAATGAATTTTAAAGGCAGAAGACTCACGTACA
CTGCACCCAGCGATTCAATTGATGTCCAGGCCTCTTTTTTCCTACTGTATACAGAAGTATGAAGTCTGCA
GCCATAATAAAAGCGGTAGGAATGATCTATGTTTTCCGCAACGCGTCAACGATTCCATGAAGAGCCTCTC
TTATGAATATTCCTAATGATCAAAACTCTAAAAAAAGCTAAAAAGAATAATTTCGCAATGCTGAAAAGTT
GAAGAGTACGGTTACACGAAGCAGTACAGGAATTCGAAATCCATCGAAAACAACTATCCAGACAAACTGA

AAAGCTAAACAGACTACTCGTTCTTTCTAGTAGACAGTATACAGGTTTTGGAATAGCAAACTATACTTAA
GGCCTATGAATCATTTCCTTCAAACTTTCAAATCAATCACCAAGCCCTCAAGAACCGTTTGATTAATTTT
CCTTGTCAACCTTCTAAGCATGGGCGTTGCGTTGAGTATAAACAAAACAGCAATAAATCCGAGTATACAT
GAATTGTTTCCTTCTTCAAACAAAACGCTGTTACCTTTGCGCGTATGATGACTTCCCATACTTACAAAAA
TTTTCAAAACCTAACA

>5
ACAGATACAATTAAGAATAACTTGTAATGGTATTCACATTACTAATAAACTTCTAAATTCATCTGTTTGG
GTATTCATTACATATTCATCAACTAAGCAAGTCTTACATGCGTCCAAATTTGTTTAAGACAAGAAAATAT
ACCATCTATGCTTAATAAACAAACTTCATCTCTTCTAGTACGCAAATAAATACATTAAATTTATTTTACA
TTATAATAAGATTCATCACTTTCTCAAAATTTTGAAACCGGAAACGGAATTCAGCATGTTTTAATAAAAA
GATGAGCAACCGCGTTCTTACTAAATACGCAATTCTGATAATTTGGGAATTGTAGTTAGTAATTTTAAAT
TTAATCATTAAAACATACCAAAAAATGGGCCCGTTCCTTTTAACATTTTGCTCAGACCAAGTGAAAAAAA
CCTAAAACGCGCTCAAGAGGAACAACGGACGAGCTACACTCAAAATATTAATGAATGGGTAGTCAACTAT
TTTACTAGCGAACAAGCAAAAGCTTTGATCCATGGATCTCACGTAATGTAAAATAGTATCTTCCCTAAGC
ATTATTTTTTTGAGAAACAGGACGTTCATTATTGTTTGCCGCAAAGAATCCAAGTTCAAGGAAATTGAAT
ACAGACCAGAGTTGCTATTCGTTTGAATGTACATTGTGACCACAAGTCGGTTTTTGTTTCCTTCCAAATA
GTACCTTCAACACGAAGTTGTACATTTAGCACGGACAAAGAATCGACCAAAAAACCAAAATTAAATGTAG
AAGGAAAACCTTCAAGTCATTTTTTTTATAATAGCTCTTTTAAGTTCCATACTTTTTATCTCCGTAACAA
ATGCCAAAAGAAGCTAGTATAGACGAGAGCATTCAGCCAACAAATTTGCACAAGCAAAAATTGTTACCAA
TACAATTTAATATTTGTATGGGGAATTGAAACTATCTATAAAACTTTCTTCATTGCTTGATTTTTAGTAA
CGTTCAGTAATCAAACGAGACTCTTCTACCAAAAATTCGTTGTGATCTGACAAGCACTTAAATGATGGAT
ACAGATTTTATACAAATGAAAGGTAGGATTAAACTGTAAATGAATTTTAAAGGCAGAAGACTCACGTACA
CTGCACCCAGCGATTCAATTGATGTCCAGGCCTCTTTTTTCCTACTGTATACAGAAGTATGAAGTCTGCA
GCCATAATAAAAGCGGTAGGAATGATCTATGTTTTCCGCAACGCGTCAACGATTCCATGAAGAGCCTCTC
TTATGAATATTCCTAATGATCAAAACTCTAAAAAAAGCTAAAAAGAATAATTTCGCAATGCTGAAAAGTT
GAAGAGTACGGTTACACGAAGCAGTACAGGAATTCGAAATCCATCGAAAACAACTATCCAGACAAACTGA
AAAGCTAAACAGACTACTCGTTCTTTCTAGTAGACAGTATACAGGTTTTGGAATAGCAAACTATACTTAA
GGCCTATGAATCATTTCCTTCAAACTTTCAAATCAATCACCAAGCCCTCAAGAACCGTTTGATTAATTTT
CCTTGTCAACCTTCTAAGCATGGGCGTTGCGTTGAGTATAAACAAAACAGCAATAAATCCGAGTATACAT
GAATTGTTTCCTTCTTCAAACAAAACGCTGTTACCTTTGCGCGTATGATGACTTCCCATACTTACAAAAA
TTTTCAAAACCTAACA

>6
ACAGATACAATTAAGAATAACTTGTAATGGTATTCACATTACTAATAAACTTCTAAATTCATCTGTTTGG
GTATTCATTACATATTCATCAACTAAGCAAGTCTTACATGCGTCCAAATTTGTTTAAGACAAGAAAATAT
ACCATCTATGCTTAATAAACAAACTTCATCTCTTCTAGTACGCAAATAAATACATTAAATTTATTTTACA
TTATAATAAGATTCATCACTTTCTCAAAATTTTGAAACCGGAAACGGAATTCAGCATGTTTTAATAAAAA
GATGAGCAACCGCGTTCTTACTAAATACGCAATTCTAATAATTTGGGAATTGTAGTTAGTAATTTTAAAT
TTAATCATTAAAACATACCAAAAAATGGGCCCGTTCCTTTTAACATTTTGCTCAGACCAAGTGAAAAAAA
CCTAAAACGCGCTCAAGAGGAACAACGGACGAGCTACACTCAAAATATTAATGAATGGGTACTCAACTAT
TTTACTAGCGAACAAGCAAAAGCTTTGATCCATGGATCTCACGTAATGTAAAATAGTATCTTCCCTAAGC
ATTATTTATTTGAGAAACAGGACGTTCATTATTGTTTGCCGCAAAGAATCCAAGTTCAAGGAAATTGAAT
ACAGACAAGAGTTGCTATTCGTTTGAATGTACATTGTGACCACAAGTCGGTTTTTGTTTCCTTCCAAATA
GTACCTTCAACACGAAGTTGTACATTTAGCACGGACAAAGAATCGACCAAAAAACCAAAATTAAATGTAG
AAGGAAAACCTTCAAGTCATTTTTTTTATAATAGCTCTTTTAAGTTCCATACTTTTTATCTCCGTAACAA
ATGCCAAAAGAAGCTAGTATAGACGAGAGCATTCAGCCAACAAATTTGCACAAGCAAAAATTGTTACCAA
TACAATTTAATATTTGTATGGGGAATTGAAACTATCTATAAAACTTTCTTCATTGCTTGATTTTTAGTAA
CCTTCAGTAATCAAACGAGACTCTTCTACCAAGAATTCGTTGTGATCTGACAAGCACTTAAATGATGGAT
ACAGAATTTATACAAATGAAAGGTAGGATTAAACTGTAAATGAACTTTAAAGGCAGAAGACTCACGTACA
CTACACCCAGCGATTCAATTGATGTCCAGGCCTCTTTTTTCCTACTGTATACAGAAGTATGAAGTCTGCA
GCCATAATAAAAGCGGTAGGAATGATCTATGTTTTCCGCAACGCGTCAACGATTCCATGAAGAGCCTCTC
TTATGAATGTTCCTAATGATCAAAACTCTAAAAAAAGCTAAAAAGAATAATTTCGCAATGCTGAAAAGTT
GAAGAGTACGGTTACACGAAGCAGTACAGGAATTCGAAATCCATCGAAAACAACTATCCAGACAAACTGA
AAAGCTAAACAGACTACTCGTTCTTTCTAGTAGACAGTATACAGGTTTTGGAATAGCAAACTATACTTAA
GGCCTATGAATCATTTCCTTCAAACTTTCAAATCAATCACCAAGTCCTCAAGAACCGTTTGATTAATTTT
CCTTGTCAACCTTCTAAGCATGGGCGTTGCGTTGAGTATAAACAAAACAGCAATAAATCCGAGTATACAT
GAATTGTTTCCTTCTTCAAACAAAACGCTGTTACCTTTGCGCGTATGATGACTTCCCATACATACAAAAA

TTTTCAAAACCTAATA

>7
ACAGATACAATTAAGAATAACTTGTAATGGTATTCACATTACTAATAAACTTCTAAATTCATCTGTTTGG
GTATTCATTACATATTCATCAACTAAGCAAGTCTTACATGCGTCCAAATTTGCTTAAGACAAGAAAATAT
ACCATCTATGCTTAATAAACAAACTTCATCTCTTCTAGTACGCAAATAAATACATTAAATTTATTTTACA
TTATAATAAGATTCATCACTTTCTCAAAATTTTGAAACCGGAAACGGAATTCAGCATGTTTTAATAAAAA
GATGAGCAACCGCGTTCGTACTAAATACGCAATTCTAATAATTTGGGAATTGTAGTTAGTAATTTTAAAT
TTAATCATTAAAACATACCAAAAAATGGGCCCGTTCCTTTTAACATTTTGCTCAGACCAAGTGAAAAAAA
CCTAAAACGCGCTCAAGAGGAACAACGGACGAGCTACACTCAAAATATTAATGAATGGGTAGTCAACTAT
TTTACTAGCGAACAAGCAAAAGCTTTGATCCATGGATCTCACGTAATGTAAAATAGTATCTTCCCTAAGC
ATTATTTATTTGAGAAACAGGACGTTCATTATTGTTTGCCGCAAAGAATCCAAGTTCAAGGAAATTGAAT
ACAGACAAGAGTTGCTATTCGTTTGAATGTACATTGTGACCACAAGTCGGTTTTTGTTTCCTTCCAAATA
GTACCTTCAACACGAAGTTGTACATTTAGCACGGACAAAGAATCGACCAAAAAACCAAAATTAAATGTAG
AAGGAAAACCTTCAAGTCATTTTTTTTATAATAGCTCTTTTAAGTTCCATACTTTTTATCTCCGTAACAA
ATGCCAAAAGAAGCTAGTATAGACGAGAGCATTCAGCCAACAAATTTGCACAAGCAAAAATCGTTACCAA
TACAATTTAATATTTGTATGGGGAATTGAAACTATCTATAAAACTTTCTTCATTGCTTGATTTTTAGTAA
CCTTCAGTAATCAAACAAGACTCTTCTACCAAAAATTCGTTGTGATCTGACAAGCACTTAAATGATGGAT
ACAGAATTTATACAAATGAAAGGTAGGATTAAACTGTAAATGAATTTTAAAGGCAGAAGACTCACGTACA
CTACACCCAGCGATTCAATTGATGTCCAGGCCTCTTTTTCCCTACTGTATACAGAAGTATGAAGTCTGCA
GCCATAATAAAAGCGGTAGGAATGATCTATGTTTTCCGCAACGCGTCAACGATTCCATGAAGAGCCTCTC
TTATGAATGTTCCTAATGATCAAAACTCTAAAAAAAGCTAAAAAGAATAATTTCGCAATGCTGAGAAGTT
GAAGAGTACGGTTACACGAAGCAGTACAGGAATTCGAAATCCATCGAAAACAACTATCCAGACAAACTGA
AAAGCTAAACAGACTACTCGTTCTTTCTAGTAGACAGTATACAGGTTTTGGAATAGCAAACTATACTTAA
GGCCTATGAATCATTTCCTTCAAACTTTCAAATCAATCACCAAGCCCTCAAGAACCGTTTGATTAATTTT
CCTTGTCAACCTTCTAAGCATGGGCGTTGCGTTGAGTATAAACAAAACAGCAATAAATCCGAGTATACAT
GAATTGTTTCCTTCTTCAAACAAAACGCTGTTACCTTTGCGCGTATGATGACTTCCCATACTTACAAAAA
TTTTCAAAACCTAATA

>8
ACAGATACAATTAAGAATAACTTGTAATGGTATTCACATTACTAATAAACTTCTAAATTCATCTGTTTGG
GTATTCATTACATATTCATCAACTAAGCAAGTCTTACATGCGTCCAAATTTGTTTGAGACAAGAAAATAT
ACCATCTATGCTTAATAAACAAACTTCATCTCTTCTAGTACGCAAATAAATACATTAAATTTATTTTACA
TTATAATAAGATTCATCACTTTCTCAAAATTTTGAAACCGGAAACGGAATTCAGCATGTTTTAATAAAAA
GATGAGCAACCGCGTTCGTACTAAATACGCAATTCTAATAATTTGGGAATTGTAGTTAGTAATTTTAAAT
TTAATCATTAAAACATACCAAAAAATGGGCCCGTTCCTTTTAACATTTTGCTCAGACCAAGTGAAAAAAA
CCTAAAACGCGCTCAAGAGGAACAACGGACGAGCTACACTCAAAATATTAATGAATGGGTAGTCAATTAT
TTTACTAGCGAACAAGCAAAAGCTTTGATCCATGGATCTCACGTAATGTAAAATAGTATCTTCCCTAAGC
ATTATTTATTTGAGAAACAGGACGTTCATTATTGTTTGCCGCAAAGAATCCAAGTTCAAGGAAATTGAAT
ACAGACAAGAGTTGCTATTCGTTTGAATGTACATTGTGACCACAAGTCGGTTTTTGTTTCCTTCCAAATA
GTACCTTCAACACGAAGTTGTACATTTAGCACGGACAAAGAATCGACCAAAAAACCAAAATTAAATGTAG
AAGGAAAACCTTCAAGTCATTTTTTTTATAATAGCTCTTTTAAGTTCCATACTTTTTATCTCCGTAACAA
ATGCCAAAAGAAGCTAGTATAGACGAGAGCATTCAGCCAACAAATTTGCACAAGCAAAAATCGTTACCAA
TACAATTTAATATTTGTATGGGGAATTGAAACTATCTATAAAACTTTCTTCATTGCTTGATTTTTAGTAA
CCTTCAGTAATCAAACAAGACTCTTCTACCAAAAATTCGTTGTGATCTGACAAGCACTTAAATGATGGAT
ACAGAATTTATACAAATGAAAGGTAGGATTAAACTGTAAATGAATTTTAAAGGCAGAAGACTCACGTACA
CTACACCCAGCGATTCAATTGATGTCCAGGCCTCTTTTTCCCTACTGTATACAGAAGTATGAAGTCTGCA
GCCATAATAAAAGCGGTAGGAATGATCTATGTTTTCCGCAACGCGTCAACGATTCCATGAAGAGCCTCTC
TTATGAATGTTCCTAATGATCAAAACTCTAAAAAAAGCTAAAAAGAATAATTTCGCAATGCTGAGAAGTT
GAAGAGTACGGTTACACGAAGCAGTACAGGAATTCGAAATCCATCGAAAACAACTATCCAGACAAACTGA
AAAGCTAAACAGACTACTCGTTCTTTCTAGTAGACAGTATACAGGTTTTGGAATAGCAAACTATACTTAA
GGCCTATGAATCATTTCCTTCAAACTTTCAAATCAATCACCAAGCCCTCAAGAACCGTTTGATTAATTTT
CCTTGTCAACCTTCTAAGCATGGGCGTTGCGTTGAGTATAAACAAAACAGCAATAAATCCGAGTATACAT
GAATTGTTTCCTTCTTCAAACAAAACGCTGTTACCTTTGCGCGTATGATGACTTCCCATACTTACAAAAA
TTTTCAAAACCTAATA

>9
ACAGATACAATTAAGAATAACTTGTAATGGTATTCACATTACTAATAAACTTCTAAATTCATCTGTTTGG

GTATTCATTACATATTCATCAACTAAGCAAGTCTTACATGCGTCCAAATTTGTTTAAGACAAGAAAATAT
ACCATCTATGCTTAATAAACAAACTTCATCTCTTCTAGTACGCAAATAAATACATTAAATTTATTTTACA
TTATAATAAGATTCATCACTTTCTCAAAATTTTGAAACCGGAAACGGAATTCAGCATGTTTTAATAAAAA
GATGAGCAACCGCGTTCTTACTAAATACGCAATTCTGATAATTTGGGAATTGTAGTTAGTAATTTTAAAT
TTAATCATTAAAACATACCAAAAAATGGGCCCGTTCCTTTTAACATTTTGCTCAGACCAAGTGAAAAAAA
CCTAAAACGCGCTCAAGAGGAACAACGGACGAGCTACACTCAAAATATTAATGAATGGGTAGTCAACTAT
TTTACTAGCGAACAAGCAAAAGCTTTGATCCATGGATCTCACGTAATGTAAAATAGTATCTTCCCTAAGC
ATTATTTATTTGAGAAACAGGACGTTCATTATTGTTTGCCGCAAAGAATCCAAGTTCAAGGAAATTGAAT
ACAGACAAGAGTTGCTATTCGTTTGAATGTACATTGTGACCACAAGTCGGTTTTTGTTTCCTTCCAAATA
GTACCTTCAACACGAAGTTGTACATTTAGCACGGACAAAGAATCGACCAAAAAACCAAAATTAAATGTAG
AAGGAAAACCTTCAAGTCATTTTTTTTATAATAGCTCTTTTAAGTTCCATACTTTTTATCTCCGTAACAA
ATGCCAAAAGAAGCTAGTATAGACGAGAGCATTCAGCCAACAAATTTGCACAAGCAAAAATTGTTACCAA
TACAATTTAATATTTGTATGGGGAATTGAAACTATCTATAAAACTTTCTTCATTGCTTGATTTTTAGTAA
CCTTCAGTAATCAAACGAGACTCTTCTACCAAAAATTCGTTGTGATCTGACAAGCACTTAAATGATGGAT
ACAGAATTTATACAAATGAAAGGTAGGATTAAACTGTAAATGAACTTTAAAGGCAGAAGACTCACGTACA
CTACACCCAGCGATTCAATTGATGTCCAGGCCTCTTTTTTCCTACTGTATACAGAAGTATGAAGTCTGCA
GCCATAATAAAAGCGGTAGGAATGATCTATGTTTTCCGCAACGCGTCAACGATTCCATGAAGAGCCTCTC
TTATGAATGTTCCTAATGATCAAAACTCTAAAAAAAGCTAAAAAGAATAATTTCGCAATGCTGAAAAGTT
GAAGAGTACGGTTACACGAAGCAGTACAGGAATTCGAAATCCATCGAAAACAACTATCCAGACAAACTGA
AAAGCTAAACAGACTACTCGTTCTTTCTAGTAGACAGTATACAGGTTTTGGAATAGCAAACTATACTTAA
GGCCTATGAATCATTTCCTTCAAACTTTCAAATCAATCACCAAGTCCTCAAGAACCGTTTGATTAATTTT
CCTTGTCAACCTTCTAAGCATGGGCGTTGCGTTGAGTATAAACAAAACAGCAATAAATCCGAGTATACAT
GAATTGTTTCCTTCTTCAAACAAAACGCTGTTACCTTTGCGCGTATGATGACTTCCCATACATACAAAAA
TTTTCAAAACCTAATA

>10
ACAGATACAATTAAGAATAACTTGTAATGGTATTCACATTACTAATAAACTTCTAAATTCATCTGTTTGG
GTATTCATTACATATTCATCAACTAAGCAAGTCTTACATGCGTCCAAATTTGTTTAAGACAAGAAAATAT
ACCATCTATGCTTAATAAACAAACTTCATCTCTTCTAGTACGCAAATAAATACATTAAATTTATTTTACA
TTATAATAAGATTCATCACTTTCTCAAAATTTTGAAACCGGAAACGGAATTCAGCATGTTTTAATAAAAA
GATGAGCAACCGCGTTCTTACTAAATACGCAATTCTGATAATTTGGGAATTGTAGTTAGTAATTTTAAAT
TTAATCATTAAAACATACCAAAAAATGGGCCCGTTCCTTTTAACATTTTGCTCAGACCAAGTGAAAAAAA
CCTAAAACGCGCTCAAGAGGAACAACGGACGAGCTACACTCAAAATATTAATGAATGGGTAGTCAACTAT
TTTACTAGCGAACAAGCAAAAGCTTTGATCCACGGATCTCACGTAATGTAAAATAGTATCTTCCCTAAGC
ATTATTTTTTTGAGAAACAGGACGTTCATTATTGTTTGCCGCAAAGAATCCAAGTTCAAGGAAATTGAAT
ACAGACCAGAGTTGCTATTCGTTTGAATGTACATTGTGACCACAAGTCGGTTTTTGTTTCCTTCCAAATA
GTACCTTCAACACGAAGTTGTACATTTAGCACGGACAAAGAATCGACCAAAAAACCAAAATTAAATGTAG
AAGGAAAACCTTCAAGTCATTTTTTTTATAATAGCTCTTTTAAGTTCCATACTTTTTATCTCCGTAACAA
ATGCCAAAAGAAGCTAGTATAGACGAGAGCATTCAGCCAACAAATTTGCACAAGCAAAAATTGTTACCAA
TACAATTTAATATTTGTATGGGGAATTGAAACTATCTATAAAACTTTCTTCATTGCTTGATTTTTAGTAA
CGTTCAGTAATCAAACGAGACTCTTCTACCAAAAATTCGTTGTGATCTGACAAGCACTTAAATGATGGAT
ACAGATTTTATACAAATGAAAGGTAGGATTAAACTGTAAATGAATTTTAAAGGCAGAAGACTCACGTACA
CTGCACCCAGCGATTCAATTGATGTCCAGGCCTCTTTTTTCCTACTGTATACAGAAGTATGAAGTCTGCA
GCCATAATAAAAGCGGTAGGAATGATCTATGTTTTCCGCAACGCGTCAACGATTCCATGAAGAGCCTCTC
TTATGAATATTCCTAATGATCAAAACTCTAAAAAAAGCTAAAAAGAATAATTTCGCAATGCTGAAAAGTT
GAAGAGTACGGTTACACGAAGCAGTACAGGAATTCGAAATCCATCGAAAACAACTATCCAGACAAACTGA
AAAGCTAAACAGACTACTCGTTCTTTCTAGTAGACAGTATACAGGTTTTGGAATAGCAAACTATACTTAA
GGCCTATGAATCATTTCCTTCAAACTTTCAAATCAATCACCAAGCCCTCAAGAACCGTTTGATTAATTTT
CCTTGTCAACCTTCTAAGCATGGGCGTTGCGTTGAGTATAAACAAAACAGCAATAAATCCGAGTATACAT
GAATTGTTTCCTTCTTCAAACAAAACGCTGTTACCTTTGCGCGTATGATGACTTCCCATACTTACAAAAA
TTTTCAAAACCTAACA

>11
ACAGATACAATTAAGAATAACTTGTAATGGTATTCACATTACTAATAAACTTCTTAATTCATCTGTTTGG
GTATTCATTACATATTCATCAACTAAGCAAGTCTTACATGCGTCCAAATTTGTTTAAGACAAGAAAATAT
ACCATCTATGCTTAATAAACAAACTTCATCTCTTCTAGTACGCAAATAAATACATTAAATTTATTTTACA
TTATAATAAGATTCATCACTTTCTCAAAATTTTGAAACCGGAAACGGAATTCAGCATGTTTTAATAAAAA
GATGAGCAACCGCGTTCTTACTAAATACGCAATTCTAATAATTTGGGAATTGTAGTTAGTAATTTTAAAT

TTAATCATTAAAACATACCAAAAAATGGGCCCGTTCCTTTTAACATTTTGCTCAGACCAAGTGAAAAAAA
CCTAAAACGCGCTCAAGAGGAACAACGGACGAGCTACACTCAAAATATTAATGAATGGGTAGTCAACTAT
TTTACTAGCGAACAAGCAAAAGCTTTGATCCATGGATCTCACGTAATGTAAAATAGTATCTTCCCTAAGC
ATTATTTATTTGAGAAACAGGACGTTCATTATTGTTTGCCGCAAAGAATCCAAGTTCAAGGAAATTGAAT
ACAGACAAGAGTTGCTATTCGTTTGAATGTACATTGTGACCACAAGTCGGTTTTTGTTTCCTTCCAAATA
GTACCTTCAACACGAAGTTGTACATTTAGCACGGACAAAGAATCGACCAAAAAACCAAAATTAAATGTAG
AAGGAAAACCTTCAAGTCATTTTTTTTATAATAGCTCTTTTAAGTTCCATACTTTTTATCTCCGTAACAA
ATGCCAAAAGAAGCTAGTATAGACGAGAGCATTCAGCCAACAAATTTGCACAAGCAAAAATTGTTACCAA
TACAATTTAATATTTGTATGGGGAATTGAAACTATCTATAAAACTTTCTTCATTGCTTGATTTTTAGTAA
CCTTCAGTAATCAAACGAGACTCTTCTACCAAAAATTCGTTGTGATCTGACAAGCACTTAAATGATGGAT
ACAGAATTTATACAAATGAAAGGTAGGATTAAACTGTAAATGAACTTTAAAGGCAGAAGACTCACGTACA
CTACACCCAGCGATTCAATTGATGTCCAGGCCTCTTTTTTCCTACTGTATACAGAAGTATGAAGTCTGCA
GCCATAATAAAAGCGGTAGGAATGATCTATGTTTTCCGCAACGCGTCAACGATTCCATGAAGAGCCTCTC
TTATGAATGTTCCTAATGATCAAAACTCTAAAAAAAGCTAAAAAGAATAATTTCGCAATGCTGAAAAGTT
GAAGAGTACGGTTACACGAAGCAGTACAGGAATTCGAAATCCATCGAAAACAACTATCCAGACAAACTGA
AAAGCTAAACAGACTACTCGTTCTTTCTAGTAGACAGTATACAGGTTTTGGAATAGCAAACTATACTTAA
GGCCTATGAATCATTTCCTTCAAACTTTCAAATCAATCACCAAGTCCTCAAGAACCGTTTGATTAATTTT
CCTTGTCAACCTTCTAAGCATGGGCGTTGCGTTGAGTATAAACAAAACAGCAATAAATCCGAGTATACAT
GAATTGTTTCCTTCTTCAAACAAAACGCTGTTACCTTTGCGCGTATGATGACTTCCCATACTTACAAAAA
TTTTCAAAACCTAATA

>12
ACAGATACAATTAAGAATAACTTGTAATGGTATTCACATTACTAATAAACTTCTAAATTCATCTGTTTGG
GTATTCATTACATATTCATCAACTAAGCAAGTCTTACATGCGTCCAAATTTGTTTAAGACAAGAAAATAT
ACCATCTATGCTTAATAAACAAACTTCATCTCTTCTAGTACGCAAATAAATACATTAAATTTATTTTACA
TTATAATAAGATTCATCACTTTCTCAAAATTTTGAAACCGGAAACGGAATTCAGCATGTTTTAATAAAAA
GATGAGCAACCGCGTTCTTACTAAATACGCAATTCTAATAATTTGGGAATTGTAGTTAGTAATTTTAAAT
TTAATCATTAAAACATACCAAAAAATGGGCCCGTTCCTTTTAACATTTTGCTCAGACCAAGTGAAAAAAA
CCTAAAACGCGCTCAAGAGGAACAACGGACGAGCTACACTCAAAATATTAATGAATGGGTAGTCAACTAT
TTTACTAGCGAACAAGCAAAAGCTTTGATCCATGGATCTCACGTAATGTAAAATAGTATCTTCCCTAAGC
ATTATTTATTTGAGAAACAGGACGTTCATTATTGTTTGCCGCAAAGAATCCAAGTTCAAGGAAATTGAAT
ACAGACAAGAGTTGCTATTCGTTTGAATGTACATTGTGACCACAAGTCGGTTTTTGTTTCCTTCCAAATA
GTACCTTCAACACGAAGTTGTACATTTAGCACGGACAAAGAATCGACCAAAAAACCAAAATTAAATGTAG
AAGGAAAACCTTCTAGTCATTTTTTTTATAATAGCTCTTTTAAGTTCCATACTTTTTATCTCCGTAACAA
ATGCCAAAAGAAGCTAGTATAGACGAGAGCATTCAGCCAACAAATTTGCACAAGCAAAAATTGTTACCAA
TACAATTTAATATTTGTATGGGGAATTGAAACTATCTATAAAACTTTCTTCATTGCTTGATTTTTAGTAA
CCTTCAGTAATCAAACGAGACTCTTCTACCAAAAATTCGTTGTGATCTGACAAGCACTTAAATGATGGAT
ACAGAATTTATACAAATGAAAGGTAGGATTAAACTGTAAATGAACTTTAAAGGCAGAAGACTCACGTACA
CTACACCCAGCGATTCAATTGATGTCCAGGCCTCTTTTTTCCTACTGTATACAGAAGTATGAAGTCTGCA
GCCATAATAAAAGCGGTAGGAATGATCTATGTTTTCCGCAACGCGTCAACGATTCCATGAAGAGCCTCTC
TTATGAATGTTCCTAATGATCAAAACTCTAAAAAAAGCTAAAAAGAATAATTTCGCAATGCTGAAAAGTT
GAAGAGTACGGTTACACGAAGCAGTACAGGAATTCGAAATCCATCGAAAACAACTATCCAGACAAACTGA
AAAGCTAAACAGACTACTCGTTCTTTCTAGTAGACAGTATACAGGTTTTGGAATAGCAAACTATACTTAA
GGCCTATGAATCATTTCCTTCAAACTTTCAAATCAATCACCAAGTCCTCAAGAACCGTTTGATTAATTTT
CCTTGTCAACCTTCTAAGCATGGGCGTTGCGTTGAGTATAAACAAAACAGCAATAAATCCGAGTATACAT
GAATTGTTTCCTTCTTCAAACAAAACGCTGTTACCTTTGCGCGTATGATGACTTCCCATACATACAAAAA
TTTTCAAAACCTAATA

>13
ACAGATACAATTAAGAATAACTTGTAATGGTATTCACATTACTAATAAACTTCTTAATTCATCTGTTTGG
GTATTCATTACATATTCATCAACTAAGCAAGTCTTACATGCGTCCAAATTTGTTTAAGACAAGAAAATAT
ACCATCTATGCTTAATAAACAAACTTCATCTCTTCTAGTACGCAAATAAATACATTAAATTTATTTTACA
TTATAATAAGATTCATCACTTTCTCAAAATTTTGAAACCGGAAACGGAATTCAGCATGTTTTAATAAAAA
GATGAGCAACCGCGTTCTTACTAAATACGCAATTCTAATAATTTGCGAATTGTAGTTAGTAATTTTAAAT
TTAATCATTAAAACATACCAAAAAATGGGCCCGTTCCTTTTAACATTTTGCTCAGACCAAGTGAAAAAAA
CCTAAAACGCGCTCAAGAGGAACAACGGACGAGCTACACTCAAAATATTAATGAATGGGTAGTCAACTAT
TTTACTAGCGAACAAGCAAAAGCTTTGATCCATGGATCTCACGTAATGTAAAATAGTATCTTTCCTAAGC
ATTATTTATTTGAGAAACAGGACGTTCATTATTGTTTGCCGCAAAGAATCCAAGTTCAAGGAAATTGAAT

ACAGACAAGAGTTGCTATTCGTTTGAATGTACATTGTGACCACAAGTCGGTTTTTGTTTCCTTCCAAATA
GTACCTTCAACACGAAGTTGTACATTTAGCACGGACAAAGAATCGACCAAAAAACCAAAATTAAATGTAG
AAGGAAAACCTTCAAGTCATTTTTTTTATAATAGCTCTTTTAAGTTCCATACTTTTTATCTCCGTAACAA
ATGCCAAAAGAAGCTAGTATAGACGAGAGCATTCAGCCAACAAATTTGCACAAGCAAAAATTGTTACCAA
TACAATTTAATATTTGTATGGGGAATTGAAACTATCTATAAAACTTTCTTCATTGCTTGATTTTTAGTAA
CCTTCAGTAATCAAACGAGACTCTTCTACCAAAAATTCGTTGTGATCTGACAAGCACTTAAATGATGGAT
ACAGAATTTATACAAATGAAAGGTAGGATTAAACTGTAAATGAACTTTAAAGGCAGAAGACTCACGTACA
CTACACCCAGCGATTCAATTGATGTCCAGGCCTCTTTTTTCCTACTGTATACAGAAGTATGAAGTCTGCA
GCCATAATAAAAGCGGTAGGAATGATCTATGTTTTCCGCAACGCGTCAACGATTCCATGAAGAGCCTCTC
TTATGAATGTTCCTAATGATCAAAACTCTAAAAAAAGCTAAAAAGAATAATTTCGCAATGCTGAAAAGTT
GAAGAGTACGGTTACACGAAGCAGTACAGGAATTCGAAATCCATCGAAAACAACTATCCAGACAAACTGA
AAAGCTAAACAGACTACTCGTTCTTTCTAGTAGACAGTATACAGGTTTTGGAATAGCAAACTATACTTAA
GGCCTATGAATCATTTCCTTCAAACTTTCAAATCAATCACCAAGTCCTCAAGAACCGTTTGATTAATTTT
CCTTGTCAACCTTCTAAGCATGGGCGTTGCGTTGAGTATAAACAAAACAGCAATAAATCCGAGTATACAT
GAATTGTTTCCTTCTTCAAACAAAACGCTGTTACCTTTGCGCGTATGATGACTTCCCATACTTACAAAAA
TTTTCAAAACCTAATA

>14
ACAGATACAATTAAGAATAACTTGTAATGGTATTCACATTACTAATAAACTTCTAAATTCATCTGTTTGG
GTATTCATTACATATTCATCAACTAAGCAAGTCTTACATGCGTCCAAATTTGTTTAAGACAAGAAAATAT
ACCATCTATGCTTAATAAACAAACTTCATCTCTTCTAGTACGCAAATAAATACATTAAATTTATTTTACA
TTATAATAAGATTCATCACTTTCTCAAAATTTTGAAACCGGAAACGGAATTCAGCATGTTTTAATAAAAA
GATGAGCAACCGCGTTCTTACTAAATACGCAATTCTGATAATTTGGGAATTGTAGTTAGTAATTTTAAAT
TTAATCATTAAAACATACCAAAAAATGGGCCCGTTCCTTTTAACATTTTGCTCAGACCAAGTGAAAAAAA
CCTAAAACGCGCTCAAGAGGAACAACGGACGAGCTACACTCAAAATATTAATGAATGGGTAGTCAACTAT
TTTACTAGCGAACAAGCAAAAGCTTTGATCCATGGATCTCACGTAATGTAAAATAGTATCTTCCCTAAGC
ATTATTTTTTTGAGAAACAGGACGTTCATTATTGTTTGCCGCAAAGAATCCAAGTTCAAGGAAATTGAAT
ACAGACCAGAGTTGCTATTCGTTTGAATGTACATTGTGACCACAAGTCGGTTTTTGTTTCCTTCCAAATA
GTACCTTCAACACGAAGTTGTACATTTAGCACGGACAAAGAATCGACCAAAAAAACAAAATTAAATGTAG
AAGGAAAACCTTCAAGTCATTTTTTTTATAATAGCTCTTTTAAGTTCCATACTTTTTATCTCCGTAACAA
ATGCCAAAAGAAGCTAGTATAGACGAGAGCATTCAGCCAACAAATTTGCACAAGCAAAAATTGTTACCAA
TACAATTTAATATTTGTATGGGGAATTGAAACTATCTATAAAACTTTCTTCATTGCTTGATTTTTAGTAA
CGTTCAGTAATCAAACGAGACTCTTCTACCAAAAATTCGTTGTGATCTGACAAGCACTTAAATGATGGAT
ACAGATTTTATACAAATGAAAGGTAGGATTAAACTGTAAATGAATTTTAAAGGCAGAAGACTCACGTACA
CTGCACCCAGCGATTCAATTGATGTCCAGGCCTCTTTTTTCCTACTGTATACAGAAGTATGAAGTCTGCA
GCCATAATAAAAGCGGTAGGAATGATCTATGTTTTCCGCAACGCGTCAACGATTCCATGAAGAGCCTCTC
TTATGAATATTCCTAATGATCAAAACTCTAAAAAAAGCTAAAAAGAATAATTTCGCAATGCTGAAAAGTT
GAAGAGTACGGTTACACGAAGCAGTACAGGAATTCGAAATCCATCGAAAACAACTATCCAGACAAACTGA
AAAGCTAAACAGACTACTCGTTCTTTCTAGTAGACAGTATACAGGTTTTGGAATAGCAAACTATACTTAA
GGCCTATGAATCATTTCCTTCAAACTTTCAAATCAATCACCAAGCCCTCAAGAACCGTTTGATTAATTTT
CCTTGTCAACCTTCTAAGCATGGGCGTTGCGTTGAGTATAAACAAAACAGCAATAAATCCGAGTATACAT
GAATTGTTTCCTTCTTCAAACAAAACGCTGTTACCTTTGCGCGTATGATGACTTCCCATACTTACAAAAA
TTTTCAAAACCTAACA

TER1 + 30

>1
TTATTGAGAAAGTGGATTCAATCAGCTATATGTTAAGCATTAGTAATTGAAGAGGTCTCTCATTTTACTTT
AATCGAAGCTGCTATGGAATTTCGACTTTATGGCTGATTAGAAGCGTGTACTGTATACTCACCTCGTTTAA
CATTTCTTTGACTCGAATTCGCAATTGGTCTTCACGACCCATTCCAGCTTCCCGTGAAGCTTGTTCTGGTA
AATGAATAGCAAGTTTTTCTTGTATGGCTTGACGAAACACCGATTTTACGTAACTGTAATCGTTGATACTT
ATAAAGTGTTAAAATCAGATTTTCTAATTTCAAATTAAAAGCATAAATAAAAGCTCTAAACTACATGGGAT
TATGAAACACACCTTTCCAATGCAATTTTTGGTAAAACGCTAGCATCTCCGTGAGACGACATGAATGCTAG
AAAGAAATGTTTTTCTTTAAGAATATATGATTAATAGATTAGAATATATTAAGAGAAAGTTAAATTTTTAG
CTGAGAGATAGAAATCATTAGTCTGAAAAGAATTTGGATGTGATAGTA
>2
TTATTGAGAAAGTGGATTCAATCAGCTATATGTTAAGCATTAGTAATTGAAGAGGTCTCT
CATTTTACTTTAATCGAAGCTGCTATGGAATTTCGACTTTATGGCTGATTAGAAGCGTGT

ACTGTATACTCACCTCGTTTAACATTTCTTTGACTCGAATTCGCAATTGGTCTTCACGAC
CCATTCCAGCTTCCCGTGAAGCTTGTTCTGGTAAATGAATAGCAAGTTTTTCTTGTATGG
CTTGGCGAAACACCGATTTTACGTAACTGTAATCGTTGATACTTAGAAAGTGTTAAAATC
AGATTTTCTAATTTCAAATTAAAAGCATAAATAAAAGCTCTAAACTACAATGGGATTATG
AAACACACCTTTCCAATGCAATTTTTGGTAAAACGCTAGCATCTCCGTGAGACGACATGA
ATGCTAGAAAGAAATGATTTTCTTTAAGAATATATGATTAATAGATTAGAATATATTAAG
AGAAAGTTAAATTTTTAGCTGAGAGATAGAAATCATTAGTCTGAAAAGAATTTGGATGTG
ATAGTA
>3
TTATTGAGAAAGTGGATTCAATCAGCTATATGTTAAGCATTAGTAATTGGAGAGGTCTCT
CATTTTACTTTAATCGAAGCTGCTATGGAATTTCGACTTTATGGCTGATTAGAAGCGTGT
ACTGTATACTCACCTCGTTTAACATTTCTTTGACTCGAATTCGCAATTGGTCTTCACGAC
CCATTCCAGCTTCCCGTGAAGCTTGTTCTGGTAAATGAATAGCAAGTTTTTCTTGTATGG
CTTGACGAAACACCGATTTTACGTAACTGTAATCGTTGATACTTATAAAGTGTTAAAATC
AGATTTTCTAATTTCAAATTAAAAGCATAAATAAAAGCTCTAAACTACA-TGGGATTATG
AAACACACCTTTCCAATGCAATTTTTGGTAAAACGCTAGCATCTCCGTGAGACGACATGA
ATGCTAGAAAGAAATGTTTTTCTTTAAGAATATATGATTAATAGATTAGAATATATTAAG
AGAAAGTTAAATTTTTAGCTGAGAGATAGAAATCATTAGTCTGAAAAGAATTTGGATGTG
ATAGTA
>4
TTATTGAGAAAGTGGATTCAATCAGCTATATGTTAAGCATTAGTAATTGAAGAGGTCTCT
CATTTTACTTTAATCGAAGCTGCTATGGAATTTCGACTTTATGGCTGATTAGAAGCGTGT
ACTGTATACTCACCTCGTTTAACATTTCTTTGACTCGAATTCGCAATTGGTCTTCACGAC
CCATTCCAGCTTCCCGTGAAGCTTGTTCTGGTAAATGAATAGCAAGTTTTTCTTGTATGG
CTTGACGAAACACCGATTTTACGTAACTGTAATCGTTGATACTTATAAAGTGTTAAAATC
AGATTTTCTAATTTCAAATTAAAAGCATAAATAAAAGCTCTAAACTACAATGGGATTATG
AAACACACCTTTCCAATGCAATTTTTGGTAAAACGCTAGCATCTCCGTGAGACGACATGA
ATGCTAGAAAGAAATGATTTTCTTTAAGAATATATGATTAATAGATTAGAATATATTAAG
AGAAAGTTAAATTTTTAGCTGAGAGATAGAAATCATTAGTCTGAAAAGAATTTGGATGTG
ATAGTA
>5
TTATTGAGAAAGTGGATTCAATCAGCTATATGTTAAGCATTAGTAATTGAAGAGGTCTCT
CGTTTTACTTTAATCGAAGCTGCTATGGAATTTCGACTTTATGGCTGATTAGAAGCGTGT
ACTGTATACTCACCTCGTTTAACATTTCTTTGACTCGAATTCGCAATTGGTCTTCACGAC
CCATTCCAGCTTCCCGTGAAGCTTGTTCTGGTAAATGAATAGCAAGTTTTTCTTGTATGG
CATGGCGAAACACCGATTTTACGTAACTGTAATCGTTGATACTTAGAAAGTGTTAAAATC
AGATTTTCTAATTTCAAATTAAAAGCATAAATAAAAGCTCTAAACTACAATGGGATTATG
AAACACACCTTTCCAATGCAATTTTTGGTAAAACGCTAGCATCTCCGTGAGACGACATGA
ATGCTAGAAAGAAATGATTTTCTTTAAGAATATATGATTAATAGATTAGAATATATTAAG
AGAAAGTTAAATTTTTAGCTGAGAGATAGAAATCATTAGTCTGAAAAGAATTTGGATGTG
ATAGTA
>6
TTATTGAGAAAGTGGATTCAATCAGCTATATGTTAAGCATTAGTAATTGAAGAGGTCTCT
CATTTTACTTTAATCGAAGCTGCTATGGAATTTCGACTTTATGGCTGATTAGAAGCGTGT
ACTGTATACTCACCTCGTTTAACATTTCTTTGACTCGAATTCGCAATTGGTCTTCACGAC
CCATTCCAGCTTCCCGTGAAGCTTGTTCTGGTAAATGAATAGCAAGTTTTTCTTGTATGG
CTTGACGAAACACCGATTTTACGTAACTGTAATCGTTGATACTTATAAAGTGTTAAAATC
AGATTTTCTAATTTCAAATTAAAAGCATAAATAAAAGCTCTAAACTACAATGGGATTATG
AAACACACCTTTCCAATGCAATTTTTGGTAAAACGCTAGCATCTCCGTGAGACGACATGA
ATGCTAGAAAGAAATGATTTTCTTTAAGAATATATGATTAATAGATTAGAATATATTAAG
AGAAAATTAAATTTTTAGCTGAGAGATAGAAATCATTAGTCTGAAAAGAATTTGGATGTG
ATAGTA

TER1 + 100

>1
ATCAAGAAACGTTCCCGCTACTGTGCAAGTTGCGCTTGATGAATACGCTCAAAATCCCTCTACTGCATCT
GAGACACTAGTAAACAAGGAGCTTGCTAATTTTTCATCTATAAAGGAAGCTGTTTCGAAAACTCTAGAAC

TGCGGGAAAAAGTGCGGGCCCTTGAATGCGATGTCGAAATCCAAAAGCAAACTGTCCAATATCAAATTTC
CAATGCAGTTAAAGAAAATTCAAATACACTTTCAGAGCAAATGAAAAATTTGGAAAGTGAGTTAAATTCG
TCAAAAATAAAAAATGAGTCCTTACTTAATGAACGAAATTTACTGAAAGAAATGTTAGCTACTTCTCGAA
GCAGTATTTTGTCTCACAATTCTTCCGCGGGCAATATTGATGACAAAATGAAATCCATTGATGAATCTAC
AAGAGAACTTGAGAAAAATTACGAAGTTTATCGTAACGAAATGACAGCTATTCAGGAATCCTTGTCTAAG
CGAAATCAGGATTTGTTGAGTGAGATGGAAGCGATACGTAAAGAGTTAGAAAATTCCAAATATCAGCAGC
AGTTATCTACTGATAGATTAACTAATGCTAATAATGACGTTGAAGCCTT

>2
ATCAAGAAACGTTCCCGCTACTGTGCAAGTTGCGCTTGATGAATACGCTCAAAATCCCTCTACTGCATCT
GAGACACTAGTAAACAAGGAGCTTGCTAATTTTTCATCTATAAAGGAAGCTGTTTCGAAAACTCTAGAAC
TGCGGGAAAAAGTGCGGGCCCTTGAATGCGATGTTGAAATCCAAAAGCAAACTGTCCAATATCAAATTTC
CAATGCAGTTAAAGAAAATTCAAATACACTTTCAGAGCAAATCAAAAATTTGGAAAGTGAGTTAAATTCG
TCAAAAATAAAAAATGAGTCCTTACTTAATGAACGAAATTTACTGAAAGAAATGTTAGCTACTTCTCGAA
GCAGTATTTTGTCTCACAATTCTTCCGCGGGCAATATTGATGACAAAATGAAATCCATTGATGAATCTAC
AAGAGAACTTGAGAAAAATTACGAAGTTTATCGTAACGAAATGACAGCTATTCAGGAATCCTTGTCTAAG
CGAAATCAGGATTTGTTGAGTGAGATGGAAGCAATACGTAAAGAGTTAGAAAATTCCAAATATCAGCAGC
AGTTATCTACTGATAGATTAACTAATGCTAATAATGACGTTGAAGCCTT

>3
ATCAAGAAACGTTCCCGCTACTGTGCAAGTTGCGCTTGATGAATACGCTCAAAATCCCTCTACTGCATCT
GAGACACTAGTAAACAAGGAGCTTGCTAATTTTTCATCTATAAAGGAAGCTGTTTCGAAAACTCTAGAAC
TGCGGGAAAAAGTGCGGGCCCTTGAATGCGATGTCGAAATCCAAAAGCAAACTGTCCAATATCAAATTTC
CAATGCAGTTAAAGAAAATTCAAATACACTTTCAGAGCAAATCAAAAATTTGGAAAGTGAGTTAAATTCG
TCAAAAATAAAAAATGAGTCCTTACTTAATGAACGAAATTTACTGAAAGAAATGTTAGCTACTTCTCGAA
GCAGTATTTTGTCTCACAATTCTTCCGCGGGCAATATTGATGACAAAATGAAATCCATTGATGAATCTAC
AAGAGAACTTGAGAAAAATTACGAAGTTTATCGTAACGAAATGACAGCTATTCAGGAGTCCTTGTCTAAG
CGAAATCAGGATTTGTTGAGTGAGATGGAAGCGATACGTAAAGAGTTAGAAAATTCCAAATATCAGCAGC
AGTTATCTACTGATAGATTAACTAATGCTAATAATGACGTTGAAGCCTT

>4
ATCAAGAAACGTTCCCGCTACTGTGCAAGTTGCGCTTGATGAATACGCTCAAAATCCCTCTACTGCATCT
GAGACACTAGTAAACAAGGAGCTTGCTAATTTTTCATCTATAAAGGAAGCTGTTTCGAAAACTCTAGAAC
TGCGGGAAAAAGTGCGGGCCCTTGAATGCGATGTTGAAATCCAAAAGCAAACTGTCCAATATCAAATTTC
CAATGCAGTTAAAGAAAATTCAAATACACTTTCAGAGCAAATCAAAAATTTGGAAAGTGAGTTAAATTCG
TCAAAAATAAAAAATGAGTCCTTACTTAATGAACGAAATTTACTGAAAGAAATGTTAGCTACTTCTCGAA
GCAGTATTTTGTCTCACAATTCTTCCGCGGGCAATATTGATGACAAAATGAAATCCATTGATGAATCTAC
AAGAGAACTTGAGAAAAATTACGAAGTTTATCGTAACGAAATGACAGCTATTCAGGAATCCTTGTCTAAG
CGAAATCAGGATTTGTTGAGTGAGATGGAAGCGATACGTAAAGAGTTAGAAAATTCCAAATATCAGCAGC
AGTTATCTACTGATAGATTAACTAATGCTAATAATGACGTTGAAGCCTT

>5
ATCAAGAAACGTTCCCGCTACTGTGCAAGTTGCGCTTGATGAATACGCTCAAAATCCCTCTACTGCATCT
GAGACACTAGTAAACAAGGAGCTTGCTAATTTTTCATCTATAAAGGAAGCTGTTTCGAAAACTCTAGAAC
TGCGGGAAAAAGTGCGGGCCCTTGAATGCGATGTCGAAATCCAAAAGCAAACTGTCCAATATCAAATTTC
CAATGCAGTTAAAGAAAATTCAAATACACTTTCAGAGCAAATGAAAAATTTGGAAAGTGAGTTAAATTCG
TCAAAAATAAAAAATGAGTCCTTACTTAATGAACGAAATTTACTGAAAGAAATGTTAGCTACTTCTCGAA
GCAGTATTTTGTCTCACAATTCTTCCGCGGGCAATATTGATGACAAAATGAAATCCATTGATGAATCTAC
AAGAGAACTTGAGAAAAATTACGAAGTTTATCGTAACGAAATGACAGCTATTCAGGAATCCTTGTCTAAG
CGAAATCAGGATTTGTTGAGTGAGATGGAAGCGATACGTAAAGAGTTAGAAAATTCCAAATATCAGCAGC
AGTTATCTACTGATAGATTAGCTAATGCTAATAATGACGTTGAAGCCTT

CC2L

>1
CAGCACGGACGGTAAGCTCGCGATGGGCGCGATGCATGGGATTACGGGTTTGGAAAGCAACGACGCGATT
CCAGTTGTTACGCTGGAAGTCAGAGCGAAGTTGAGCAGGTGAGTAGCGGTACTCTACGAAATCGAAATGG
CGAATAGGAGTAACGGCTTGAAGTTTACCACCAACATACACATTTCCAGCACGACCAAACAAGTAGTCGA

CGGCAGGGTGGGCGCGATCATTAGCACCAAATACCTTTTCAGCTTCGTTCGCCTTATCTGGGGTATACTT
GTCCTCAACAGTCAAAATGGCAATAACAGTTTGCCCATCCCTAGGATCTAATAATGCAACACGATCACCG
GCCTTCAAAGAATCGGCCTGCGATTCATTGAGGTCCAAGGTGATAGGGATGGGAAAGACCTCGCCAGTGC
TGAGACGCAAATTCTCGACGACATTCAAATAATCCTTTTGGTTCATGAATCCGTCTAATGGACTGAAACC
TCCGTTCAAAATGAGTTCCACATCGCAAAACTGACGTTCCGATAGTACAATTTTGGGAAGGCTAGTAGCC
TCCTGTTCCAGTTGTTCTCTCAGAGGAGCATCTCTGGCGTTAAGATCTTTCAACAAAGCTTTAGTCATTT
TGACACCCCAAATCGTAAAGGACCTGCTT

>2
CAGCACGGACGGTAAGCTCACGATGGGCGCGATGCATGGGATTACGGGTTTGGAAAGCAACGACGCGATT
CCAGTTGTTACGCTGGAAGTCAGAGCGAAGTTGAGCAGGTGAGTAGCGGTACTCTACGAAATCGAAATGG
CGAATAGGAGTAACGGCTTGAAGTTTACCACCAACATACACATTTCCAGCACGACCAAACAAGTAGTCGA
CGGCAGGGTGGGCGCGATCATTAGCACCAAATACCTTTTCAGCTTCGTTCGCCTTATCTGGGGTATACTT
GTCCTCAACAGTCAAAATGGCAATAACAGTTTGCCCATCCCTAGGATCTAATAATGCAACACGATCACCG
GCCTTCAAAGAATCGGCCTGCGATTCATTGAGGTCCAAGGTGATAGGGATGGGAAAGACCTCGCCAGTGC
TGAGACGCAAATTCTCGACGACATTCAAATAATCCTTTTGGTTCATGAATCCGTCTAATGGACTGAAACC
TCCGTTCAAAATGAGTTCCACATCGCAAAACTGACGTTCCGATAGTACAATTTTGGGAAGGCTAGTAGCC
TCCTGTTCCAGTTGTTCTCTCAGAGGAGCATCTCTGGCGTTAAGATCTTTCAACAAAGCTTTAGTCATTT
TGACACCCCAAATCGTAAAGGACCTGCTT

>3
CAGCACGGACGGTAAGCTCACGATGGGCGCGATGCATGGGATTACGGGTTTGGAAAGCAACGACGCGATT
CCAGTTGTTACGCTGGAAGTCAGAGCGAAGTTGAGCAGGTGAGTAGCGGTACTCTACGAAATCGAAATGG
CGAATAGGAGTAACGGCTTGAAGTTTACCACCAACATACACATTTCCAGCACGATCAAACAAGTAGTCGA
CGGCAGGGTGGGCGCGATCATTAGCACCAAATACCTTTTCAGCTTCGTTCGCCTTATCTGGGGTATACTT
GTCCTCAACAGTCAAAATGGCAATAACAGTTTGCCCATCCCTAGGATCTAATAATGCAACACGATCACCG
GCCTTCAAAGAATCGGCCTGCGATTCATTGAGGTCCAAGGTGATAGGGATGGGAAAGACCTCGCCAGTGC
TGAGACGCAAATTCTCGACGACATTCAAATAATCCTTTTGGTTCATGAATCCGTCTAATGGACTGAAACC
TCCGTTCAAAATGAGTTCCACATCGCAAAACTGACGTTCCGATAGTACAATTTTGGGAAGGCTAGTAGCC
TCCTGTTCCAGTTGTTCTCTCAGAGGAGCATCTCTGGCGTTAAGATCTTTCAACAAAGCTTTAGTCATTT
TGACACCCCAAATCGTAAAGGACCTGCTT

>4
CAGCACGGACGGTAAGCTCACGATGGGCGCGATGCATGGGATTACGGGTTTGGAAAGCAACGACGCGATT
CCAGTTGTTACGCTGGAAGTCAGAGCGAAGTTGAGCAGGTGAGTAGCGGTACTCTACGAAATCGAAATGG
CGAATAGGAGTAACGGCTTGAAGTTTACCACCAACATACACATTTCCAGCACGACCAAACAAGTAGTCGA
CGGCAGGGTGGGCGCGATCATTAGCACCAAATACCTTTTCAGCTTCGTTCGCCTTATCTGGGGTATACTT
GTCCTCAACAGTCAAAATGGCAATAACAGTTTGCCCATCCCTAGGATCTAATAATGCAACACGATCACCG
GCCTTCAAAGAATCGGCCTGCGATTCATTGAGGTCCAAGGTGATAGGGATGGGAAAGACCTCGCCAGTGC
TGAGACGCAAATTCTCGACGACATTCAAATAATCCTTTTGGTTCATGAATCCGTCTAATGGACTGAAACC
TCCGTTCAAAATGAGTTCCACATCGCAAAACTGACGTTCCGATAGTACAATTTTGGGAAGGCTAGTAGCC
TCCTGTTCCAGTTGTTCTCTCAGAGGAGCATCTCTGGCGTTAAGATCTTTCAACAAAGCTTTAGTCATTT
TGACACCCCAAATCGTAAAGGACCTGGTT

>5
CAGCACGGACGGTAAGCTCACGATGGGCGCGATGCATGGGATTACGGGTTTGGAAAGCAACGACGCGATT
CCAGTTGTTACGCTGGAAGTCAGAGCGAAGTTGAGCAGGTGAGTAGCGGTACTCTACGAAATCGAAATGG
CGAATAGGAGTAACGGCTTGAAGTTTACCACCAACATACACATTTCCAGCACGACCAAACAAGTAGTCGA
CGGCAGGGTGGGCGCGATCATTAGCACCAAATACCTTTTCAGCTTCGTTCGCCTTATCTGGGGTATACTT
GTCCTCAACAGTCAAAATGGCAATAACAGTTTGCCCATCCCTAGGATCTAATAATGCAACACGATCACCG
GCCTTCAAAGAATCGGCCTGCGATTCATTGAGGTCCAAGGTGATAGGGATGGGAAAGACCTCGCCAGTGC
TGAGACGCAAATTCTCGACGACATTCAAGTAATCCTTTTGGTTCATGAATCCGTCTAATGGACTGAAACC
TCCGTTCAAAATGAGTTCCACATCGCAAAACTGACGTTCCGATAGTACAATTTTGGGAAGGCTAGTAGCC
TCCTGTTCCAGTTGTTCTCTCAGAGGAGCATCTCTGGCGTTAAGATCTTTCAACAAAGCTTTAGTCATTT
TGACACCCCAAATCGTAAAGGACCTGCTT

CC2R

>1
GTGCCTTTATATTCTTTAACAAGGCTATATGTTTTAGCTTTGTAAGAAGGATGGCGATCTCGTACAGCGG
TACGATGTCCCAAGACACCACCCAAGACACCAATGAGAACACTACAAATTAAAGCGACTATAAAAATTAA
TATCCAAATAAAGTAACGTCGGTACCAAGGCAATAACTTTTTCTCTGCAGCCTTTTCGTTAAAGGCTTCA
TTGTTTTCGTTATTATTATTGCTATCAAGGTTTTCATGGTCATTATCGTGATCATCAATTTCAGTGTCTG
TATGATTGTCGATAACATCATTAGTTGACTCGTTCTTGCGTGCCGGGTCGTAAATATTCTCAAAGCTAGC
AGAGGACAATGCAGCAGATGAGAGACTATGGCGTGAATCTGTAGTGGAATCAGGAATACCCATTGCAAAA
GTAGTAAAAGTTAATCAAATTTCAACAAATTAAAGAAGGGCGAGAAAAATCAAAAAGAAAGAAAAGATAA
GAATAAAAAGAGCGGGAAGTAATGAGACACGATCAAATAAAAAAATGCCAATCTGGTGAGCTATGCACCA
AGTTCAGAAATGGAAATAAAATGCTAGTAAAAATAACCAAACTCAAGGATATCTTGTTAAAGGGGAATAA
AAAAGGGAAATATGGGATAATGCTTTGTAGAGATGAGAAATGCGGTTTAAAATGAAAATAATTATTATCG
AAAGACGATAGATAGGGAG

>2
GTGCCTTTATATTCTTTAACAAGGCTATATGTTTTAGCTTTGTAAGAAGGATGGCGATCTCGTACAGCGG
TACGATGTCCCAAGACACCACCCAAGACACCAATGAGAACACTACAAATTAAAGCGACTATAAAAATTAA
TATCCAAATAAAGTAACGTCGGTACCAAGGCAATAACTTTTTCTCTGCAGCCTTTTCGTTAAAGGCTTCA
TTGTTTTCGTTATTATTATTGCTATCAAGGTTTTCATGGTCATTATCGTGATCATCAATTTCAGTGTCTG
TATGATTGTCGATAACATCATTAGTTGACTCGTTCTTGCGTGCCGGGTCGTAAATATTCTCAAAGCTAGC

AGAGGACAATGCAGCAGATGAGAGACTATGGCGTGAATCTGTCGTGGAATCAGGAATACCCATTGCAAAA
GTAGTAAAAGTTAATCAAATTTCAACAAATTAAAGAAGGGCGAGAAAAATCAAAAAGAAAGAAAAGATAA
GAATAAAAAGAGCGGGAAGTAATGAGACACGATCAAATAAAAAAATGCCAATCTGGTGAGCTATGCACCA
AGTTCAGAAATGGAAATAAAATGCTAGTAAAAATAACCAAACTCAAGGATATCTTGTTAAAGGGGAATAA
AAAAGGGAAATATGGGATAATGCTTTGTAGAGATGAGAAATGCGGTTTAAAATGAAAATAATTATTATCG
AAAGACGATAGATAGGGAG

>3
GTGCCTTTATATTCTTTAACAAGGCTATATGTTTTAGCTTTGTAAGAAGGATGGCGATCTCGTACAGCGG
TACGATGTCCCAAGACACCACCCAAGACACCAATGAGAACACTACAAATTAAAGCGACTATAAAAATTAA
TATCCAAATAAAGTAACGTCGGTACCAAGGCAATAACTTTTTCTCTGCAGCCTTTTCGTTAAAGGCTTCA
TTGTTTTCGTTATTATTATTGCTATCAAGGTTTTCATGGTCATTATCGTGATCATCAATTTCAGTGTCTG
TATGATTGTCGATAACATCATTAGTTGACTCGTTCTTGCGTGCCGGGTCGTAAATATTCTCAAAGCTAGC
AGAGGACAACGCAGCAGATGAGAGACTATGGCGTGAATCTGTAGTGGAATCAGGAATACCCATTGCAAAA
GTAGTAAAAGTTAATCAAATTTCAACAAATTAAAGAAGGGCGAGAAAAATCAAAAAGAAAGAAAAGATAA
GAATAAAAAGAGCGGGAAGTAATGAGACACGATCAAATAAAAAAATGCCAATCTGGTGAGCTATGCACCA
AGTTCAGAAATGGAAATAAAATGCTAGTAAAAATAACCAAACTCAAGGATATCTTGTTAAAGGGGAATAA
AAAAGGGAAATATGGGATAATGCTTTGTAGAGATGAGAAATGCGGTTTAAAATGAAAATAATTATTATCG
AAAGACGATAGATAGGGAG

>4
GTGCCCTTATATTCTTTAACAAGGCTATATGTTTTAGCTTTGTAAGAAGGATGGCGATCTCGTACAGCGG
TACGATGTCCCAAGACACCACCCAAGACACCAATGAGAACACTACAAATTAAAGCGACTATAAAAATTAA
TATCCAAATAAAGTAACGTCGGTACCAAGGCAATAACTTTTTCTCTGCAGCCTTTTCGTTAAAGGCTTCA
TTGTTTTCGTTATTATTATTGCTATCAAGGTTTTCATGGTCATTATCGTGATCATCAATTTCAGTGTCTG
TATGATTGTCGATAACATCATTAGTTGACTCGTTCTTGCGTGCCGGGTCGTAAATATTCTCAAAGCTAGC
AGAGGACAATGCAGCAGATGAGAGACTATGGCGTGAATCTGTAGTGGAATCAGGAATACCCATTGCAAAA
GTAGTAAAAGTTAATCAAATTTCAACAAATTAAAGAAGGGCGAGAAAAATCAAAAAGAAAGAAAAGATAA
GAATAAAAAGAGCGGGAAGTAATGAGACACGATCAAATAAAAAAATGCCAATCTGGTGAGCTATGCACCA
AGTTCAGAAATGGAAATAAAATGCTAGTAAAAATAACCAAACTCAAGGATATCTTGTTAAAGGGGAATAA
AAAAGGGAAATATGGGATAATGCTTTGTAGAGATGAGAAATGCGGTTTAAAATGAAAATAATTATTATCG
AAAGACGATAGATAGGGAG

>5
GTGCCTTTATATTCTTTAACAAGGCTATATGTTTTAGCTTTGTAAGAAGGATGGCGATCTCGTACAGCGG
TACGATGTCCCAAGACACCACCCAAGACACCAATGAGAACACTACAAATTAAAGCGACTATAAAAATTAA
TATCCAAATAAAGTAACGTCGGTACCAAGGCAATAACTTTTTCTCTGCAGCCTTTTCGTTAAAGGCTTCA
TTGTTTTCGTTATTATTATTGCTATCAAGGTTTTCATGGTCATTATCGTGATCATCAATTTCAGTGTCTG
TATGATTGTCGATAACATCATTAGTTGACTCGTTCTTGCGTGCCGGGTCGTAAATATTCTCAAAGCTAGC
AGAGGACAACGCAGCAGATGAGAGACTATGGCGTGAATCTGTAGTGGAATCAGGAATACCCATTGCAAAA
GTAGTAAAAGTTAATCAAATTTCAACAAATTAAAGAAGGGCGAGAAAAATCAAAAAGAAAGAAAAGATAA
GAATAAAAAGAGCGGGAAGTAATGAGACACGATCAAATAAAAATATGCCAATCTGGTGAGCTATGCACCA
AGTTCAGAAATGGAAATAAAATGCTAGTAAAAATAACCAAACTCAAGGATATCTTGTTAAAGGGGAATAA
AAAAGGGAAATATGGGATAATGCTTTGTAGAGATGAGAAATGCGGTTTAAAATGAAAATAATTATTATCG
AAAGACGATAGATAGGGAG

>6
GTGCCTTTATATTCTTTAACAAGGCTATATGTTTTAGCTTTGTAAGAAGGATGGCGATCTCGTACAGCGG
TACGATGTCCCAAGACACCACCCAAGACACCAATGAGAACACTACAAATTAAAGCGACTATAAAAATTAA
TATCCAAATAAAGTAACGTCGGTACCAAGGCAATAACTTTTTCTCTGCAGCCTTTTCGTTAAAGGCTTCA
TTGTTTTCGTTATTATTATTGCTATCAAGGTTTTCATGGTCATTATCGTGATCATCAATTTCAGTGTCTG
TATGATTGTCGATAACATCATTAGTTGACTCGTTCTTGCGTGCCGGGTCGTAAATATTCTCAAAGCTAGC
AGAGGACAACGCAGCAGATGAGAGACTATGGCGTGAATCTGTAGTGGAATCAGGAATACCCATTGCAAAA
GTAATAAAAGTTAATCAAATTTCAACAAATTAAAGAAGGGCGAGAAAAATCAAAAAGAAAGAAAAGATAA
GAATAAAAAGAGCGGGAAGTAATGAGACACGATCAAATAAAAAAATGCCAATCTGGTGAGCTATGCACCA
AGTTCAGAAATGGAAATAAAATGCTAGTAAAAATAACCAAACTCAAGGATATCTTGTTAAAGGGGAATAA
AAAAGGGAAATATGGGATAATGCTTTGTAGAGATGAGAAATGCGGTTTAAAATGAAAATAATTATTATCG
AAAGACGATAGATAGGGAG

>7
GTGCCCTTATATTCTTTAACAAGGCTATATGTTTTAGCTTTGTAAGAAGGATGGCGATCTCGTACAGCGG
TACGATGTCCCAAGACACCACCCAAGACACCAATGAGAACACTACAAATTAAAGCGACTATAAAAATTAA
TATCCAAATAAAGTAACGTCGGTACCAAGGCAATAACTTTTTCTCTGCAGCCTTTTCGTTAAAGGCTTCA
TTGTTTTCGTTATTATTATTGCTATCAAGGTTTTCATGGTCATTATCGTGATCATCAATTTCAGTGTCTG
TATGATTGTCGATAACATCATTAGTTGACTCGTTCTTGCGTGCCGGGTCGTAAATATTCTCAAAGCTAGC
AGAGGACAACGCAGCAGATGAGAGACTATGGCGTGAATCTGTAGTGGAATCAGGAATACCCATTGCAAAA
GTAGTAAAAGTTAATCAAATTTCAACAAATTAAAGAAGGGCGAGAAAAATCAAAAAGAAAGAAAAGATAA
GAATAAAAAGAGCGGGAAGTAATGAGACACGATCAAATAAAAAAATGCCAATCTGGTGAGCTATGCACCA
AGTTCAGAAATGGAAATAAAATGCTAGTAAAAATAACCAAACTCAAGGATATCTTGTTAAAGGGGAATAA
AAAAGGGAAATATGGGATAATGCTTTGTAGAGATGAGAAATGCGGTTTAAAATGAAAATAATTATTATCG
AAAGACGATAGATAGGGAG

Central core chromosome II

>1
AGATTTGAGCAATAATTTCCTAAAACATTTACAATTAATGGGTTATTTTTTTTTT-CTAAAAATTGAAGG
AGCTTGTTTGATCTATTCCGTTATTAGTATGTAAATTTAAAAAATCGTATCCCTAACTAATTCATACTTA
AACATAATATGCAGATTTTAAAATAATTGTCCATATAGATAACACGCGGAATACTTAGAAAAGTAGAATT
GGCCGAAGCCAAAAAGGAAACATAGAAATCAACCAGGACTAACCAATGCTTCTTCATATTAACGAGTAAC
GCGGCTTACGCATCACAAAGCAGTACGTGTGAATAAATATTATTGGAAAACATTTTTCTTTAACCAGCTA
AACCGCTTAAAGAAAAATGAATAAGATACAAATTTTCAGGTTTTTGCTTTGATGCTAAAAACTCGTCGAA
TTCTAAAACCAAGAATTAAACGTACCCTTAAACTTTTTTTTT-GATTTTCGTACTAATAATTATCGGAG
TTCGCGTTTTCATTTTAAAAAAA-GGTTTAGTAAATCTCAAATTGCGATACGAACAATGATAATGATATT
TTCAGATAAACGTTACTTAATAGCATCTTATAGTCATGGGCCATCTCAATATTACCATTAAACTTTTAAG
CAAAAAGATCATTTCTATTTTAAAAAACCTAATAAAAATTACCTTTTGTAAGTATACGTTTGCTAAACAA
TAAAAGAACGTAATACTAA

>2
AGATTTGAGCAATAATTTCCTAAAACATTTACAATTAATGGGTTATTTTTTTTTT-CTAAAAATTGAAGG
AGCTTGTTTGATCTATTCCGTTATTAGTATGTAAATTTAAAAAATCGTATCCCTAACTAATTCATACTTA
AACATAATATGCAGATTTTAAAATAATTGTCCATATAGATAACACGCGGAATACTTAGAAAAGTAGAATT
GGCCGAAGCCAAAAAGGAAACATAGAAATCAACCAGGACTAACCAATGCTTCTTCATATTAACGAGTAAC
GCGGCTTACGCATCACAAAGCAGTACGTGTGAATAAATATTATTGGAAAACATTTTTCTTTAACCAGCTA
AACCGCTTAAAGAAAAATGAATAAGATACAAATTTTCAGGTTTTTGCTTTGATGCTAAAAACTCGTCGAA
TTCTAAAACCAAGAATTAAACGTACCCTTAAACTTTTTTTTTT-GATTTTCGTACTAATAATTATCGGAG
TTCGCGTTTTCATTTTAAAAAAAAGGTTTAGTAAATCTCAAATTGCGATACGAACAATGATAATGATATT
TTCAGATAAACGTTACTTAATAGCATCTTATAGTCATGGGCCATCTCAATATTACCATTAAACTTTTAAG
CAAAAAGATCATTTCTATTTTAAAAAACCTAATAAAAATTACCTTTTGTAAGTATACGTTTGCTAAACAA
TAAAAGAACGTAATACTAA

>3
AGATTTGAGCAATAATTTCCTAAAACATTTACAATTAATGGGTTATTTTTTTTTT-CTAAAAATTGAAGG
AGCTTGTTTGATCTATTCCGTTATTAGTATGTAAATTTAAAAAATCGTATCCCTAACTAATTCATACTTA
AACATAATATGCAGATTTTAAAATAATTGTCCATATAGATAACACGCGGAATACTTAGAAAAGTAGAATT
GGCCGAAGCCAAAAAGGAAACATAGAAATCAACCAGGACTAACCAATGCTTCTTCATATTAACGAGTAAC
GCGGCTTACGCATCACAAAGCAGTACGTGTGAATAAATATTATTGGAAAACATTTTTCTTTAACCAGCTA
AACCGCTTAAAGAAAAATGAATAAGATACAAATTTTCAGGTTTTTGCTTTGATGCTAAAAACTCGTCGAA
TTCTAAAACCAAGAATTAAACGTACCCTTAAACTTTTTTTTT--GATTTTCGTACTAATAATTATCGGAG
TTCGCGTTTTCATTTTAAAAAAA-GATTTAGTAAATCTCAAATTGCGATACGAACAATGATAATGATATT
TTCAGATAAACGTTACTTAATAGCATCTTATAGTCATGGGCCATCTCAATATTACCATTAAACTTTTAAG
CAAAAAGATCATTTCTATTTTAAAAAACCTAATAAAAATTACCTTTTGTAAGTATACGTTTGCTAAACAA
TAAAAGAACGTAATACTAA

>4
AGATTTGAGCAATAATTTCCTAAAACATTTACAATTAATGGGTTATTTTTTTTTT-CTAAAAATTGAAGG
AGCTTGTTTGATCTATTCCGTTATTAGTATGTAAATTTAAAAAATCGTATCCCTAACTAATTCATACTTA
AACATAATATGCAGATTTTAAAATAATTGTCCATATAGATAACACGCGGAATACTTAGAAAAGTAGAATT
GGCCGAAGCCAAAAAGGAAACATAGAAATCAACCAGGACTAACCAATGCTTCTTCATATTAACGAGTAAC
GCGGCTTACGCATCACAAAGCAGTACGTGTGAATAAATATTATTGGAAAACATTTTTCTTTAACCAGCTA
AACCGCTTAAAGAAAAATGAATAAGATACAAATTTTCAGGTTTTTGCTTTGATGCTAAAAACTCGTCGAA
TTCTAAAACCAAGAATTAAACGTACCCTTAAACTTTTTTTTTT-GATTTTCGTACTAATAATTATCGGAG
TTCGCGTTTTCATTTTAAAAAAA-GGTTTAGTAAATCTCAAATTGCGA-----ACAATGATAATGATATT
TTCAGATAAACGTTACTTAATAGCATCTTATAGTCATGGGCCATCTCAATATTACCATTAAACTTTTAAG
CAAAAAGATCATTTCTATTTTAAAAAACCTAATAAAAATTACCTTTTGTAAGTATACGTTTGCTAAACAA
TAAAAGAACGTAATACTAA

>5
AGATTTGAGCAATAATTTCCTAAAACATTTACAATTAATGGGTTATTTTTTTTTT-CTAAAAATTGAAGG
AGCTTGTTTGATCTATTCCGTTATTAGTATGTAAATTTAAAAAATCGTATCCCTAACTAATTCATACTTA
AACATAATATGCAGATTTTAAAATAATTGTCCATATAGATAACACGCGGAATACTTAGAAAAGTAGAATT
GGCCGAAGCCAAAAAGGAAACATAGAAATCAACCAGGACTAACCAATGCTTCTTCATATTAACGAGTAAC
GCGGCTTACGCATCACAAAGCAGTACGTGTGAATAAATATTATTGGAAAACATTTTTCTTTAACCAGCTA
AACCGCTTAAAGAAAAATGAATAAGATACAAATTTTCAGGTTTTTGCTTTGATGCTAAAAACTCGTCGAA
TTCTAAAACCAAGAATTAAACGTACCCTTAAACTTTTTTTTTT-GATTTTCGTACTAATAATTATCGGAG
TTCGCGTTTTCATTTTAAAAAAA-GGTTTAGTAAATCTCAAATTGCGATACGAACAATGATAATGATATT
TTCAGATAAACGTTACTTAATAGCATCTTATAGTCATGGGCCATCTCAATATTACCATTAAACTTTTAAG
CAAAAAGATCATTTCTATTTTAAAAAACCTAATAAAAATTACCTTTTGTAAGTATACGTTTGCCAAACAA
TAAAAGAACGTAATACTAA

>6
AGATTTGACCAATAATTTCCTAAAACATTTACAATTAATGCGTTATTTTTTTTTTTCTAAAAATTGAAGG
AGCTTGTTTGATCTATTCCGTTATTAGTATGTAAATTTAGAAAATCGTATCCCTAACTAATTCATACTTA
AACCTAATATGCAGATTTTAAAATAATAGTCCATAAAGATAACACACGGAATACTTAAAAAAGTAGAATT
GGCCGAAGCCAAAAAGGAAACATAGAAATCAACCAGGACTAACCAATGCTTCTTCATATTAACGAGTAAC
--GGCTTACGCATCACAAAGCAGTACATGTGAATAAATATTATTGGAAAACATTTTTCTTTAACCAGCTA
AACCGCTTAAAGAAAAATGAATAAGATACAAATCTTCAGGTTTTTGCTTTGATGCTAAAAACTCGTCGAA
TTTTAAAACCAAGAATTAAACGTACCCTTCAACTTTTTTTTTTTGATTTTCGTACTAATGATTATCGGAG
TTCGCGTTTTCATTTTAAAAAAA-GGTTTAGTAAATCTCAAATTGCGATACGAACAATGATAATGATATT
TTCAGATAAACGTTACTTAATAGCATCTTATAGTCATGGGCCATCTCAATATTACCATTAAACTTTTAAG
CAAAAAGATCATTTCTATTTTAAAAAATCTAATAAAAATTACCTTTTGTAAGTATACGTTTGCTAAACAA
TAAAAGAACATATTACTAA

>7
AGATTTGAGCAATAATTTCCTAAAACATTTACAATTAATGGGTTATTTTTTTTTT-CTAAAAATTGAAGG
AGCTTGTTTGATCTATTCCGTTATTAGTATGTAAATTTAAAAAATCGTATCCCTAACTAATTCATACTTA
AACATAATATGCAGATTTTAAAATAATTGTCCATATAGATAACACGCGGAATACTTAGAAAAGTAGAATT
GGCCGAAGCCAAAAAGGAAACATAGAAATCAACCAGGACTAACCAATGCTTCTTCATATTAACGAGTAAC
GCGGCTTACGCATCACAAAGCAGTACGTGTGAATAAATATTATTGGGAAACATTTTTCTTTAACCAGCTA
AACCGCTTAAAGAAAAATGAATAAGATACAAATTTTCAGGTTTTTGCTTTGATGCTAAAAACTCGTCGAA
TTCTAAAACCAAGAATTAAACGTACCCTTAAACTTTTTTTTTT-GATTTTCGTACTAATAATTATCGGAG
TTCGCGTTTTCATTTTAAAAAAA-GGTTTAGTAAATCTCAAATTGCGA-----ACAATGATAATGATATT
TTCAGATAAACGTTACTTAATAGCATCTTATAGTCATGGGCCATCTCAATATTACCATTAAACTTTTAAG
CAAAAAGATCATTTCTATTTTAAAAAACCTAATAAAAATTACCTTTTGTAAGTATACGTTTGCTAAACAA
TAAAAGAACGTAATACTAA

>8
AAATTTGAGCAATAATTTCCTAAAACATTTACAATTAATGGGTTATTTTTTTTTT-CTAAAAATTGAAGG
AGCTTGTTTGATCTATTCCGTTATTAGTATGTAAATTTAAAAAATCGTATCCCTAACTAATTCATACTTA
AACATAATATGCAGATTTTAAAATAATTGTCCATATAGATAACACGCGGAATACTTAGAAAAGTAGAATT
GGCCGAAGCCAAAAAGGAAACATAGAAATCAACCAGGACTAACCAATGCTTCTTCATATTAACGAGTAAC
GCGGCTTACGCATCACAAAGCAGTACGTGTGAATAAATATTATTGGAAAACATTTTTCTTTAACCAGCTA
AACCGCTTAAAGAAAAATGAATAAGATACAAATTTTCAGGTTTTTGCTTTGATGCTAAAAACTCGTCGAA
TTCTAAAACCAAGAATTAAACGTACCCTTAAACTTTTTTTTTT-GATTTTCGTACTAATAATTATCGGAG
TTCGCGTTTTCATTTTAAAAAAA-GGTTTAGTAAATCTCAAATTGCGATACGAACAATGATAATGATATT
TTCAGATAAACGTTACTTAATAGCATCTTATAGTCATGGGCCATCTCAATATTACCATTAAACTTTTAAG
CAAAAAGATCATTTCTATTTTAAAAAACCTAATAAAAATTACCTTTTGTAAGTATACGTTTGCTAAACAA
TAAAAGAACGTAATACTAA

>9
AGATTTGACCAATAATTTCCTAAAACATTTACAATTAATGCGTTATTTTTTTTTTTCTAAAAATTGAAGG
AGCTTGTTTGATCTATTCCGTTATTAGTATGTAAATTTAGAAAATCGTATCCCTAACTAATTCATACTTA
AACCTAATATGCAGATTTTAAAATAATAGTCCATAAAGATAACACGCGGAATACTTAAAAAAGTAGAATT
GGCCGAAGCCAAAAAGGAAACATAGAAATCAACCAGGACTAACCAATGCTTCTTCATATTAACGAGTAAC
--GGCTTACGCATCACAAAGCAGTACATGTGAATAAATATTATTGGAAAACATTTTTCTTTAACCAGCTA
AACCGCTTAAAGAAAAATGAATAAGATACAAATCTTCAGGTTTTTGCTTTGATGCTAAAAACTCGTCGAA
TTTTAAAACCAAGAATTAAACGTACCCTTCAACTTTTTTTTTTTGATTTTCGTACTAATGATTATCGGAG
TTCGCGTTTTCATTTTAAAAAAA-GGTTTAGTAAATCTCAAATTGCGATACGAACAATGATAATGATATT
TTCAGATAAACGTTACTTAATAGCATCTTATAGTCATGGGCCATCTCAATATTACCATTAAACTTTTAAG
CAAAAAGATCATTTCTATTTTAAAAAATCTAATAAAAATTACCTTTTGTAAGTATACGTTTGCTAAACAA
TAAAAGAACATATTACTAA

>10
AGATTTGAGCAATAATTTCCTAAAACATTTACAATTAATGGGTTATTTTTTTTTT-CTAAAAATTGAAGG
AGCTTGTTTGATCTATTCCGTTATTAGTATGTAAATTTAAAAAATCGTATCCCTAACTAATTCATACTTA
AACATAATATGCAGATTTTAAAATAATTGTCCATATAGATAACACGCGGAATACTTAGAAAAGTAGAATT
GGCCGAAGCCAAAAAGGAAACATAGAAATCAACCAGGACTAACCAATGCTTCTTCATATTAACGAGTAAC
GCGGCTTACGCATCACAAAGCAGTACGTGTGAATAAATATTATTGGAAAACATTTTTCTTTAACCAGCTA
AACCGCTTAAAGAAAAATGAATAAGATACAAATTTTCAGGTTTTTGCTTTGATGCTAAAAACTCGTCGAA
TTCTAAAACCAAGAATTAAACGTACCCTTAAACTTTTTTTTTT-GATTTTCGTACTAATAATTATCGGAG
TTCGCGTTTTCATTTTAAAAAAA-GGTTTAGTAAATCTCAAATTGCGATACGAACAATGATAATGATATT
TTCAGATAAACGTTACTTAATAGCATCTTATAGTCATGGGCCATCTCAATATTACCATTAAACTTTTAAG
CAAAAAGATCATTTCTATTTTAAAAAACCTAATAAAAATTACCTTTAGTAAGTATACGTTTGCTAAACAA
TAAAAGAACGTAATACTAA
