# Supplementary material for: A Geographically Diverse Collection of Schizosaccharomyces pombe Isolates Shows Limited Phenotypic Variation but Extensive Karyotypic Diversity
Source: G3 (Bethesda). 2011 Dec 1;1(7):615–26. doi: 10.1534/g3.111.001123 (PMC3276172; doi:10.1534/g3.111.001123)
Supplement: Supporting Information [file supp_1.7.615_TableS3.pdf]

Table S3 Primers used to make probes used in hybridization analysis

Name	Sequence	Chromosome	Position
CENDistal1LF	CCCAAGAAACGTCATGTGTG	1	196555-196574
CENDistal1LR	CTACCAGGAGCCTTCAGTGC	1	197476-197457
CENDistal1RF	CACTTCGTGCAAAGCAAAAA	1	4745750-4745769
CENDistal1RR	ACAATTGCCAACCTCCTCAC	1	4746658-4746639
CENDistal2LF	TTGGTCTCGAAGGAGCATCT	2	751963-751982
CENDistal2LR	ATTCCACCAATTTCCCAACA	2	752829- 752810
CENDistal2RF	TTCGATAGGTCGGATTCCAG	2	3761023-3761042
CENDistal2RR	ATCTCTTGCCAACCACCAAC	2	3761895-3761876
CENDistal3LF	TCAAACAATCCATCGGACAA	3	501513-501532
CENDistal3LR	GATGCGGAAGCTAAATCGAG	3	502474-502455
CENDistal3RF	GTGACGTAAATGCCCGAGTT	3	2004488-2004507
CENDistal3RR	AGATTCGGGTTTCAGTGGTG	3	2005446-2005465
CENproximal1LR	AAAGGCTGTCGGTGTTATGG	1	3733956-3733937
CENproximal1LF	CGGTGAACCTGACAATGATG	1	3733183-3733202
CENproximal1RF	CGTCAATCCTCAATTCAGAGTTCC	1	3792899-3792922
CENproximal1RR	TATGTCAAAGAAGCCTCGATAAAGAAG	1	3793712-3793738
CENproximal2LF	GATCGATATCACCAGGCTTGGTC	2	1572261-1572283
CENproximal2LR	GCGAGAAATTGCCAATTGCCGAC	2	1573046-1573068
CENproximal2RF	AAGTGACATCACAAGTCCGACTTC	2	1650281-1650304
CENproximal2RL	AACTTGGAAACCAATAAAATTGAG	2	1651324-1651301
CENproximal3LF	AATAGGGAAGCCGATTGTTCTTACAGATGT	3	1061733-1061762
CENproximal3LR	TATCTATGGAAAGCATTAGAACGA	3	1062572-1062549
CENproximal3RF	GTTCGTCAATTTATTAATCATTAG	3	1145013-1145036
CENproximal3RR	AACCCCTTACACCTCCTCAAGATC	3	1145732-1145709
