# Supplementary material for: A Geographically Diverse Collection of Schizosaccharomyces pombe Isolates Shows Limited Phenotypic Variation but Extensive Karyotypic Diversity
Source: G3 (Bethesda). 2011 Dec 1;1(7):615–26. doi: 10.1534/g3.111.001123 (PMC3276172; doi:10.1534/g3.111.001123)
Supplement: Supporting Information [file supp_1.7.615_TableS4.pdf]

Table S4 Primers used to amplify DNA for diversity analys

Sequence	Primer F	Sequence	Primer R	Sequence
SPBC660.16 intron 3	SPBC660L	gccgacaaggggtaagtttt	SPBC660R	ttacctttggcttcgtttgc
Central core of centromere of chromosome II	CNT21L	cacagaagcgagacatgttt	CNT21R	ggataagacacttcgccaaaa
TER	TRC19F	tgtttgatctacctcgtttatattca	TRC1409R	aaaaagaggcctggacatca
TER	TRC667F	aacctaaaacgcgctcaaga	TRC1983R	ttgaatgtaatgttagcccttaaaa
CEN2:left flank	CENFlank2LF	gatcgatatcaccaggcttggtc	CENFlank2LR	gcgagaaattgccaattgccgac
CEN2:right flank	CENFlank2iRF	gcactgctgttacggtcaag	CENFlank2iRR	aagctggttggctttttatcc
TER; I-3113975-3114540	TRCflank1F	gccagatcaaatggctcaat	TRCflank1R	acaacgttgggatcaagagg
TER; I-3194538-3195201	100TRCFlank1F	ttaaacgctcttgcgtcctt	100TRCFlank1R	ctcaccgcatctcgtactca
