# Supplementary material for: A Geographically Diverse Collection of Schizosaccharomyces pombe Isolates Shows Limited Phenotypic Variation but Extensive Karyotypic Diversity
Source: G3 (Bethesda). 2011 Dec 1;1(7):615–26. doi: 10.1534/g3.111.001123 (PMC3276172; doi:10.1534/g3.111.001123)
Supplement: Supporting Information [file supp_1.7.615_FigureS8.pdf]

Haplotype: abundance	3084500	3084506	3084568	3084571	3084743	**3084762**	3084771	3084778	3084779	3084929	3084934	3084944	3084970	3085000	3085001	3085013	3085016	3085085	3085203	3085232	3085238	3085242	3085352	3085405	3085432	3085436	3085448	3085464	3085507	3085520	3085546	3085574	3085611	3085720	3085776	3085966	**3086123**	3086146
1:1	T	G	T	A	T	A	G	A	A	G	C	G	T	A	C	T	A	A	C	A	T	T	T	T	C	T	G	A	A	A	C	A	T	G	A	T	T	T
5:1	.	.	.	.	.	.	.	.	.	.	.	.	.	.	.	.	.	.	.	.	-	.	.	.	.	.	.	.	.	.	.	.	.	.	.	.	.	.
2:1	.	A	.	.	.	.	.	.	.	.	.	A	.	.	.	A	.	.	.	.	-	.	.	.	.	.	.	.	.	.	.	.	.	.	.	.	.	.
13:2	.	A	.	.	.	.	.	.	.	.	.	A	.	.	.	.	.	.	.	.	-	.	.	.	.	.	.	.	.	T	.	.	.	.	.	.	.	.
15:1	.	A	.	.	.	.	C	.	.	.	.	A	.	-	T	.	.	.	.	.	-	.	.	.	.	.	.	.	.	T	.	.	.	.	.	.	.	.
3:5	A	A	.	.	.	.	.	.	.	.	.	A	.	.	.	.	.	.	.	.	-	.	.	.	.	.	.	.	.	T	.	.	.	.	.	.	A	.
7:3	A	A	.	.	.	.	.	.	.	C	.	A	.	.	.	.	.	.	.	.	-	.	.	.	.	.	.	G	.	T	.	.	.	.	.	.	A	.
14:1	A	A	.	.	.	.	.	-	-	.	.	A	.	.	.	.	.	.	.	T	-	.	.	.	.	.	.	.	.	T	.	.	.	.	.	.	A	.
11:1	A	A	.	.	.	G	.	.	.	.	.	A	.	.	.	.	.	.	.	.	-	.	.	.	.	.	.	.	.	T	.	.	.	.	.	.	A	.
10:1	A	A	.	G	G	.	.	.	.	.	T	A	.	.	.	.	.	.	.	.	-	.	C	.	.	.	A	.	.	T	T	.	C	.	G	C	.	.
9:1	A	A	C	.	G	.	.	.	.	.	.	A	.	.	.	.	.	.	.	.	-	-	C	.	.	.	A	.	.	T	T	.	C	.	G	C	.	.
4:2	A	A	.	.	.	G	.	.	.	.	.	A	.	.	.	.	T	C	.	.	-	-	.	A	G	.	.	.	T	T	T	G	.	A	.	C	.	C
6:17	A	A	.	.	.	G	.	.	.	.	.	A	.	.	.	.	T	C	.	.	-	-	.	.	G	.	.	.	T	T	T	G	.	A	.	C	.	C
8:1	A	A	.	.	.	G	.	.	.	.	.	A	.	.	.	.	T	C	.	.	-	-	.	.	G	-	.	.	T	T	T	G	.	A	.	C	.	C
12:1	A	A	.	.	.	G	.	.	.	.	.	A	C	.	.	.	T	C	.	.	-	-	.	.	G	.	.	.	T	T	T	G	.	A	.	C	.	C
16:1	A	A	.	.	.	G	.	.	.	.	.	A	.	.	.	.	T	C	A	.	-	-	.	.	G	.	.	.	T	T	T	G	.	A	.	C	.	C

TER1+30Y

	3113994	3114036	3114236	**3114239**	3114280	3114344	**3114431**	3114480
1:21	A	A	T	A	T	-	T	G
2:10	.	.	.	G	G	A	A	.
3:3	G	.	.	.	.	-	.	.
4:2	.	.	.	.	.	A	A	.
5:3	.	G	A	G	G	A	A	.
6:1	.	.	.	.	.	A	A	A

TER1+100Y

	3194616	3194679	3194724	**3194949**	3195027
1:24	T	C	T	C	G
2:12	.	T	.	G	A
3:1	.	.	C	G	.
4:2	.	.	.	G	A
5:1	C	.	.	.	.

Figure S8 SNPs identified in the TER1 gene and in adjacent sequences. The figure illustrates the positions of the SNPs identified in the TER1 gene and the two adjacent sequences studied. Informative SNPs used in the linkage disequilibrium (Table 4) measurements are shown in bold.
